# TFAP2C Knockdown Sensitizes Bladder Cancer Cells to Cisplatin Treatment via Regulation of EGFR and NF-κB

**DOI:** 10.3390/cancers14194809

**Published:** 2022-09-30

**Authors:** Ji Xing, Wu Chen, Kang Chen, Shaoming Zhu, Fangyou Lin, Yucheng Qi, Yunlong Zhang, Shangting Han, Ting Rao, Yuan Ruan, Sheng Zhao, Weimin Yu, Fan Cheng

**Affiliations:** 1 Department of Urology, Renmin Hospital of Wuhan University, Wuhan 430060, China; 2 Department of Urology, Beijing Chao-Yang Hospital, Capital Medical University, Beijing 100020, China

**Keywords:** bladder cancer, TFAP2C, cisplatin, EGFR, NF-κB

## Abstract

**Simple Summary:**

Bladder cancer (BCa) is considered one of the most common neoplasms of the urology system. Cisplatin-based chemotherapy has been the primary treatment for patients with advanced or metastatic BCa. Nevertheless, cisplatin resistance often limits the treatment of bladder cancer. We expect to find approaches to improve the therapeutic efficacy of cisplatin in bladder cancer. In recent years, many studies have shown that transcription factor AP-2 gamma (TFAP2C) acts as a key player in cancer development and and its expression level is closely related to the sensitivity of tumors to cisplatin. Our study investigated whether TFAP2C affects the sensitivity of BCa cells to cisplatin and the possible mechanisms. We found that TFAP2C expression was significantly upregulated in most BCa tissues compared to adjacent normal tissues. The present study confirmed that TFAP2C knockdown enhanced the anti-tumor effects of cisplatin by decreasing cisplatin-induced activation levels of epidermal growth factor receptor (EGFR) and nuclear factor kappaB (NF-κB). Specifically, this study provides a novel approach to improve the efficacy of cisplatin.

**Abstract:**

Cisplatin is the first-line chemotherapy for advanced or metastatic bladder cancer. Nevertheless, approximately half of patients with BCa are insensitive to cisplatin therapy or develop cisplatin resistance during the treatment process. Therefore, it is especially crucial to investigate ways to enhance the sensitivity of tumor cells to cisplatin. Transcription factor AP-2 gamma (TFAP2C) is involved in cancer development and chemotherapy sensitivity. However, its relationship with chemotherapy has not been studied in BCa. In this study, we aimed to investigate the therapeutic potential of TFAP2C in human BCa. Results based on TCGA (The Cancer Genome Atlas), GTEx (The Genotype-Tissue Expression) and GEO (Gene Expression Omnibus) data showed that TFAP2C expression was upregulated in BCa tissues and that its high expression was associated with poor prognosis. Meanwhile, we demonstrated the overexpression of TFAP2C in BCa clinical specimens. Subsequently, in vitro, we knocked down TFAP2C in BCa cells and found that TFAP2C knockdown further increased cell cycle arrest and apoptosis caused by cisplatin. In addition, the inhibitory effect of cisplatin on BCa cell migration and invasion was enhanced by TFAP2C knockdown. Our data indicated that cisplatin increased epidermal growth factor receptor (EGFR) and nuclear factor-kappaB (NF-κB) activation levels, but TFAP2C knockdown suppressed this effect. Finally, in vivo data further validated these findings. Our study showed that TFAP2C knockdown affected the activation levels of EGFR and NF-κB and enhanced the anti-tumor effects of cisplatin in vivo and in vitro. This provides a new direction to improve the efficacy of traditional cisplatin chemotherapy.

## 1. Introduction

Bladder cancer (BCa) is one of the most common tumors of the urinary system, with an estimated incidence of more than 570,000 new cases in 2020 [1]. While considerable progress has been made in the treatment of BCa, patients still have unsatisfactory prognoses. Chemotherapy is an important modality for BCa treatment, and cisplatin-based chemotherapy has been the mainstay of treatment for patients with advanced or metastatic BCa for many years [2]. It is of great significance to reduce the BCa recurrence rate and mortality rate and improve the prognosis of patients. Nevertheless, the sensitivity to cisplatin varies among patients with BCa. The response rate of cisplatin chemotherapy in BCa is only approximately 40–60%, with a high recurrence rate [2]. Therefore, it is crucial to identify the mechanism of BCa resistance to cisplatin and improve the sensitivity of BCa to cisplatin.

Transcription factor AP-2 gamma (TFAP2C), also known as AP-2γ, belongs to the activator protein-2 (AP-2) transcription factor family. The activities of TFAP2C include involvement in DNA binding and regulation of transcriptional potential [3,4]. TFAP2C plays an indispensable role in embryonic development, and the human protein profile shows that TFAP2C is mainly distributed in the skin or esophagus [5,6,7]. Current studies have demonstrated that TFAP2C plays an important role in the regulation of cell proliferation, cell cycle progression and apoptosis, participating in the development of several cancers and influencing tumor sensitivity to chemotherapy [8,9,10,11]. In colorectal cancer (CRC), TFAP2C promotes chemoresistance by regulating the Hippo signaling pathway [12]. Additionally, in seminoma, TFAP2C enhances resistance to cisplatin chemotherapy [13]. However, whether and how TFAP2C affects the sensitivity of BCa to cisplatin has yet to be studied.

Activation of epidermal growth factor receptor (EGFR) by overexpression or mutation has been observed in a variety of human cancers, including BCa, which results in tumor cell proliferation, invasion, migration, and evasion of apoptosis [14,15,16]. In approximately 50% of bladder tumors, EGFR is strongly expressed, predicting muscle invasion and poor tumor differentiation [17,18]. Notably, EGFR inhibitors significantly reduced the resistance to cisplatin in cisplatin-resistant UCUB BCa cells [19]. Nuclear factor-kappaB (NF-κB) is activated in multiple types of cancers and associated with proliferation, apoptosis, and drug resistance [20,21]. In the absence of an activating stimulus, the prototypical NF-κB is present in the cytoplasm as a p65/p50 dimer and complexes with the NF-κB inhibitor kappa B(IκB) to form the NF-κB-IκB complex. Activation of NF-κB requires P65 to be freed and phosphorylated for nuclear translocation and binding to DNA sequences in the promoter regions of downstream target genes to regulate cellular processes [22]. Furthermore, NF-κB signaling is activated by cisplatin treatment in BCa cells and plays a critical role in cisplatin resistance and malignant behavior in BCa [23]. Dynamic crosstalk between EGFR- and NF-κB-dependent pathways has been widely observed in a variety of tumors [24]. However, the regulatory mechanisms of EGFR and NF-κB in BCa are not fully understood.

In this study, we observed that TFAP2C expression was significantly upregulated in most BCa tissues compared to adjacent normal tissues. Under cisplatin treatment conditions, TFAP2C knockdown led to lower levels of EGFR and NF-κB activation, enhancing the anti-tumor effect of cisplatin in BCa. Taken together, TFAP2C knockdown sensitized BCa cells to cisplatin treatment by regulating the EGFR and NF-κB signaling pathways.

## 2. Materials and Methods

### 2.1. Reagents and Antibodies

Cisplatin was purchased from MedChemExpress (MCE, Shanghai China) and dissolved in saline. EGF was purchased from PeproTech (Suzhou, China). The antibodies used in WB are shown in Table 1. The antibodies used in immunohistochemistry (IHC) are shown in Table 2. The antibodies used in immunofluorescence (IF) are shown in Table 3.

### 2.2. Bioinformatics

Pan-carcinoma gene expression data for the TCGA database [25] and the GTEx database [26] were obtained from the KMPLOT database (https://kmplot.com, accessed on 3 April 2022) [27]. The TFAP2C expression data in 407 tumors and 28 normal control tissues were obtained from the UCSC Xena database (for GTEx-bladder and TCGA-BLCA [28] data) (http://xena.ucsc.edu/, accessed on 14 May 2021). Association of TFA2PC expression with survival was analyzed on data from TCGA-BLCA dataset using KMplot (https://kmplot.com, accessed on 3 April 2022). In addition, the gene expression datasets and survival information of GSE dataset (GSE13507) [29] were downloaded from GEO (http://www.ncbi.nlm.nih.gov/geo/, accessed on 14 May 2021) [30]. The survival package in R software was used to conduct Kaplan-Meier survival analysis based on the data in the GSE13507 dataset. The expression of TFAP2C in BCa cell lines was obtained from CCLE (https://sites.broadinstitute.org/ccle/, accessed on 25 February 2021).

### 2.3. Tissue Samples

All BCa tissues and adjacent normal tissues were collected from patients who underwent surgery and were diagnosed with BCa at Renmin Hospital of Wuhan University. Tissue samples were frozen in liquid nitrogen before RNA extraction. All patients signed the informed consent form allowing their clinical materials to be used for research purposes. This study was approved by the Ethics Committee of Renmin Hospital of Wuhan University (approval no. WDRY2019-K035). The clinicopathological characteristics are shown in Table 4.

### 2.4. RNA Isolation and qRT–PCR

Total RNA was extracted from clinical specimens or cultured cell lines using TRIzol reagent (Bioshap, Hefei, China), and cDNA synthesis was performed using the Primescript RT reagent kit (Takara Bio Inc., Shiga, Japan). For mRNA analysis of cell lines, qRT-PCR was performed with a real-time PCR system (Biosystems, Thermo Fisher Scientific, Inc., Waltham, MA, USA), using SYBR Premix Ex Taq Reagent (Takara Bio Inc.). Clinical specimens were conducted using a real-time PCR system (Roche LightCycler 480 Instrument II system, Basel, Switzerland). qPCR cycling conditions (Biosystems): step 1: 95 °C 30 s step 2: GOTO (40 cycles) 95 °C (5 s) 60 °C (30 s); step 3: Melt Curve. qPCR cycling conditions (Roche LightCycler 480): step 1: 95 °C 30 s; step 2: 95 °C (5 s) 60 °C (30 s) 40 Cycles; step 3: 95 °C (5 s) 60 °C (60 s) 95 °C; step 4: 50 °C (30 s). The primers used for qRT–PCR were as follows: GAPDH forward, 5′-TGA CAT CAA GAA GGT GGT GAA GCA G-3′ and reverse, 5′-GTG TCG CTG TTG AAG TCA GAG GAG-3′; and TFAP2C forward, 5′-GAT CAG ACA GTC ATT CGC AAA G-3′ and reverse, 5′-AAG ACC TCA GTG GGG TTC ATT A-3′. The calculation of 2^−ΔΔCt^ for mRNA expression in cell lines was performed as previously described [31]. For clinical specimens, three wells were repeated for each sample and data analysis was performed using Lightcycler 480 software (version 1.5; Roche). 

### 2.5. Immunohistochemistry (IHC)

Tissue samples were fixed with formalin and embedded in paraffin. Antigens were retrieved by boiling in 0.01 M citrate buffer (pH 6.0), and endogenous peroxidase activity was quenched in 3% hydrogen peroxide. Nonspecific binding was blocked by goat serum followed by staining with the corresponding primary antibodies (Table 2). Next, sections were incubated with IgG (horseradish peroxidase (HRP)-conjugated) for 30 min, followed by incubation with DAB (Vector Laboratories, Newark, CA, USA). Finally, hematoxylin was used for counterstaining. The images were collected using microscopy (Olympus BX51; Olympus Corp, Tokyo, Japan). To quantify the IHC images, the integrated optical density (IOD)/Area was calculated using ImageJ software (version 1.52a; National Institutes of Health (NIH)) [32]. The calculation covered both the nucleus and the cytoplasm.

After collecting tumor tissues from nude mice, they were fixed in formalin, embedded in paraffin, and cut into tissue sections of 4 µm thickness. The subsequent steps were as described above.

### 2.6. Cell Culture

The BCa cell lines T24 (RRID: CVCL_0554) and 5637 (RRID: CVCL_0126) were purchased from The Cell Bank of Type Culture Collection of The Chinese Academy of Sciences and cultured in RPMI 1640 (HyClone; Cytiva) containing 10% fetal bovine serum (FBS; Hangzhou Sijiqing Biological Engineering Materials Company, Hangzhou, China) and 1% penicillin/streptomycin (Life Technology, Carlsbad, CA, USA). All cell lines were cultured in a humidified incubator at 37 °C with 5% CO_2_.

### 2.7. Cell Transfection

Knockdown lentiviruses for TFAP2C (termed shTFAP2C) and control lentivirus (termed shNC) were purchased from HanHeng Biology Company (Shanghai Province, China). Cells ready for transfection were seeded into 6-well dishes at a density of 1 × 106 cells/well and transfection was carried out according to the manufacturer’s instructions after reaching 40% confluence. Both 5637 and T24 cell lines were transfected with shTFAP2C or shNC. Stable transfected cell lines were obtained by using 2 μg/mL puromycin treatment. The stably transfected cells were used in subsequent experiments.

### 2.8. CCK-8 Cell Proliferation Assay and IC50 Determination

Cell proliferation assay: cells were counted using a TC20 automated cell counter (Bio-Rad) and seeded into 96-well plates at a density of 1 × 10^3^ (T24) cells/well or 3 × 10^3^ (5637) cells/well, with cisplatin treatment in advance for 24 h or no treatment. Cell proliferation was measured at 0, 24, 48, 72, and 96 h using the CCK-8 Assay Kit (Biosharp, Hefei, China). After incubation for 2 h at 37 °C, the absorbance at 450 nm was measured using a microplate reader (Ensight; Perkin Elmer, Waltham, MA, USA).

IC50 determination: cells were trypsinized and seeded into a 96-well plate at a density of 8 × 10^3^ cells/well, which was cultured in the incubator overnight. Subsequently, the cells were treated with various concentrations of cisplatin for 24 h. IC50 was measured via the CCK-8 Assay Kit (Biosharp, Hefei, China).

### 2.9. Transwell Cell Invasion Assay and Scratch Assay

The cisplatin-treated group was treated with cisplatin 24 h in advance. Cells (8 × 10^4^ cells) in 200 μL serum-free medium were seeded into the upper chamber, which was coated with Matrigel (BD Biosciences) for the invasion assays. Then, 600 μL of 10% FBS-containing medium was added to the bottom chamber. Then, the cells were incubated at 37 °C for 72 h, fixed with methanol for 30 min and stained with 0.1% crystal violet. Invasive cells were counted by a microscope (Olympus IX71; Olympus Corp., Tokyo, Japan) from 3 randomly selected fields.

Cells were seeded into 6-well plates at a density of 1 × 10^6^ cells/well. When the cells reached 100% confluence, a scratch was created using a 200 µL pipette tip. Images of the wound were captured at 0 and 24 h using a microscope (Olympus IX71; Olympus Corp.). The healing rate (%) was calculated as follows: (0 h scratch width–24 h scratch width)/0 h scratch width × 100%.

### 2.10. Colony-Formation Assay

After advance cisplatin treatment for 24 h in the cisplatin-treated group, cells were seeded into 6-well plates (500 cells/well) to conduct the colony formation assay. After incubation for 2 weeks, the cells were fixed in paraformaldehyde for 30 min at room temperature and stained with 0.1% crystal violet, and the number of colonies was counted manually.

### 2.11. Immunofluorescence Staining

Cells were seeded into a 6-well plate with coverslips at a density of 5 × 10^4^ cells/well. After reaching 30% confluence, the cisplatin-treated group experienced 24 h of cisplatin treatment. Cells were fixed for 15 min with 4% paraformaldehyde, permeabilized with 0.5% Triton X-100 and then blocked with 1% bovine serum albumin V for 1 h. Primary antibodies (Table 3) were incubated at 4 °C overnight. After this step, the cells were incubated with the corresponding secondary antibody at room temperature for 1 h. Finally, the cells were counterstained with DAPI, and the coverslip was mounted on slides before imaging. A fluorescence microscope (Olympus IX71; Olympus Corp) was used to obtain fluorescence images.

### 2.12. Western Blot Analysis

Cells were lysed in whole-cell lysis buffer (Radioimmunoprecipitation Assay (RIPA) buffer (Beyotime)), and protein concentrations were quantified using a bicinchoninic acid (BCA) protein assay kit (Beyotime). Total protein was separated by SDS–PAGE and transferred to a polyvinylidene fluoride (PVDF) membrane. The membrane was blocked with 5% skim milk for 2 h at room temperature and incubated with the corresponding primary antibodies (Table 1) at 4 °C overnight. After three washes with Tris-buffered saline-Tween 20, membranes were incubated with horseradish peroxidase-conjugated anti-rabbit or anti-mouse secondary antibodies. Protein bands were detected using a chemiluminescence system (ChemiDoc^TM^ Touch; Bio-Rad, Hercules, CA, USA) and analyzed using ImageJ software (version 1.52a; National Institutes of Health (NIH)). The experiment was repeated three times for each group. Original blots see Supplementary File S1.

### 2.13. Cell Cycle and Apoptosis Assay

Cells were seeded in 6-well plates (1 × 10^6^ cells/well). After 24 h of treatment or nontreatment with cisplatin, cells were digested using trypsin and then incubated with an Annexin V-APC/PI Apoptosis Detection Kit (Elabscience) at room temperature for 30 min. Apoptosis was assessed through flow cytometry (CytoFlex; Beckman Coulter Life Sciences) and analyzed by FlowJo software (version 10; FlowJo). For cell cycle analysis, cells were stained with propidium iodide (PI) for 15 min according to the kit protocol and analyzed by flow cytometry as previously described.

### 2.14. In Vivo Xenograft Experiments In Vivo

Four -week-old nude mice were obtained from the Animal Experiment Center of Wuhan University. 5637 cells (2 × 10^6^) stably transfected with TFAP2C knockout lentivirus (shTFAP2C) or control lentivirus (shNC), were subcutaneously injected into each mouse. From day 7 after tumor cell inoculation, mice in the cisplatin-treated group were given weekly intraperitoneal injections of cisplatin (2.5 mg/kg). In the remaining groups without cisplatin treatment, the mice were administered an isovolumetric injection of normal saline. The growth of tumor size (L, longest dimension; W, shortest dimension) was monitored by the caliper rule every 5 days, and tumor volumes were calculated using the formula: V = L × W × W/2 [33]. The tumors were subsequently harvested for further research.

### 2.15. TUNEL Detection

The TUNEL assay was performed according to the manufacturer’s protocol using the In Situ Apoptosis Detection kit (Roche Applied Science). Apoptotic cell nuclei were stained brown, and negative cell nuclei were stained blue. TUNEL assay was used to detect apoptosis in the tumor samples from nude mice.

### 2.16. Statistical Analysis

All data were analyzed with SPSS software (version 26.0, SPSS Inc., Chicago, IL, USA). All figures were created with GraphPad Prism software (version 9.0; GraphPad Software). The Shapiro‒Wilk test and Kolmogorov‒Smirnov test were used to assess normality. Paired t-tests were used to compare the RT-qPCR data from the patients’ tumor tissues and adjacent normal tissues. An independent-sample t test was used to analyze potential differences between the two groups. The tumor volumes of nude mice in each group were compared using analyses of variance with repeated measures. Multivariate analysis of variance was applied to analyze effects of different factors. After analyses of variance with repeated measures and Multivariate analysis of variance, post hoc analyses were performed using the Tukey–Kramer test. Unless otherwise stated, the data represent the mean ± standard deviation (SD) of three independent experiments (* *p* < 0.05, ** *p* < 0.01, *** *p* < 0.001, ns, not significant).

## 3. Results

### 3.1. TFAP2C Is Highly Expressed in Tumor Tissues and Is Associated with Patient Prognosis

**Figure 1:**
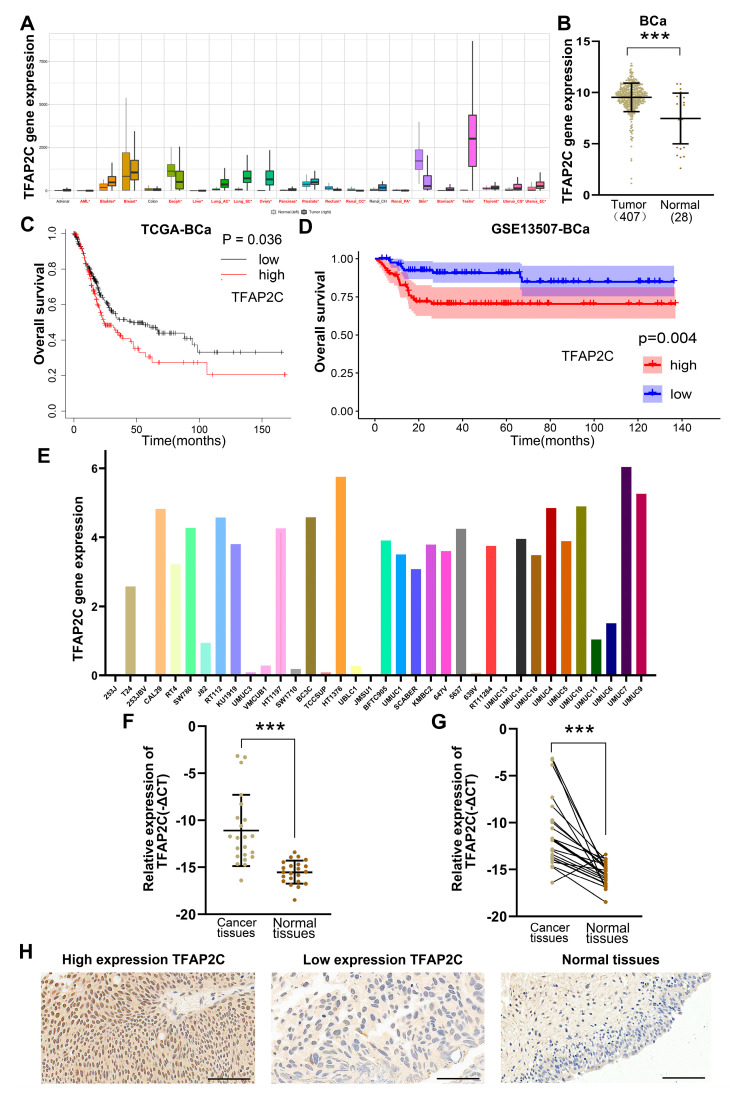
TFAP2C expression analysis and cancer prognosis. (**A**) The expression of TFAP2C in pan − carcinoma tissues. Tumor names marked in red represent significant differences. (**B**) TFAP2C expression in tumor and normal tissues in TCGA − BLCA dataset and GTEx datasets. (**C**,**D**) Kaplan − Meier plot curves of the TCGA − BLCA dataset (**C**) and GSE dataset (GSE13507) (**D**). (**E**) The expression of TFAP2C in 36 BCa cell lines. (**F**,**G**) qRT − PCR was performed to measure TFAP2C mRNA expression levels in 22 pairs of BCa and adjacent normal tissues. TFAP2C expression was significantly upregulated in BCa tissues compared with adjacent normal tissues. (**H**) Representative immunohistochemistry of TFAP2C in BCa tissues and adjacent tissues. Scale bars, 100 μm. (*** *p* < 0.001).

By using the KMPlot database, we first aimed to determine TFAP2C expression in pan-cancerous tissues (for GTEx and TCGA data). The results indicated that the expression of TFAP2C was significantly different in tumor tissues compared to the corresponding normal tissues in 19 different cancers (Figure 1A). We found that TFAP2C was highly expressed in lung cancer, prostate cancer, and BCa, among others. However, it was expressed at low levels in skin cancer. To show the expression of TFAP2C in human BCa more clearly, we compared TFAP2C mRNA levels between tumor tissues (*n* = 407) and normal tissues (*n* = 28) using the UCSC Xena database (for GTEx-bladder and TCGA-BLCA data) (Figure 1B). We further analyzed the correlation between TFAP2C expression and the survival condition of BCa patients in independent databases (TCGA-BLCA dataset (analyzed using KMplot) and GSE dataset (GSE13507)). For overall survival (OS), higher TFAP2C expression was associated with a shorter OS of BCa patients in the TCGA-BLCA dataset (Figure 1C) and GSE databases (Figure 1D). Moreover, the expression of TFAP2C mRNA in 36 BCa cell lines was determined via CCLE analysis (Figure 1E). The T24 and 5637 cell lines were selected for further analysis, as they were used frequently and had high expression of TFAP2C among the BCa cell lines. Then, the mRNA levels of TFAP2C in 22 pairs of BCa tissues and corresponding adjacent normal tissues were examined. The results demonstrated that TFAP2C was significantly upregulated in BCa tissues compared with adjacent normal tissues (Figure 1F,G). To further verify the differential expression of TFAP2C in BCa, six pairs of BCa tissues and corresponding adjacent normal tissues were randomly selected for immunohistochemical analysis. (Figure 1H, The results of the IHC analysis were presented in Supplementary Material Figure S1). Our results suggested that TFAP2C expression was primarily detected within the cytoplasm and nucleus, but occurred mostly in the nucleus, and its expression was higher than that in adjacent tissues (5 of 6 cases had higher expression in tumor tissues than in adjacent normal tissues).

### 3.2. TFAP2C Knockdown Enhances the Inhibitory Effect of Cisplatin on BCa Cells Proliferation In Vitro

**Figure 2:**
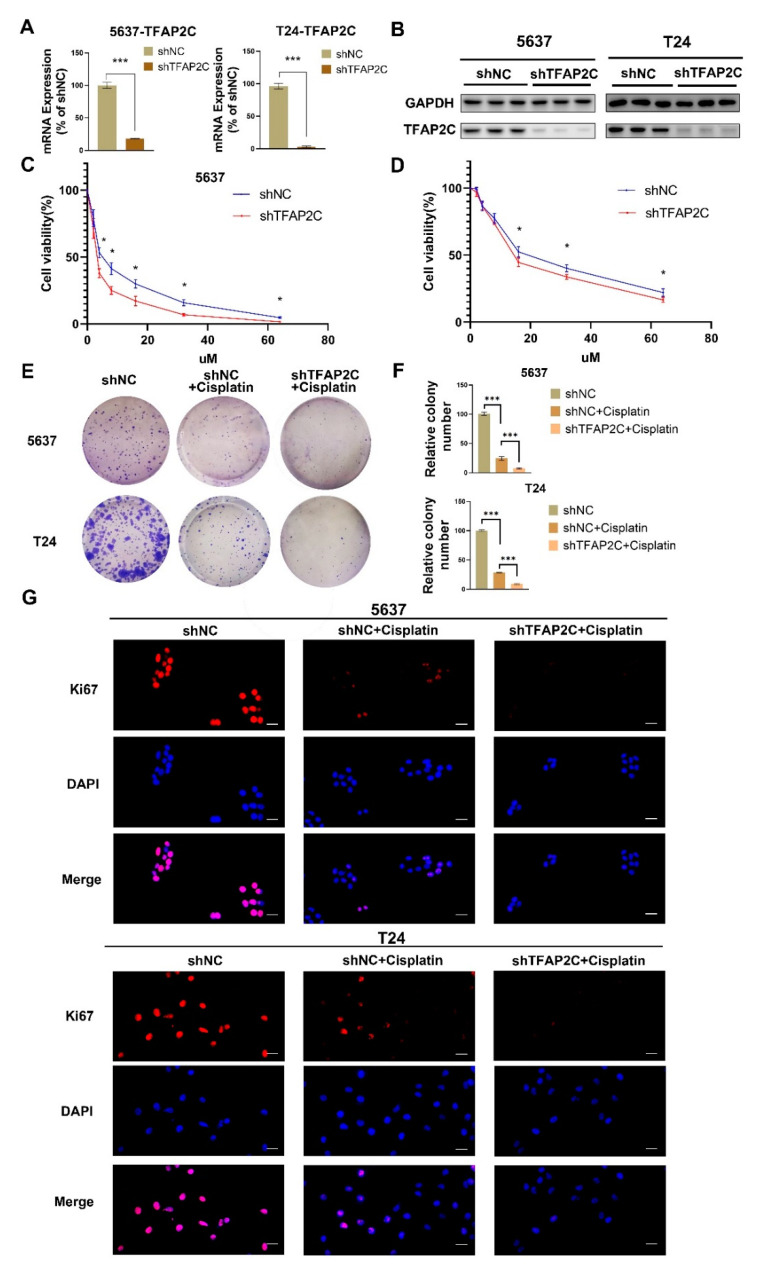
TFAP2C knockdown enhances the anti-tumor effects of cisplatin. (**A**,**B**) The knockdown efficiency of TFAP2C was confirmed by qRT–PCR and WB analysis. (**C**,**D**) CCK-8 assays were performed to detect cell viability following treatment with cisplatin. TFAP2C knockdown increased the suppression rate of cisplatin in 5637 and T24 cells. (**E**,**F**) Representative images of the colony formation assay. (**G**) Representative immunofluorescence images of Ki67 staining in 5637 cells and T24 cells. Scale bars, 20 μm. Data represent the mean ± standard deviation (SD) of three independent experiments (* *p* < 0.05, *** *p* < 0.001).

To assess whether the expression level of TFAP2C affects the sensitivity of BCa cells to cisplatin, T24 and 5637 cells were used to construct 2 stable cell lines with stable TFAP2C knockdown, and knockdown efficiency was assessed by qRT-PCR and WB (Figure 2A,B). Subsequently, increasing concentrations of cisplatin were used to treat BCa cells. Determination of IC50 was assayed by CCK-8 assay. The results demonstrated that compared with shNC group, BCa cells in the shTFAP2C knockdown group had significantly lower viability (Figure 2C,D). For the shNC group in 5637 and T24 cells, fitted values were IC50 = 5.75 µM (95% confidence interval; 5.51–6.50 µM) and 20.87 µM (95% confidence interval; 18.93–23.05 µM), respectively. For the shTFAP2C group in 5637 and T24 cells, fitted values were IC50 = 3.397 µM (95% confidence interval; 2.984–3.829 µM) and 17.13 µM (95% confidence interval; 15.86–18.53 µM), respectively. Based on IC50 data, we selected 5 μM and 20 μM cisplatin in 5637 and T24 cells, for subsequent studies, respectively. Colony-formation assay was used to assess the proliferation ability of BCa cells. The results showed that the colony-forming ability of BCa cells was inhibited by cisplatin and would be further inhibited by cisplatin combined with TFAP2C knockdown (Figure 2E,F). Next, immunofluorescence was used to measure the proliferation marker Ki67. The results showed that cisplatin inhibited Ki67 expression in BCa cells, and cisplatin combined with TFAP2C knockdown further inhibited Ki67 expression (Figure 2G).

### 3.3. TFAP2C Knockdown Enhances the Inhibitory Effect of Cisplatin on BCa Cells Migration and Invasion In Vitro

**Figure 3:**
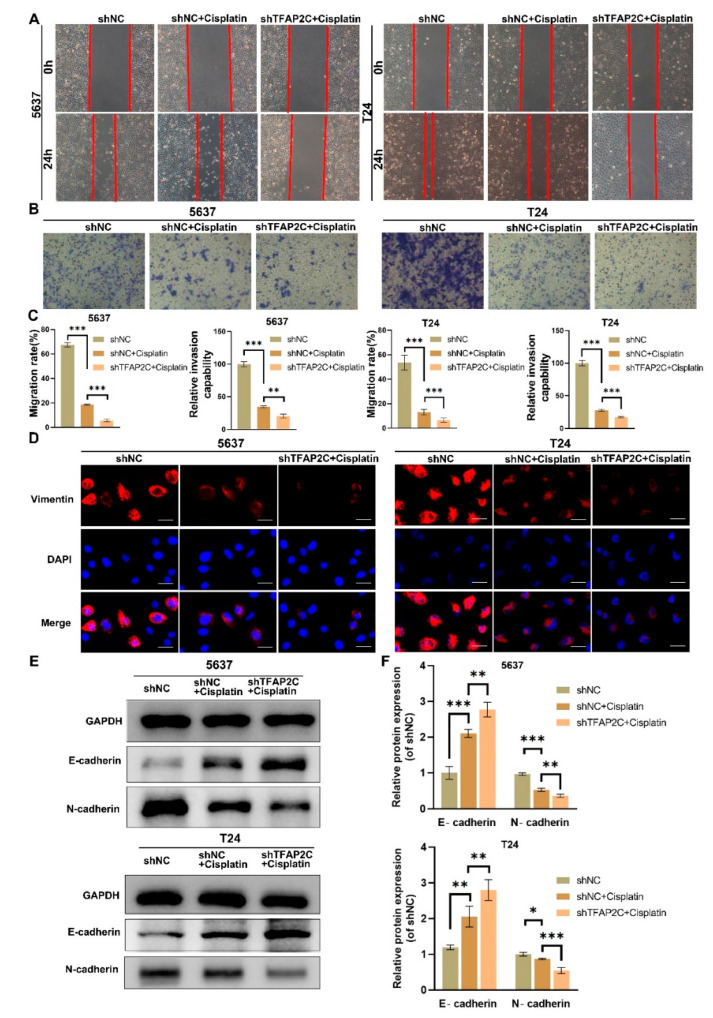
TFAP2C knockdown enhances the inhibitory effect of cisplatin on migration and invasion. (**A**–**C**) Representative images of the scratch assay in 5637 and T24 cells. Inhibition of migration by cisplatin is enhanced by TFAP2C knockdown. Representative images of the transwell assay. Inhibition of invasion by cisplatin is enhanced by TFAP2C knockdown (**D**) Representative images of vimentin immunostaining. Scale bars, 20 μm. (**E**,**F**) Protein expression levels of E-cadherin and N-cadherin. Data represent the mean (±standard deviation, SD) of three independent experiments (* *p* < 0.05, ** *p* < 0.01, *** *p* < 0.001).

The transwell cell invasion assay and scratch assay were used to detect the effects of cisplatin and TFAP2C knockdown on BCa cells invasion and migration abilities. The results showed that compared with shNC+cisplatin group, both the healing rate and the number of invasive cells of BCa cells were significantly decreased in the shTFAP2C+cisplatin group, indicating that 5637 and T24 cells in the shTFAP2C+cisplatin group had lower migratory and invasive abilities (Figure 3A–C). Next, immunofluorescence and WB were used to assess the level of epithelial markers and mesenchymal markers. The results showed that cisplatin inhibited EMT in 5637 and T24 cells, which was shown with a rise in epithelial marker (E-cadherin) and a decrease in mesenchymal markers (vimentin and N-cadherin). Unsurprisingly, the inhibitory effect of cisplatin on EMT was enhanced after TFAP2C was knocked down. (Figure 3D–F).

### 3.4. TFAP2C Knockdown Enhances Cisplatin-Induced Apoptosis In Vitro

**Figure 4:**
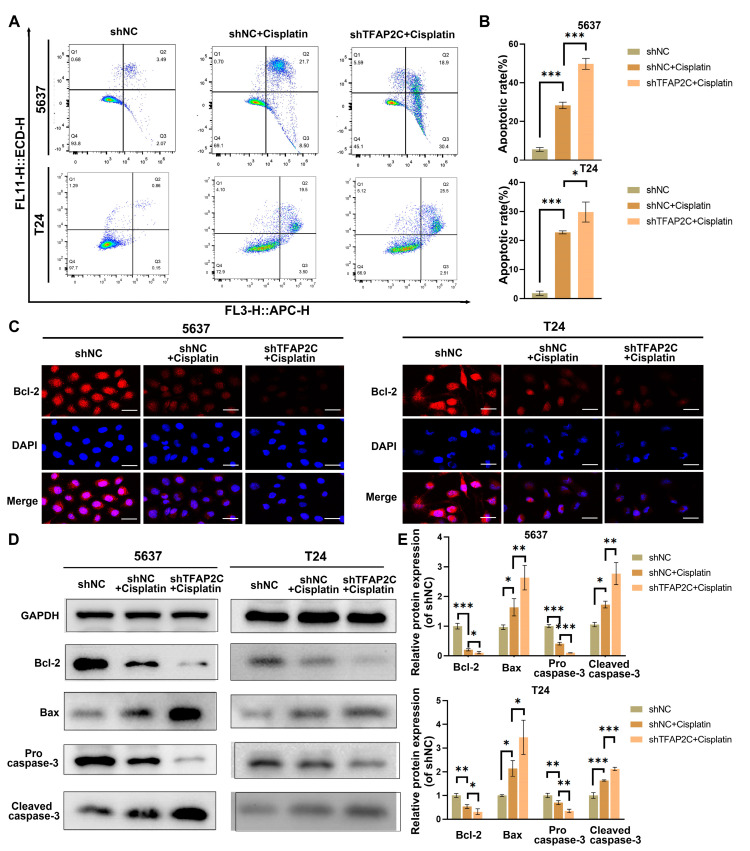
TFAP2C knockdown enhances cisplatin-induced apoptosis. (**A**,**B**) Flow cytometry results of apoptosis in T24 and 5637 cells. (**C**) Representative images of Bcl-2 immunostaining. Scale bars, 20 μm. (**D**,**E**) Protein expression levels of Bcl-2, Bax, pro-caspase-3, and cleaved caspase-3. Data represent the mean ± standard deviation (SD) of three independent experiments (* *p* < 0.05, ** *p* < 0.01, *** *p* < 0.001).

To assess the effect of TFAP2C knockdown on cisplatin-induced apoptosis, flow cytometry was used to detect the rate of apoptosis. The results showed that compared to the shNC+cisplatin group, TFAP2C knockdown increased cisplatin-induced apoptosis in 5637 and T24 cells (Figure 4A,B). In addition, immunofluorescence or WB was used to analyze the levels of Bax, Bcl-2, pro-caspase-3, and cleaved caspase-3. The results showed that compared with the shNC+cisplatin group, the expression of cleaved caspase-3 and Bax was upregulated in the shTFAP2C+cisplatin group, whereas the expression of Bcl-2 and pro caspase-3 was downregulated. (Figure 4C–E). In conclusion, apoptosis induced by cisplatin was enhanced by TFAP2C knockdown.

### 3.5. TFAP2C Knockdown Enhances Cisplatin-Induced Cell Cycle Arrest In Vitro

**Figure 5:**
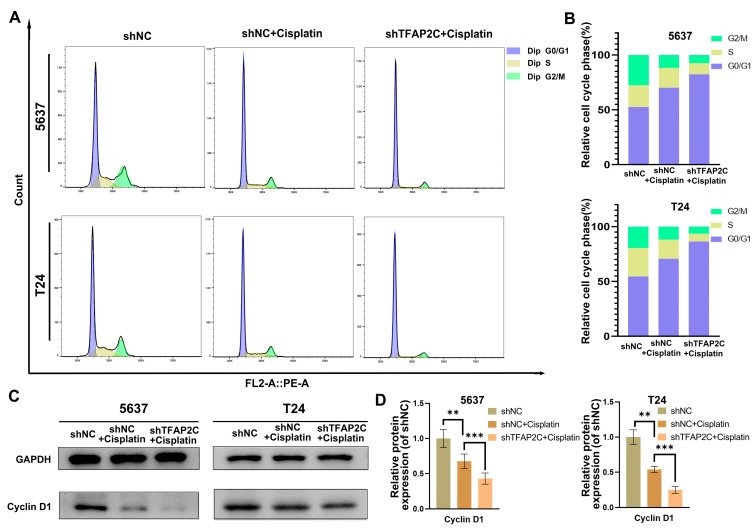
TFAP2C knockdown enhances cisplatin-induced cycle arrest. (**A**,**B**) Flow cytometry results of the cell cycle in T24 and 5637 cells. (**C**,**D**) Protein expression levels of cyclin D1. Data represent the mean ± standard deviation (SD) of three independent experiments (** *p* < 0.01, *** *p* < 0.001).

To investigate whether the lower proliferative capacity of BCa cells in the shTFAP2C+cisplatin group was associated with cell cycle arrest, cell cycle distribution was determined using Flow cytometry. The results showed that the ratio of G0/G1 phase cells in the shTFAP2C+cisplatin group was higher than that in the shNC+cisplatin group (Figure 5A,B). This indicated that cisplatin-induced G1 phase arrest of BCa cells was enhanced by TFAP2C knockdown. Furthermore, the expression level of cyclin D1, which drives the cells from G1 to S phase, was examined using WB. The results showed that cisplatin decreased the expression of cyclin D1 and that cisplatin combined with TFAP2C knockdown further reduced its expression (Figure 5C,D).

### 3.6. TFAP2C Knockdown Inhibits Cisplatin-Induced NF-κB and EGFR Activation In Vitro 

**Figure 6:**
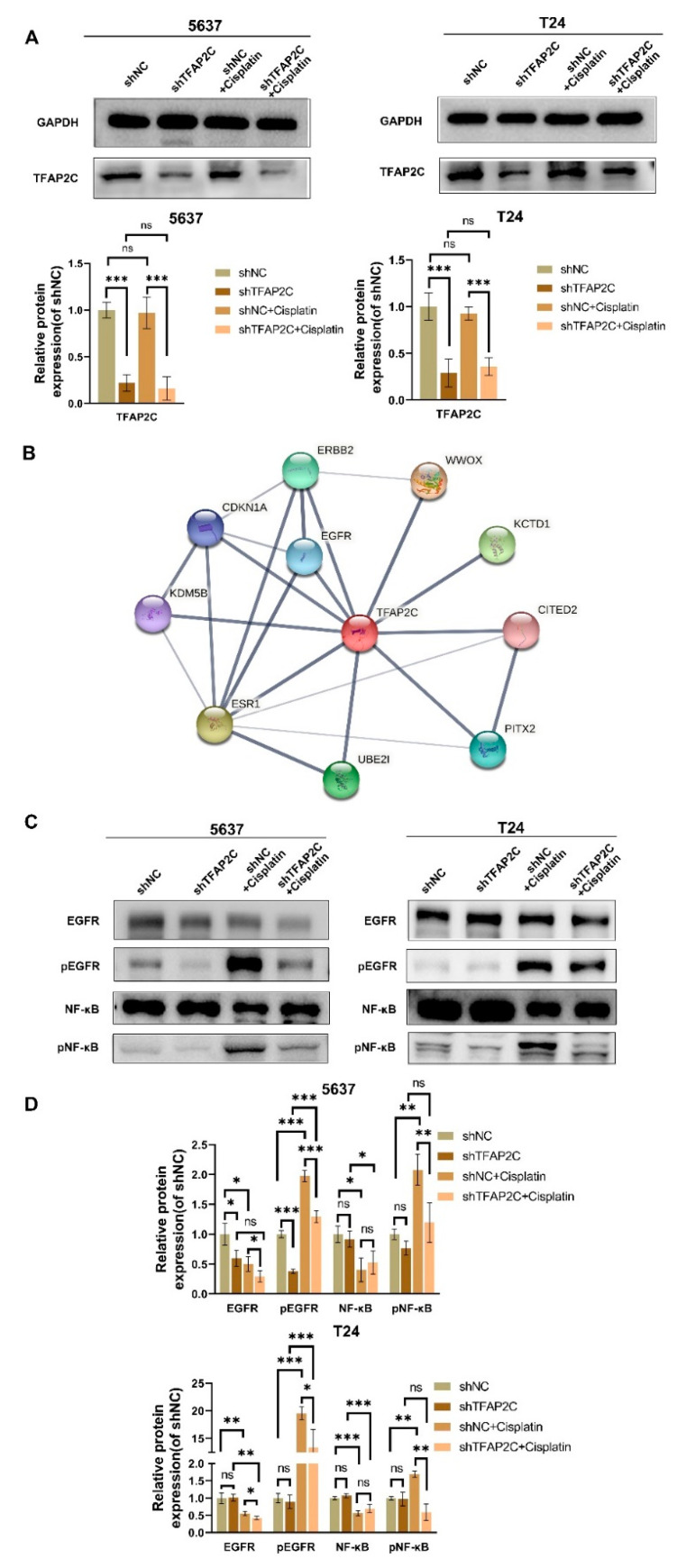
Activation of EGFR and NF-κB induced by cisplatin is affected by TFAP2C. (**A**) The protein expression level of TFAP2C in T24 and 5637 cells. The TFAP2C protein expression level was not increased by cisplatin. (**B**) The results from the STRING database revealed the protein interaction partners for TFAP2C. (**C**,**D**) Protein expression levels of EGFR, pEGFR, NF-κB, and pNF-κB. Data represent the mean (± standard deviation, SD) of three independent experiments (* *p* < 0.05, ** *p* < 0.01, *** *p* < 0.001, ns, not significant).

**Figure 7:**
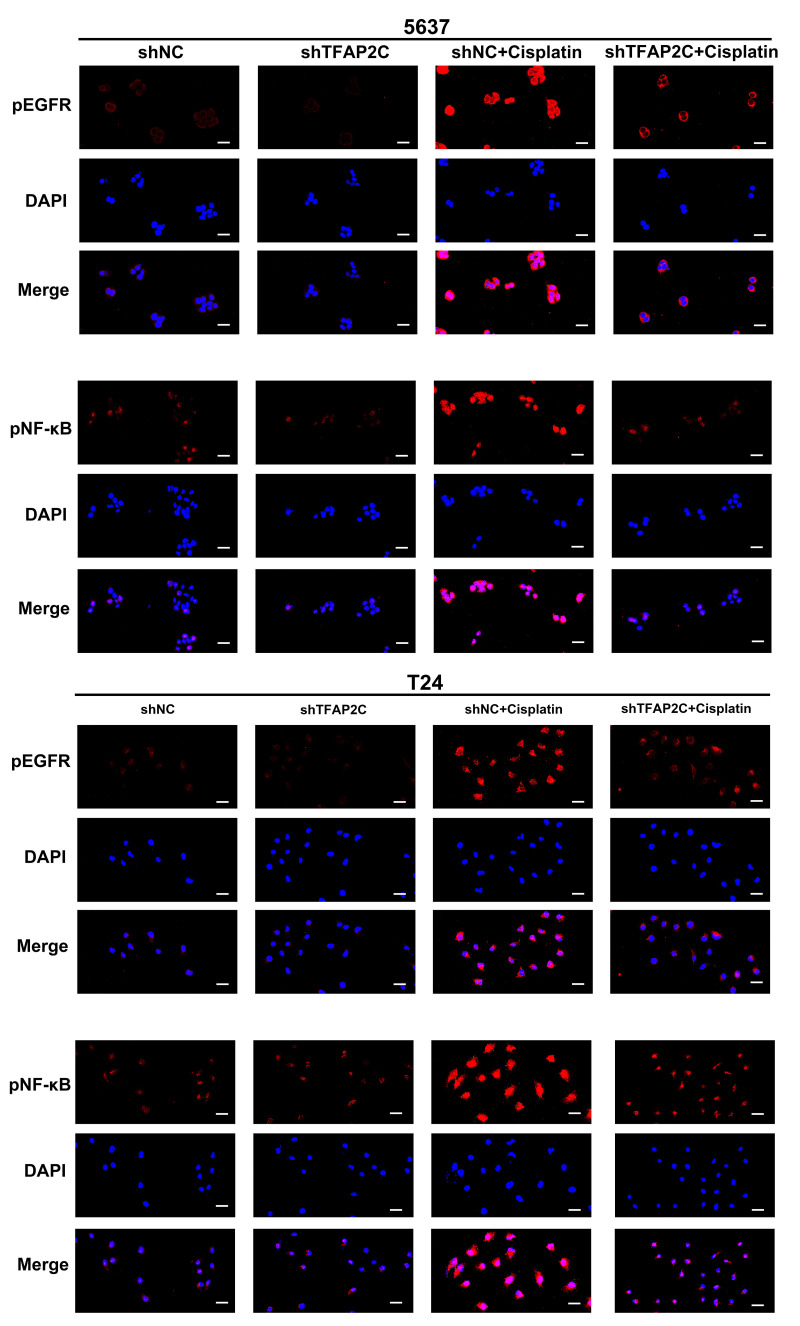
Representative images of pEGFR and pNF-κB immunostaining. Scale bars, 20 μm.

We delved into the possible molecular mechanisms by which TFAP2C knockdown affected the effect of cisplatin treatment in BCa. First, whether TFAP2C expression was affected by cisplatin was analyzed by WB. The results showed that the expression of TFAP2C was not changed by cisplatin (Figure 6A). Next, the STRING website (https://string-db.org/, accessed on 3 June 2021) was used to search for the possible targets of TFAP2C (Figure 6B). Among the potential targets of TFAP2C, EGFR attracted our attention due to its close association with the cisplatin sensitivity in BCa [34]. Moreover, we assessed the activation of NF-κB as NF-κB promotes BCa cell survival in cisplatin treatment and can be activated by EGFR [23,35,36,37]. WB results showed that cisplatin enhanced the activation of EGFR and NF-κB, and TFAP2C knockdown suppressed this phenomenon (Figure 6C,D). Simultaneously, decreased EGFR and NF-κB levels were observed after EGFR and NF-κB activation. The possible mechanism was that massive amounts of EGFR and NF-κB were phosphorylated. Immunofluorescence of pEGFR and pNF-κB further confirmed this result (Figure 7). In addition, knockdown of TFAP2C alone also resulted in reduced activation of EGFR in 5637 cells, but not significantly in T24 cells. These findings suggested that TFAP2C was a key factor in the activation of EGFR by cisplatin in BCa cells.

### 3.7. TFAP2C Affects the Sensitivity of BCa Cells to Cisplatin by Regulating the Activation of EGFR In Vitro

**Figure 8:**
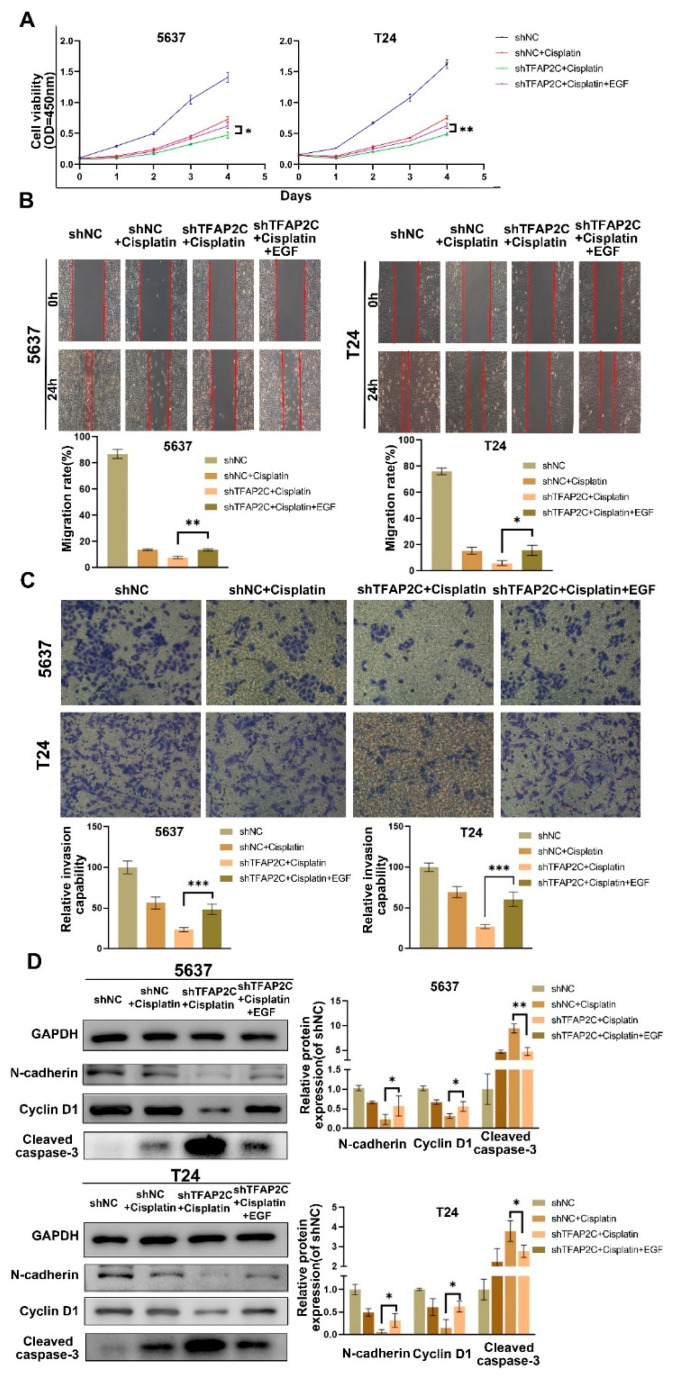
Activation of EGFR with EGF partially reduced the sensitivity of BCa cells to cisplatin. (**A**) Cell viability CCK-8 assay. (**B**) Representative images of the Scratch Assay in 5637 and T24 cells. (**C**) Representative images of the transwell assay. (**D**) Protein expression levels of E-cadherin, cyclin D1, and cleaved-caspase-3. Data represent the mean ± standard deviation (SD) of three independent experiments (* *p* < 0.05, ** *p* < 0.01, *** *p* < 0.001).

To further investigate whether TFAP2C affects the sensitivity of BCa cells to cisplatin by decreasing the level of EGFR activation, we activated EGFR with EGF before cells underwent 24 h of cisplatin treatment. Subsequently, proliferation was measured by CCK8, migration ability was determined by scratch assay, and invasion ability was assayed by transwell assay. The results revealed that the proliferation, migration, and invasion abilities of cells in the shTFAP2C+cisplatin group were partially reversed after EGF treatment (Figure 8A–C). Moreover, the result of WB showed the expression levels of N-cadherin and cyclin D1 were increased after EGF treatment, while the levels of cleaved caspase-3 were decreased (Figure 8D). This confirmed that activation of EGFR could partially reduced the effect of TFAP2C knockdown on cisplatin sensitivity in BCa cells. 

### 3.8. TFAP2C Enhances the Anti-Tumor Effect of Cisplatin In Vivo

**Figure 9:**
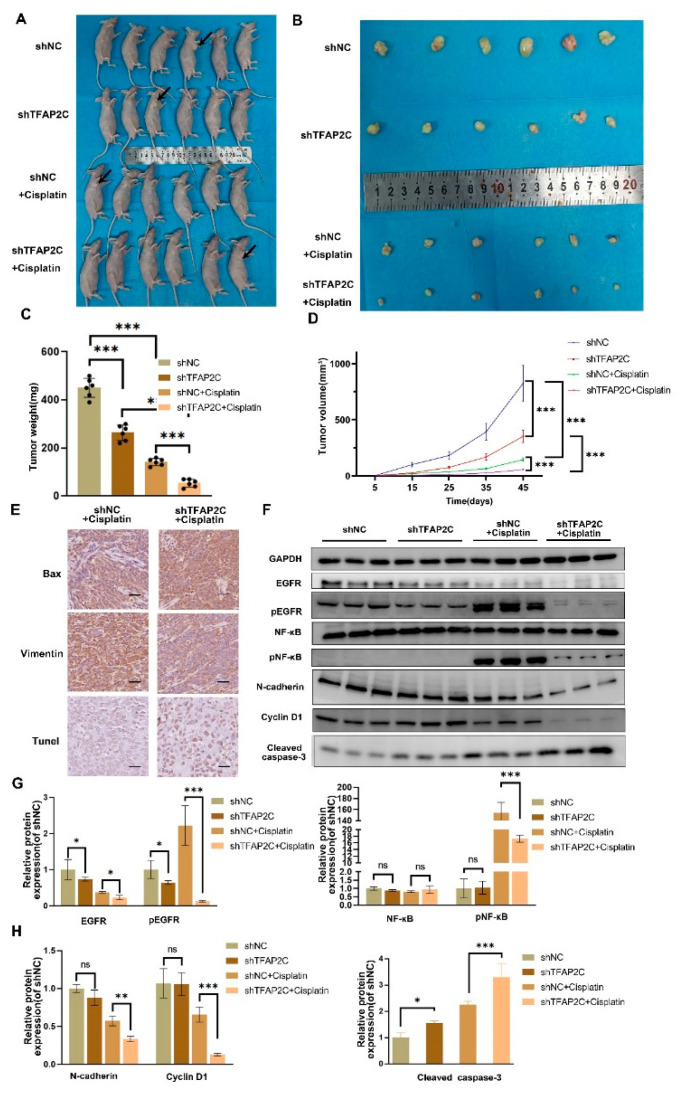
TFAP2C knockdown alone or combined with cisplatin inhibits BCa cell growth in vivo. (**A**) Images of tumors in nude mice after different treatments. Arrows indicate the subcutaneous tumors. (**B**) Morphology of the subcutaneously implanted tumor. (**C**) Tumor weight. (**D**) Volume of tumor at each time point. (**E**) Representative images of TUNEL staining and IHC staining for Bax and vimentin in the cisplatin alone and cisplatin combined with TFAP2C knockdown groups. Scale bars, 20 μm. (**F**–**H**) Expression levels of major proteins in different groups. Data represent the mean ± standard deviation (SD) of three independent experiments (* *p* < 0.05, ** *p* < 0.01, *** *p* < 0.001, ns, not significant).

To demonstrate the function of TFAP2C in vivo, nude mice were subcutaneously injected with shNC or shTFA2PC 5637 cells. This was followed by cisplatin treatment or no treatment. We found that cisplatin treatment significantly inhibited tumor growth and that shTFAP2C knockdown also inhibited tumor growth (Figure 9A–D). Multifactor ANOVA results showed TFAP2C knockdown and cisplatin treatment had a synergistic effect on tumor suppression. These results suggested that cisplatin treatment inhibited tumor growth in vivo and TFAP2C knockdown enhanced the inhibitory effect of cisplatin. The TUNEL results showed that compared with the shNC+cisplatin group, tumors in the shTFAP2C+cisplatin group showed an increased number of apoptotic (TUNEL-positive) cells. IHC showed that compared to the shNC+cisplatin group, tumors in the shTFAP2C+cisplatin group had increased Bax expression and decreased Vimentin expression (Figure 9E, The results of the IHC analysis were presented in Supplementary Material Figure S2). The expression levels of EGFR, pEGFR, NF-κB, pNF-κB, N-cadherin, cyclin D1, and cleaved caspase-3 were detected by WB and the results were consistent with in vitro data (Figure 9F–H). All these results further demonstrated that TFAP2C affected the sensitivity of BCa cells to cisplatin through EGFR and NF-κB. 

## 4. Discussion 

BCa is one of the most common cancers worldwide and caused more than 210,000 deaths in 2020 [1]. Despite the development of new therapeutic approaches, cisplatin-based chemotherapy remains the first-line treatment for muscle-invasive BCa [38,39,40,41]. However, chemoresistance is one of the obstacles to cisplatin chemotherapy for BCa, resulting in treatment failure [42,43,44]. Therefore, it is particularly important to investigate the mechanism of cisplatin resistance in BCa. The aim of this study was to explore the impact of TFAP2C on the anti-tumor effects of cisplatin in BCa. Our study found cisplatin increased apoptosis and inhibited the proliferation and migration of BCa, and TFAP2C knockdown enhanced this effect of cisplatin by decreasing the activation levels of EGFR and NF-κB. 

The anti-tumor activity of cisplatin involves multiple mechanisms, including causing tumor cell mitochondrial apoptosis, inhibiting tumor cell proliferation and metastasis [45,46,47]. Under a normal environment, tumor cells enhance metastasis via the EMT process, which is accompanied by the transformation of immotile epithelial cells into motile mesenchymal cells, with downregulation of epithelial markers (such as E-cadherin) and upregulation of mesenchymal markers (such as vimentin and N-cadherin) [48,49]. Meanwhile, sustained proliferation is a hallmark of cancer cells, concurrent with cell cycle dysregulation [50,51,52]. Under cisplatin treatment, mitochondrial apoptosis is induced by oxidative stress and DNA damage that results in cell death [46,53,54,55,56]. In addition, cisplatin decreases tumor metastasis and inhibits tumor proliferation by suppressing the EMT process and arresting the cell cycle [46,57]. This is consistent with our data. Cisplatin increased the rate of apoptosis in BCa cells, downregulated Bcl-2, upregulated Bax and activated caspase-3. In addition, cisplatin inhibited proliferation by inducing G1 phase arrest in BCa cells and suppressed BCa migration by suppressing the EMT process. These results were supported by in vivo data.

Although numerous studies have been conducted, the mechanisms leading to cisplatin resistance are not completely understood. It has been reported that transcription factors, including activating transcription factor-2 (ATF2), ATF4, and AP-2, may be associated with cisplatin resistance gene activation [58,59,60]. TFAP2C is a member of the AP-2 family, whose functional activities include involvement in DNA binding and regulation of transcriptional potential [61,62]. Interestingly, TFAP2C exhibits pro- and anti-tumor functions in different tumors, depending on the function and tumor type [11,63,64,65]. Furthermore, Wei et al. also confirmed in seminoma that TFAP2C affects the efficacy of cisplatin [13]. To date, there are few reports on the role of TFAP2C in BCa. In this study, high expression of TFAP2C in BCa was observed in the TCGA-BLCA dataset and GTEx database, which correlated with poor prognosis of BCa patients. We verified the high expression of TFAP2C in BCa by 22 pairs of BCa tissues and adjacent normal tissues. The increased expression of TFAP2C supported that TFAP2C has a pro-tumor function in BCa. Next, to investigate the effect of TFAP2C expression level on cisplatin treatment of BCa, we compared the therapeutic effects of cisplatin on BCa cells both before and after TFAP2C knockdown. We observed enhanced therapeutic effects of cisplatin on BCa after TFAP2C knockdown, including increased BCa apoptosis and decreased BCa migration and proliferation. Finally, Xenograft experiments demonstrated that TFAP2C knockdown also enhanced the anti-tumor effect of cisplatin in vivo.

Next, we further explored the possible mechanisms by which TFAP2C affects the therapeutic efficacy of cisplatin. We used the STRING website to search for potential targets of TFAP2C. EGFR is a member of the ERBB family of receptor tyrosine kinases (RTKs), which crucially promotes cell survival, proliferation, and invasiveness [66]. EGFR dimerization and phosphorylation of multiple tyrosines occur during the activation of EGFR and specifically trigger PI3K/AKT/mTOR, NF-κB, STAT, and RAS/RAF/MEK1/ERK1/2 activation [24,67,68]. The activation of EGFR induced by cisplatin has been observed in a variety of cancers, and the extent of its activation may affect the sensitivity of cancer cells to chemotherapy [19,37,69,70]. Moreover, the activation of NF-κB induced by cisplatin led to cisplatin resistance, which has been demonstrated in BCa [23,71]. Indeed, this is similar to our observed data that cisplatin induced the activation of EGFR and NF-κB. Our evidence also demonstrates that cisplatin-induced activation levels of EGFR and NF-κB were significantly reduced after TFAP2C knockdown and that EGFR protein levels were also reduced in accordance with attenuated TFAP2C expression in the cisplatin-treated groups. This is consistent with the findings of De Andrade et al. and Park et al. that TFAP2C regulates the expression of EGFR [10,72]. We speculate that TFAP2C influences the response of BCa cells to cisplatin by regulating EGFR levels and activation. Based on those results, we activated EGFR and observed that after TFAP2C knockdown, activation of EGFR partially reduced the sensitivity of Bca cells to cisplatin. The subsequent in vivo experiments also showed that TFAP2C knockdown led to a decrease in cisplatin-induced EGFR levels and further inhibited tumor growth. The above data further supports our speculation.

Although our study showed TFAP2C as a potential target to improve the efficacy of cisplatin, our study still had some shortcomings. Due to the difficulty in collecting clinical specimens, the expression levels of TFAP2C before and after cisplatin treatment were not studied. In addition, the mechanism was not further validated in in vivo experiments by using EGFR activators. However, this did not affect our final conclusion that TFAP2C knockdown improved the sensitivity of BCa to cisplatin.

**Figure 10:**
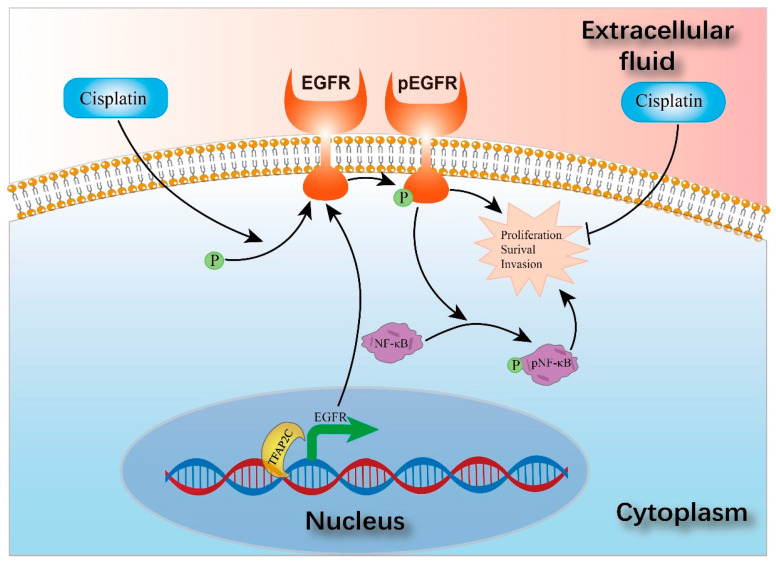
The ability of BCa cells to proliferate, survive, invade and migrate is impaired by cisplatin. However, the cells also activate cellular protective mechanisms by activating EGFR, which leads to depletion of EGFR; knockdown of TFAP2C further reduces EGFR, leading to a further decrease in its activation. In conclusion, TFAP2C is an important factor in maintaining EGFR levels in cisplatin-induced EGFR activation.

The mechanism diagram is shown in Figure 10.

## 5. Conclusions

We showed that TFAP2C knockdown enhanced the anti-tumor effects of cisplatin in vivo and in vitro by reducing the levels of EGFR and NF-κB activation, thereby providing a new idea for improving the efficacy of cisplatin.

## Figures and Tables

**Table 1:** Antibodies used in WB.

Antibodies	Company	Catalog Number	Host	Dilution
GAPDH	Servicebio	GB15004	Rabbit	1:1000
EGFR	Zhengneng Biotechnology	R22778	Mouse	1:1000
pEGFR	Cell Signaling Technology	#3777T	Rabbit	1:1000
NF-κB	Proteintech	10745-1-AP	Rabbit	1:1000
pNF-κB	Cell Signaling Technology	#3033	Rabbit	1:1000
Bax	Proteintech	60267-1-Ig	Mouse	1:5000
Bcl-2	Cell Signaling Technology	#15071	Mouse	1:1000
TFAP2C	Proteintech	14572-1-AP	Rabbit	1:1000
Caspase-3	Proteintech	19677-1-AP	Rabbit	1:1000
E-cadherin	Proteintech	20874-1-AP	Rabbit	1:5000
N-cadherin	Proteintech	22018-1-AP	Rabbit	1:5000
Cyclin D1	Proteintech	60186-1-Ig	Mouse	1:5000
GAPDH	Servicebio	GB15004	Rabbit	1:1000

**Table 2:** Antibodies used in IHC.

Antibodies	Company	Catalog Number	Host	Dilution
TFAP2C	Proteintech	14572-1-AP	Rabbit	1:400
Bax	Cell Signaling Technology	60267-1-Ig	Mouse	1:1000
Vimentin	Proteintech	10366-1-AP	Rabbit	1:5000

**Table 3:** Antibodies used in IF.

Antibodies	Company	Catalog Number	Host	Dilution
pEGFR	Cell Signaling Technology	#3777T	Rabbit	1:800
pNF-κB	Cell Signaling Technology	#3033	Rabbit	1:800
Bcl-2	Proteintech	60178-1-Ig	Mouse	1:400
Ki67	Proteintech	27309-1-AP	Rabbit	1:200
Vimentin	Proteintech	10366-1-AP	Rabbit	1:50

**Table 4:** Clinicopathological characteristics of 22 patients with BCa.

Variable	Groups	Total
Sex	Male	16
	Female	6
Age (years)	≥60	14
	<60	8
Tumor size (cm)	<3	7
	≥3	15
Multiplicity of tumor	Single	10
	Multiple	12
Tumor grade	Low grade	6
	High grade	16
Tumor stage	Ta, T1	4
	T2–T4	18
Lymph nodes	Negative	17
	Positive	5
Distant metastasis	Absent	22
	Present	0

## Data Availability

The raw data of this article will be made available by the authors, without undue reservation.

## Supplementary Materials

The following supporting information can be downloaded at: https://www.mdpi.com/article/10.3390/cancers14194809/s1, Figure S1: Quantitative analysis of protein expression levels.; Figure S2: Quantitative analysis of protein expression levels. File S1: Original blots.
