# Supplementary material for: TFAP2C Knockdown Sensitizes Bladder Cancer Cells to Cisplatin Treatment via Regulation of EGFR and NF-κB
Source: Cancers (Basel). 2022 Sep 30;14(19):4809. doi: 10.3390/cancers14194809 (PMC9562889; doi:10.3390/cancers14194809)
Supplement: Supplementary file 1 [file cancers-14-04809-s001.zip › cancers-1883913-Supplementary Figure S1,S2.pdf]

Supplementary Materials: The following supporting information can be downloaded at: www.mdpi.com/xxx/s1, Figure S1-2

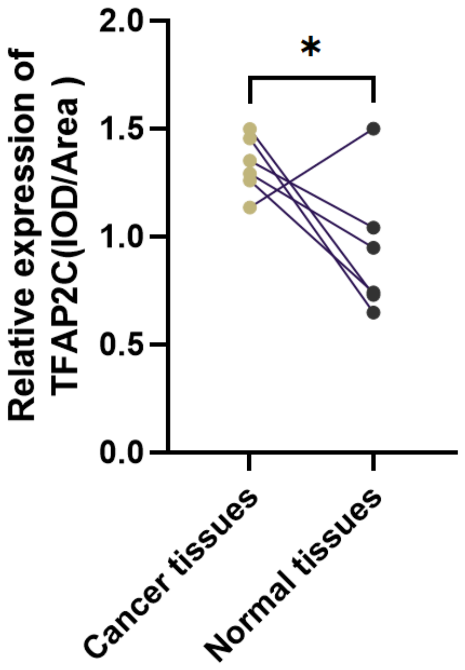

Figure S1: Quantitative analysis of protein expression levels. Six paired BCa tissues and adjacent tissues were used for immunohistochemistry. (*P < 0.05, **P < 0.01, ***P < 0.001, ns, not significant.)

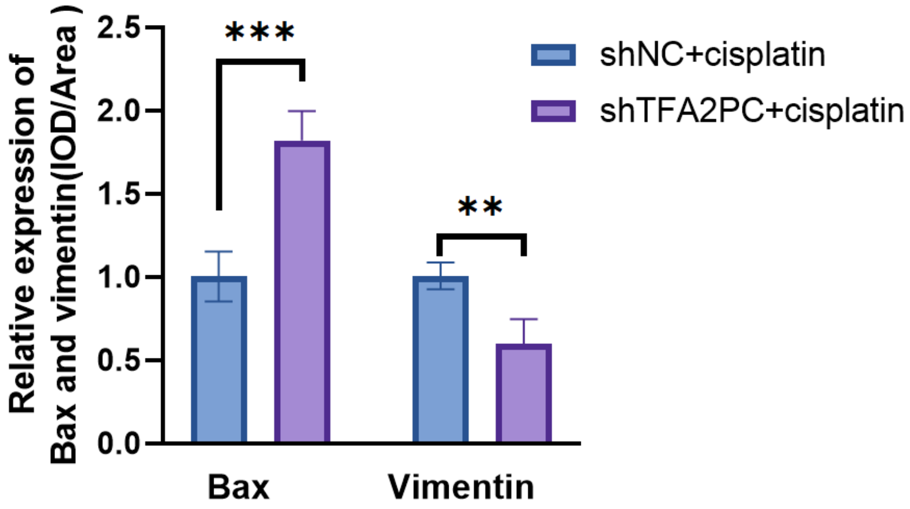

Figure S2: Quantitative analysis of protein expression levels. shTFAP2C +cisplatin group had a higher level of Bax and lower level of vimentin than shNC+cisplatin group. （*P < 0.05, **P < 0.01, ***P < 0.001, ns, not significant. ）
