# Supplementary figures and images for: TFAP2C Knockdown Sensitizes Bladder Cancer Cells to Cisplatin Treatment via Regulation of EGFR and NF-κB
Source: Cancers (Basel). 2022 Sep 30;14(19):4809. doi: 10.3390/cancers14194809 (PMC9562889; doi:10.3390/cancers14194809)

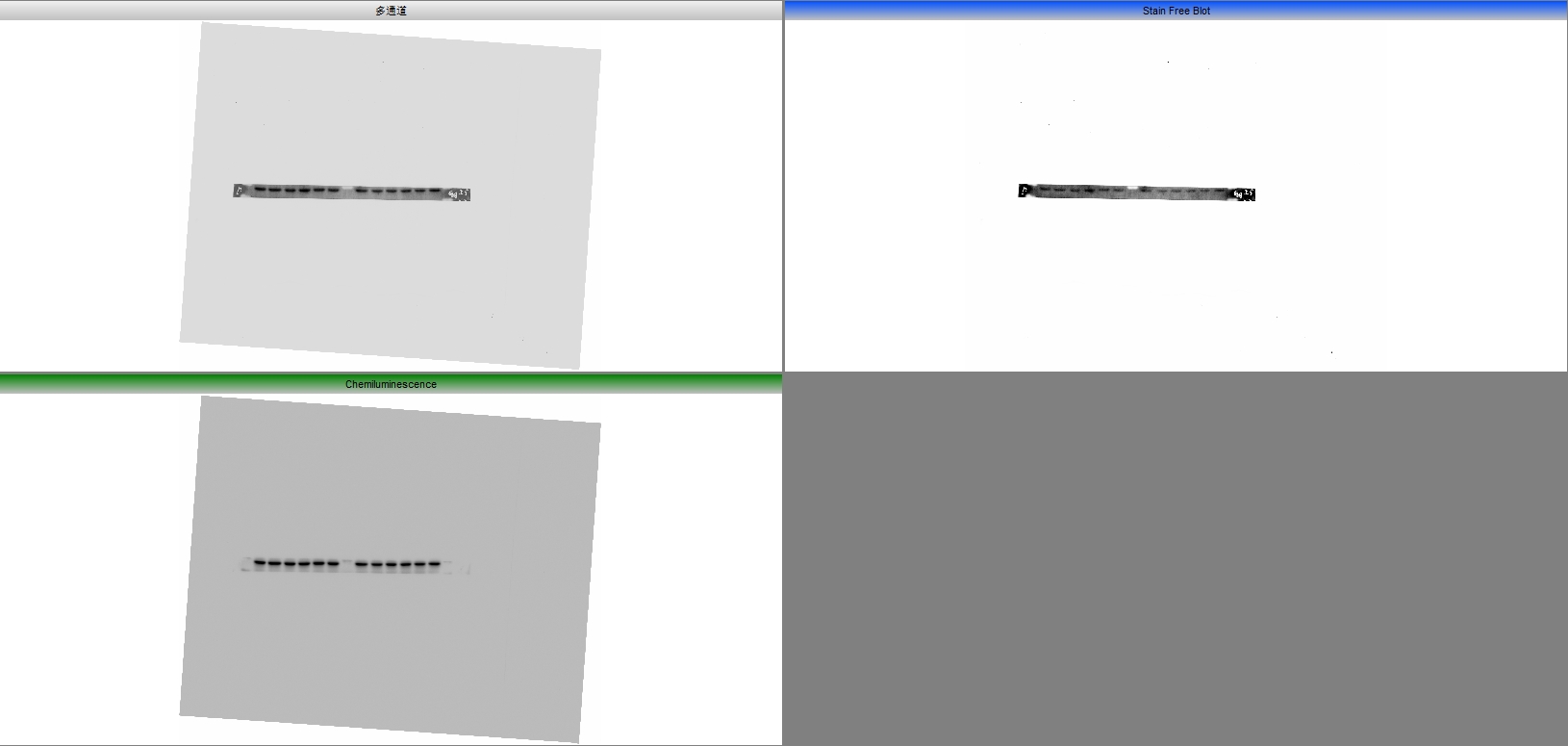

Supplement: Supplementary file 1 [file cancers-14-04809-s001.zip › cancers-1883913-Supplementary File S1. original whole blot/fig2/5637-gapdh.jpg]

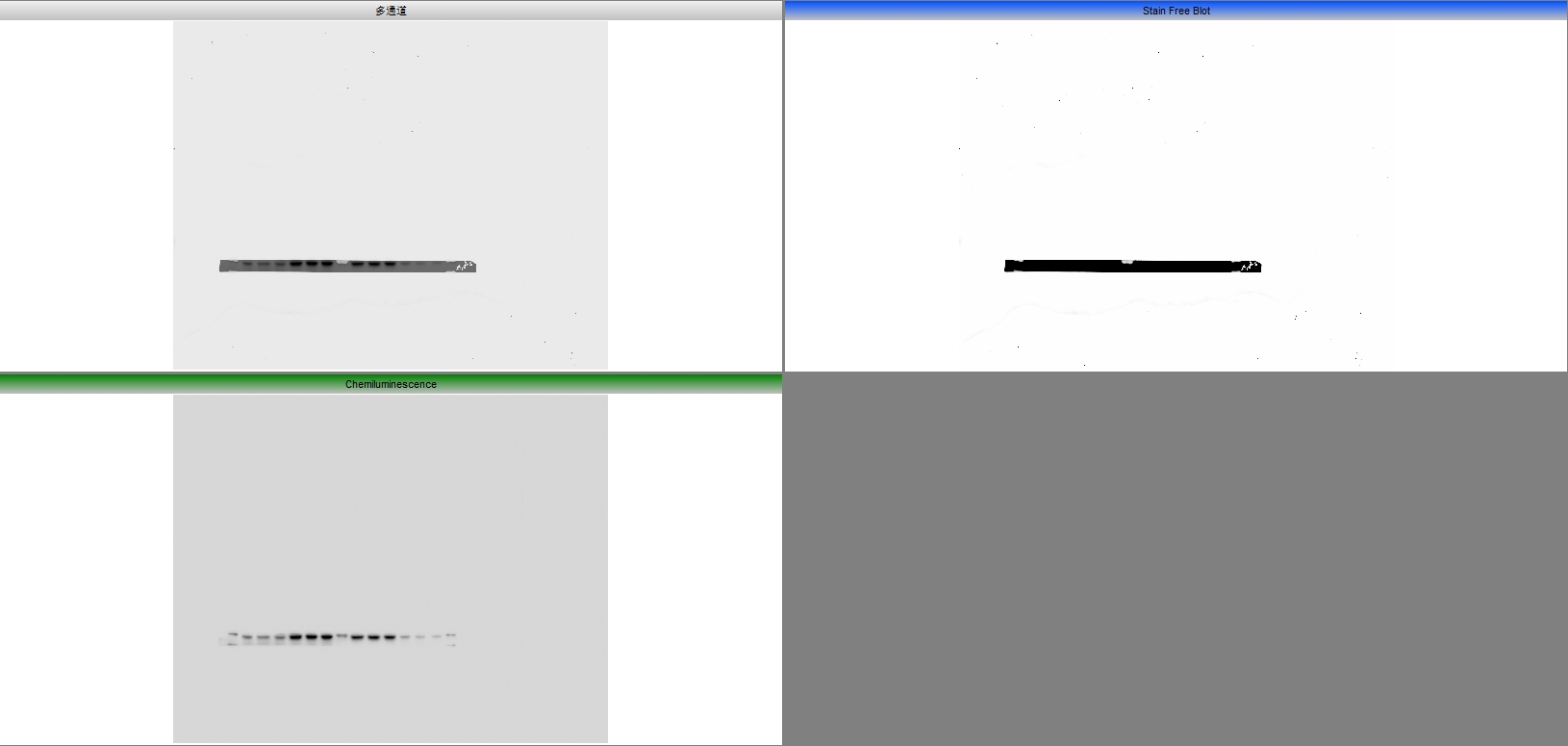

Supplement: Supplementary file 1 [file cancers-14-04809-s001.zip › cancers-1883913-Supplementary File S1. original whole blot/fig2/5737-TFAP2C.jpg]

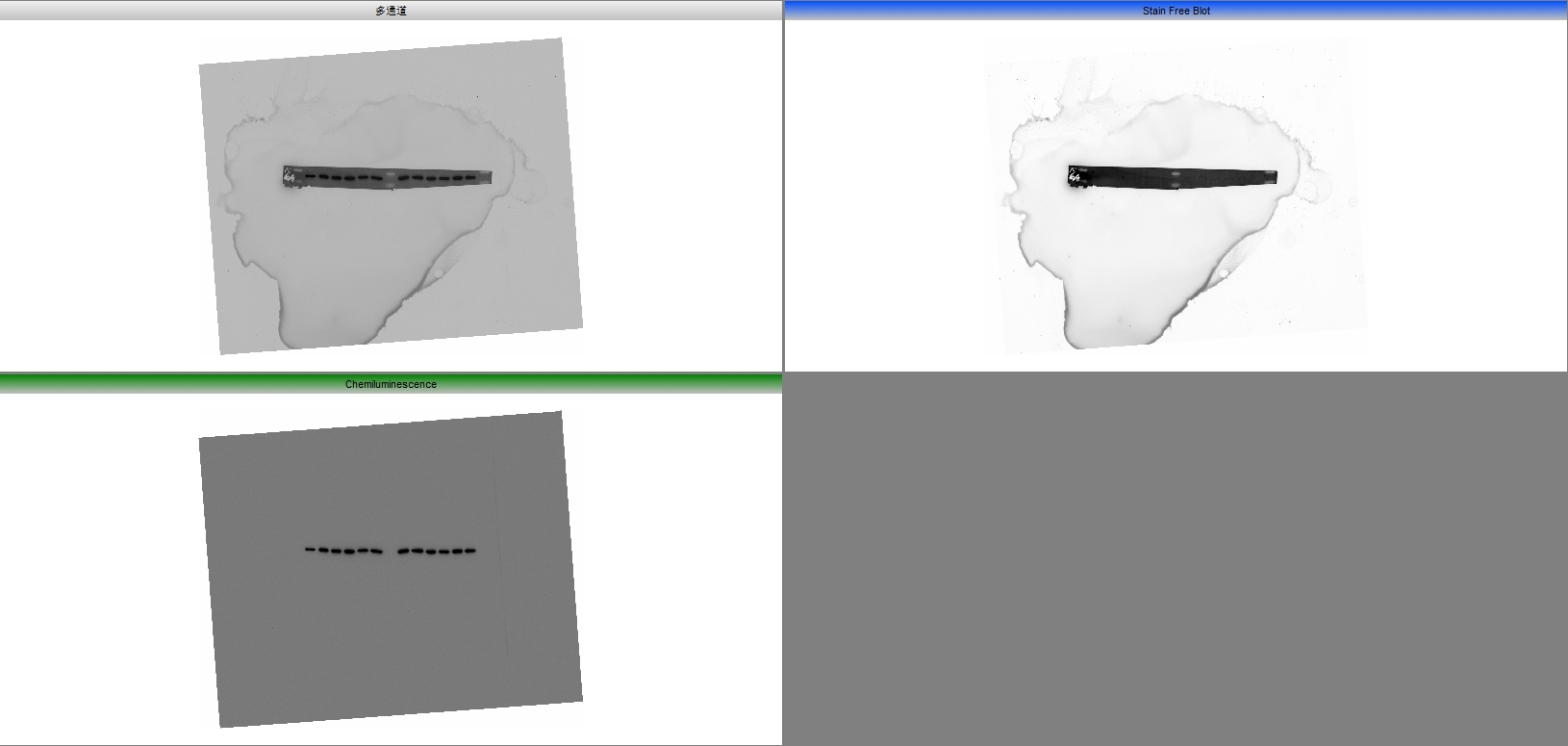

Supplement: Supplementary file 1 [file cancers-14-04809-s001.zip › cancers-1883913-Supplementary File S1. original whole blot/fig2/T24-gapdh.jpg]

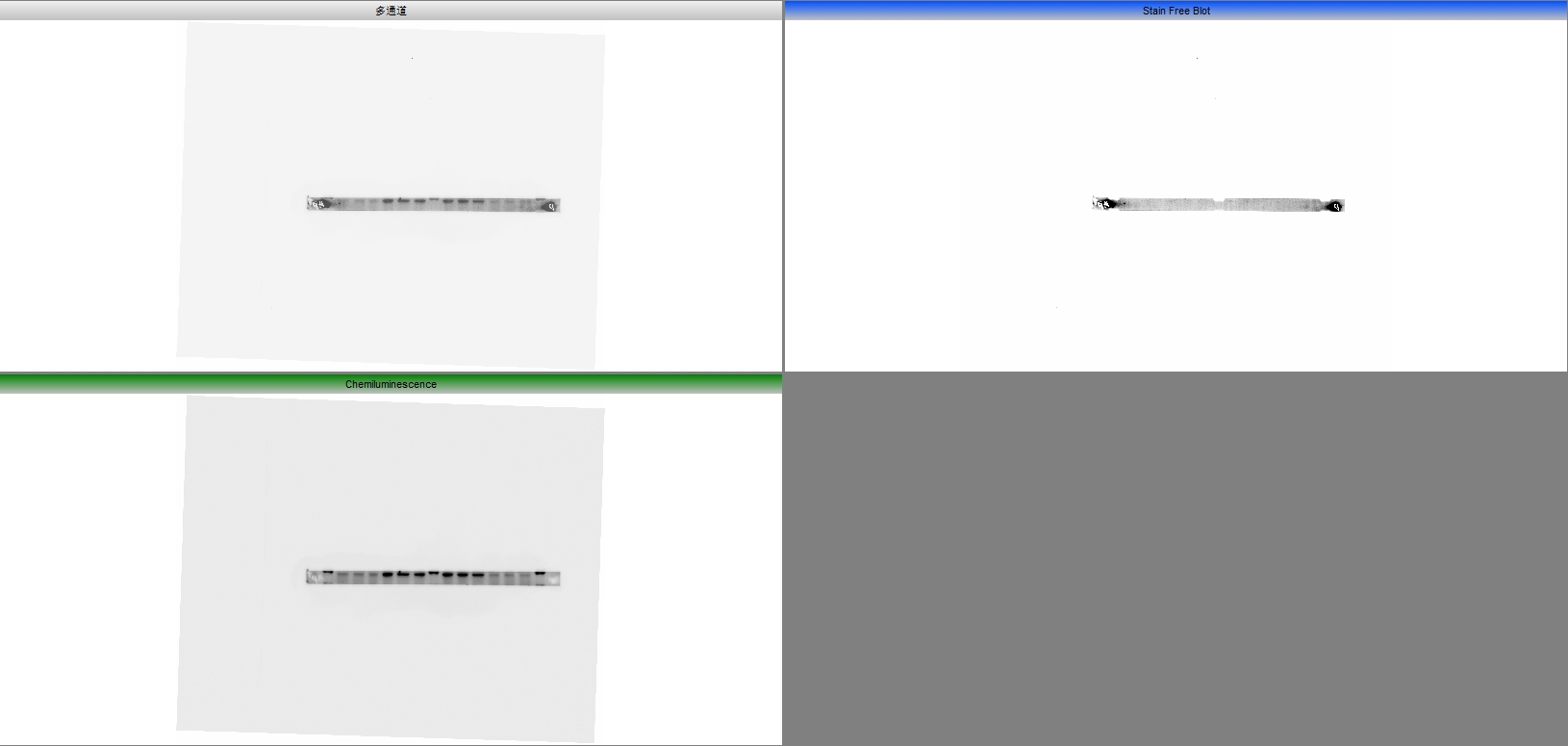

Supplement: Supplementary file 1 [file cancers-14-04809-s001.zip › cancers-1883913-Supplementary File S1. original whole blot/fig2/T24-TFAP2C.jpg]

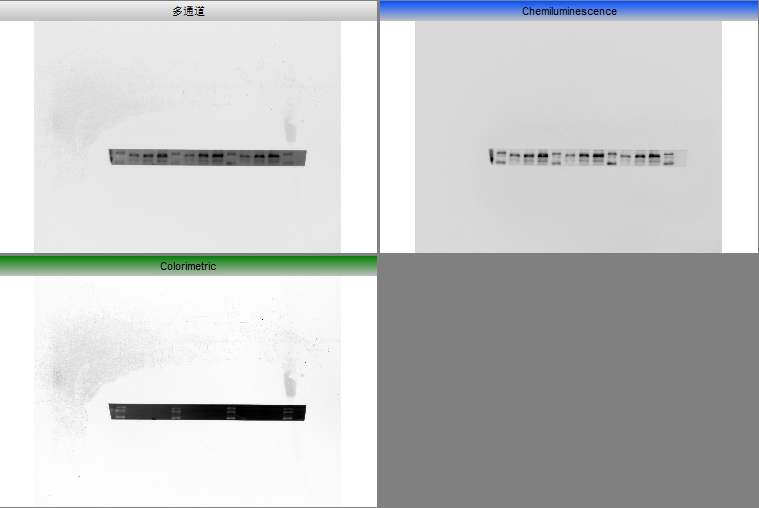

Supplement: Supplementary file 1 [file cancers-14-04809-s001.zip › cancers-1883913-Supplementary File S1. original whole blot/fig3/5637 E-cadhern.jpg]

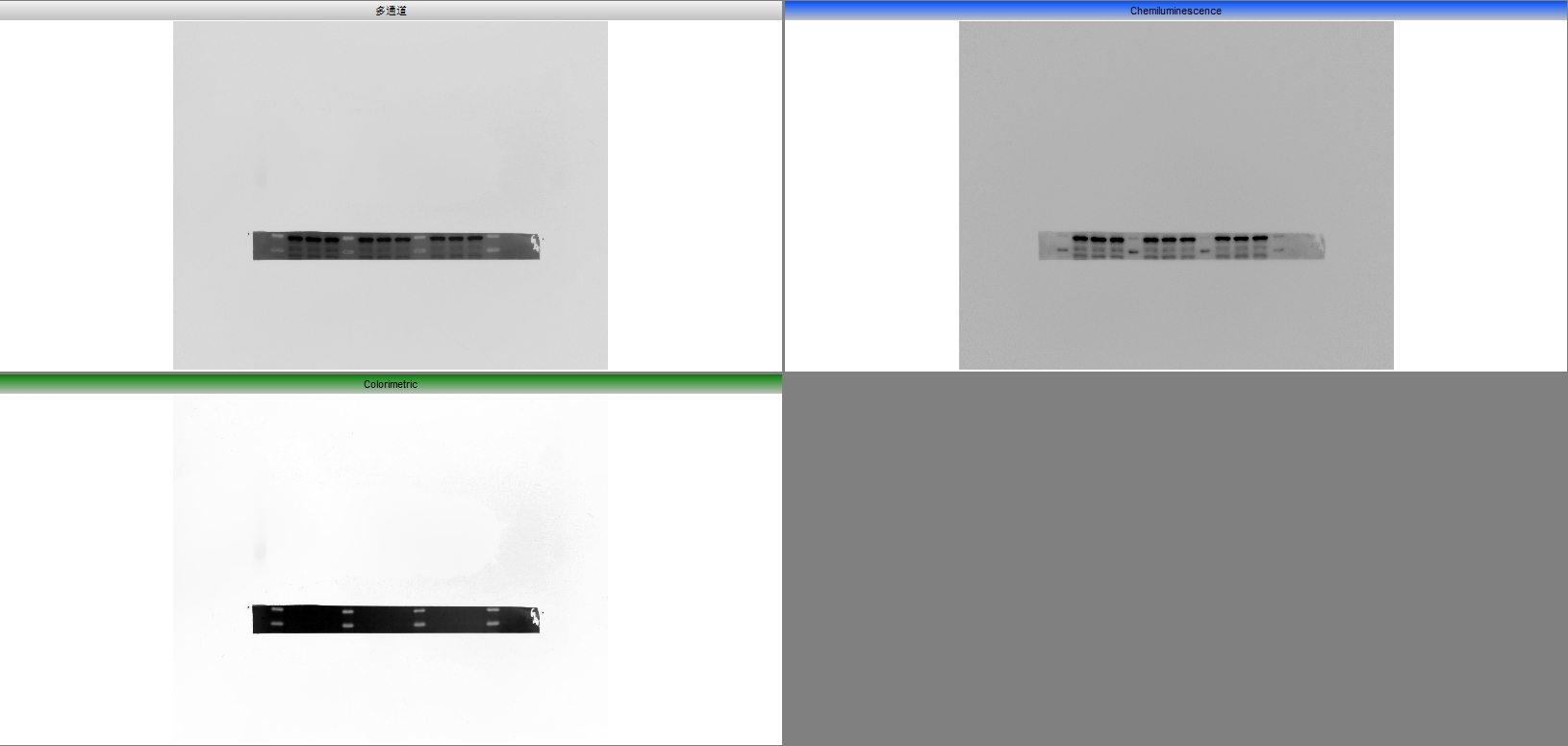

Supplement: Supplementary file 1 [file cancers-14-04809-s001.zip › cancers-1883913-Supplementary File S1. original whole blot/fig3/5637 GAPDH.jpg]

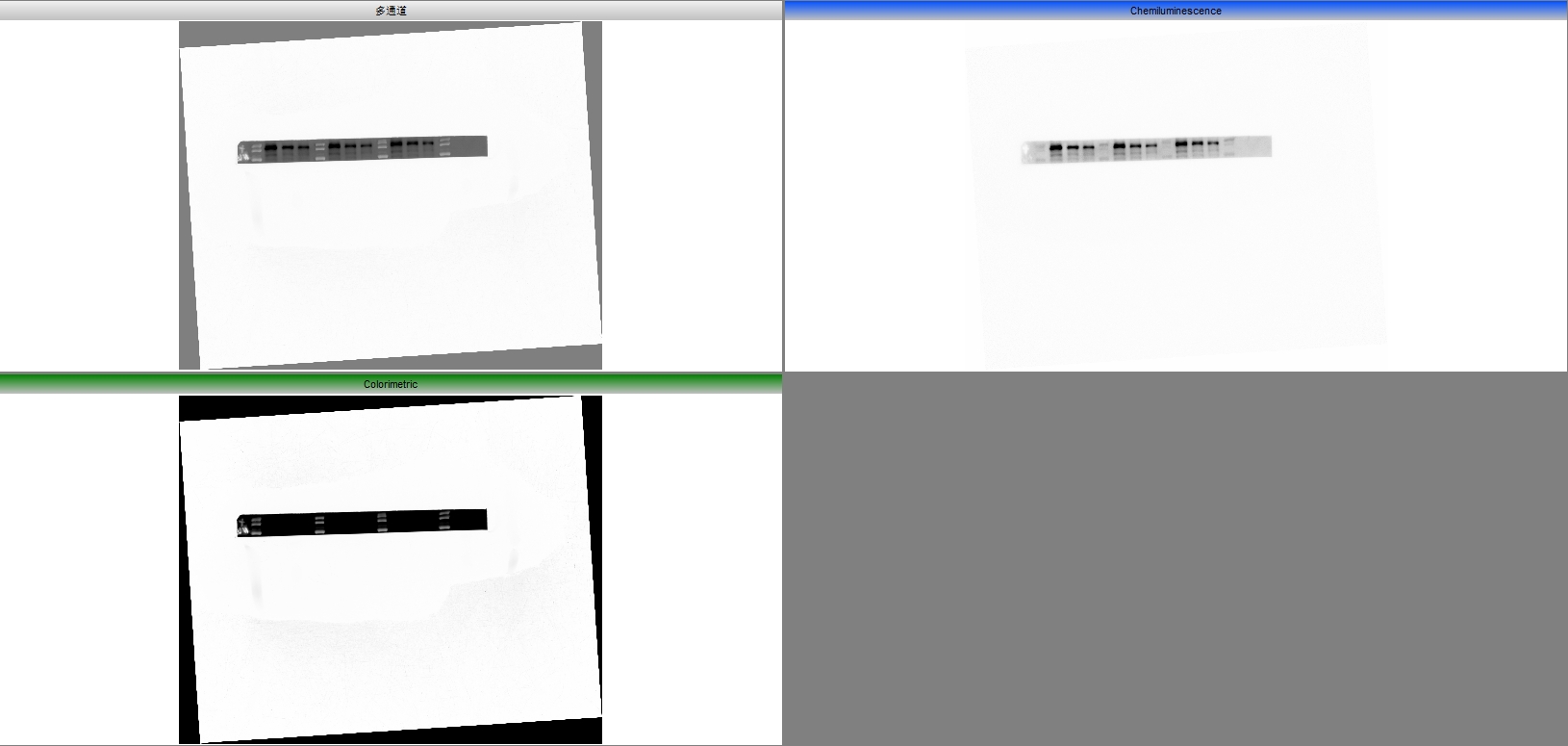

Supplement: Supplementary file 1 [file cancers-14-04809-s001.zip › cancers-1883913-Supplementary File S1. original whole blot/fig3/5637 N-cadhern.jpg]

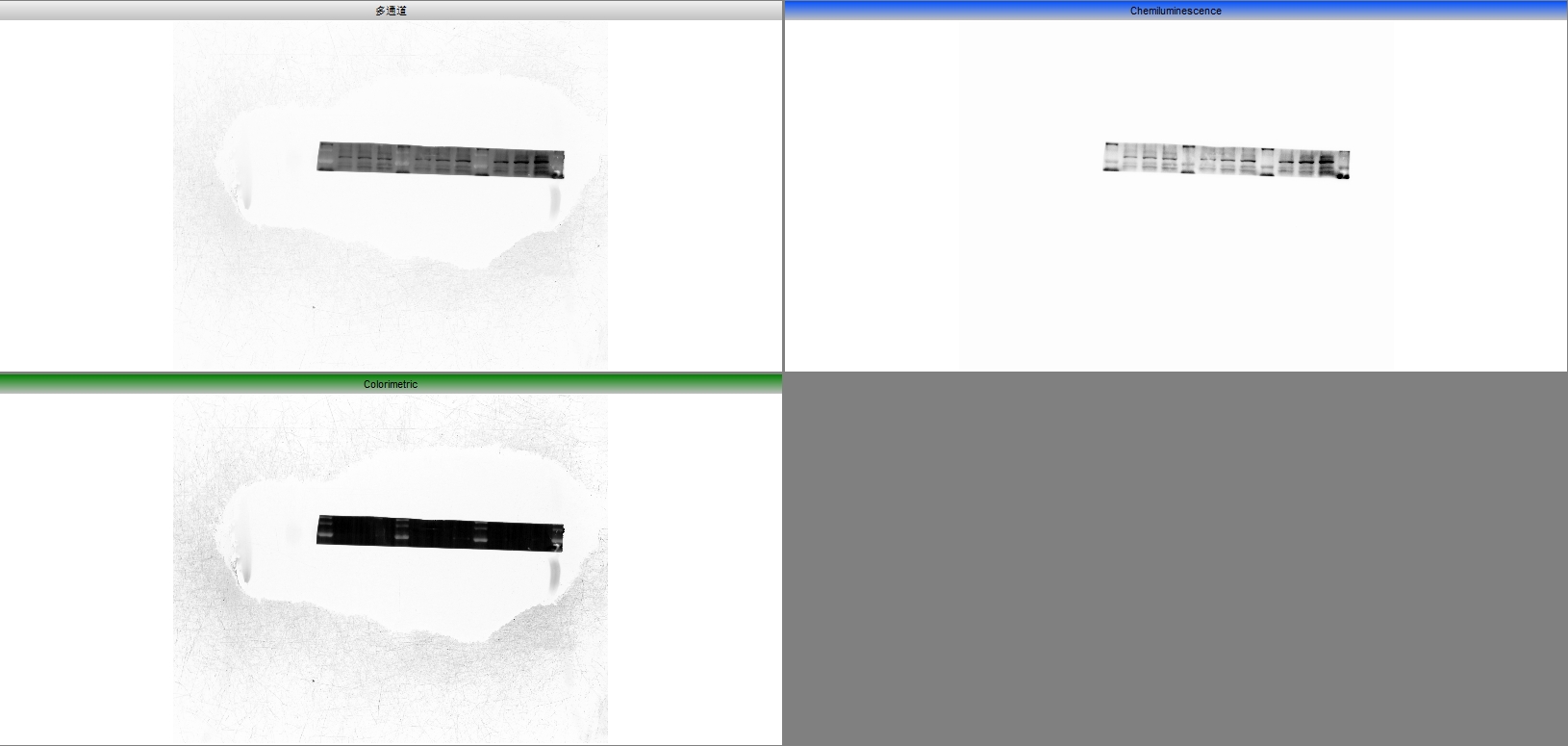

Supplement: Supplementary file 1 [file cancers-14-04809-s001.zip › cancers-1883913-Supplementary File S1. original whole blot/fig3/T24 E-cadhern.jpg]

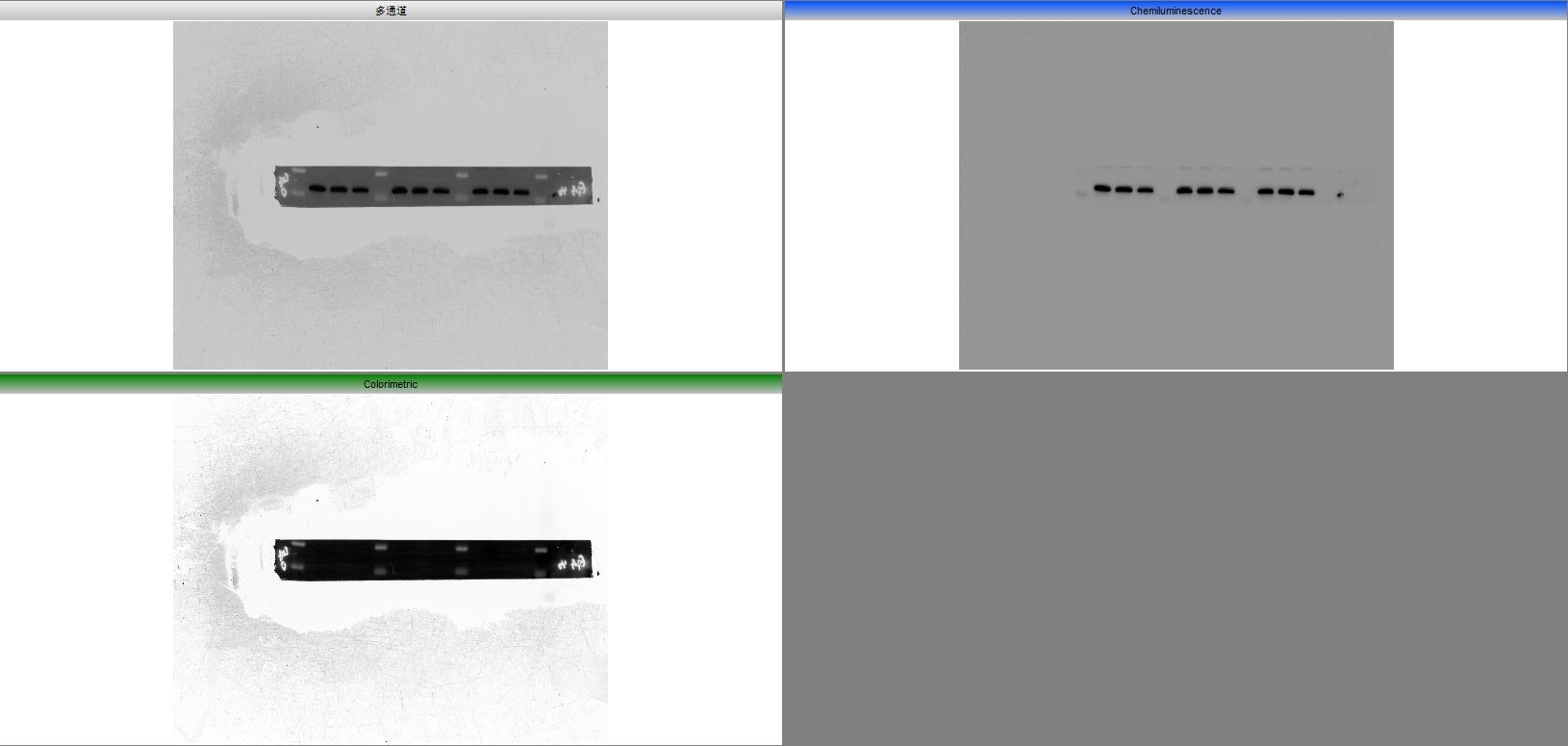

Supplement: Supplementary file 1 [file cancers-14-04809-s001.zip › cancers-1883913-Supplementary File S1. original whole blot/fig3/T24 GAPDH.jpg]

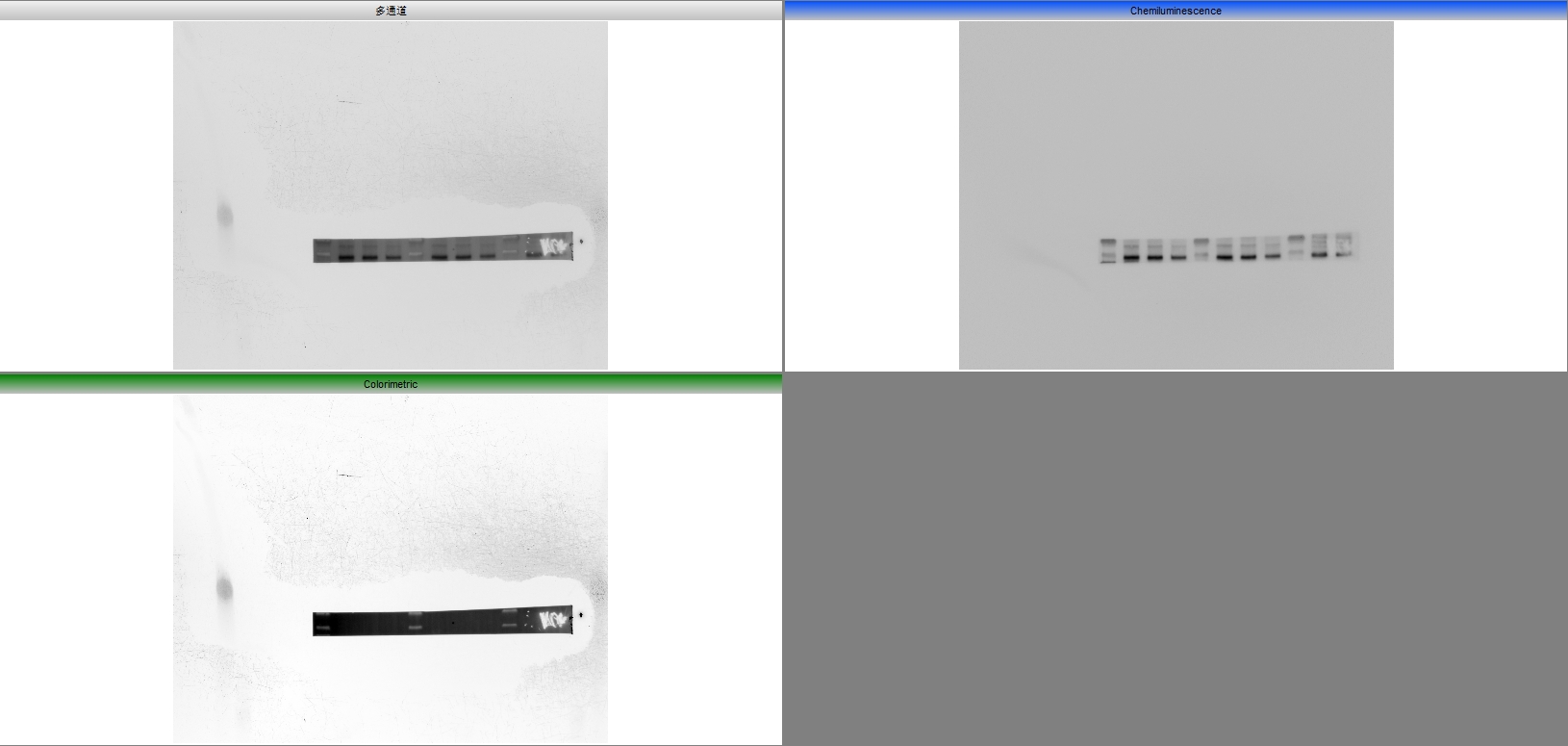

Supplement: Supplementary file 1 [file cancers-14-04809-s001.zip › cancers-1883913-Supplementary File S1. original whole blot/fig3/T24 N-cadhern1.jpg]

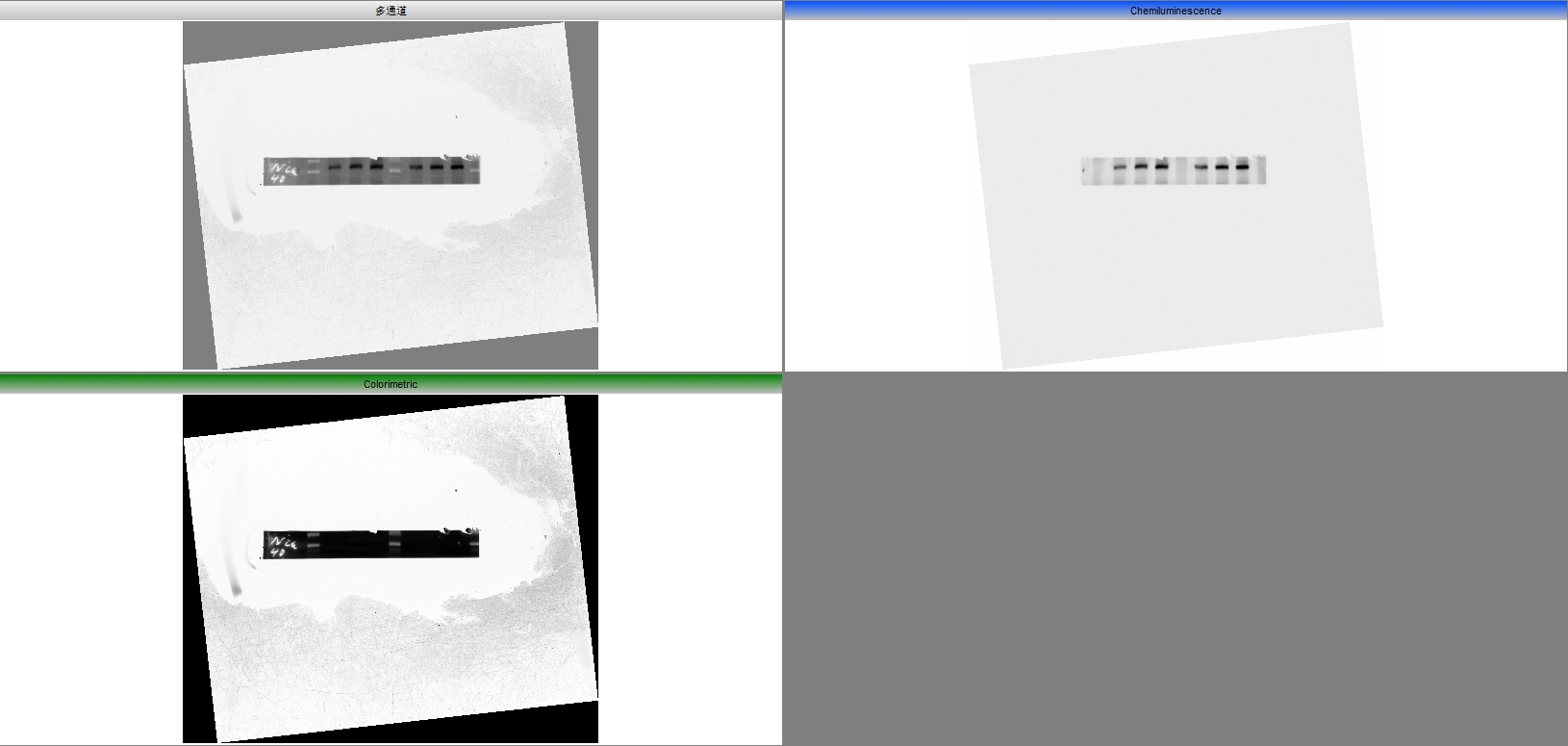

Supplement: Supplementary file 1 [file cancers-14-04809-s001.zip › cancers-1883913-Supplementary File S1. original whole blot/fig3/T24 N-cadhern2.jpg]

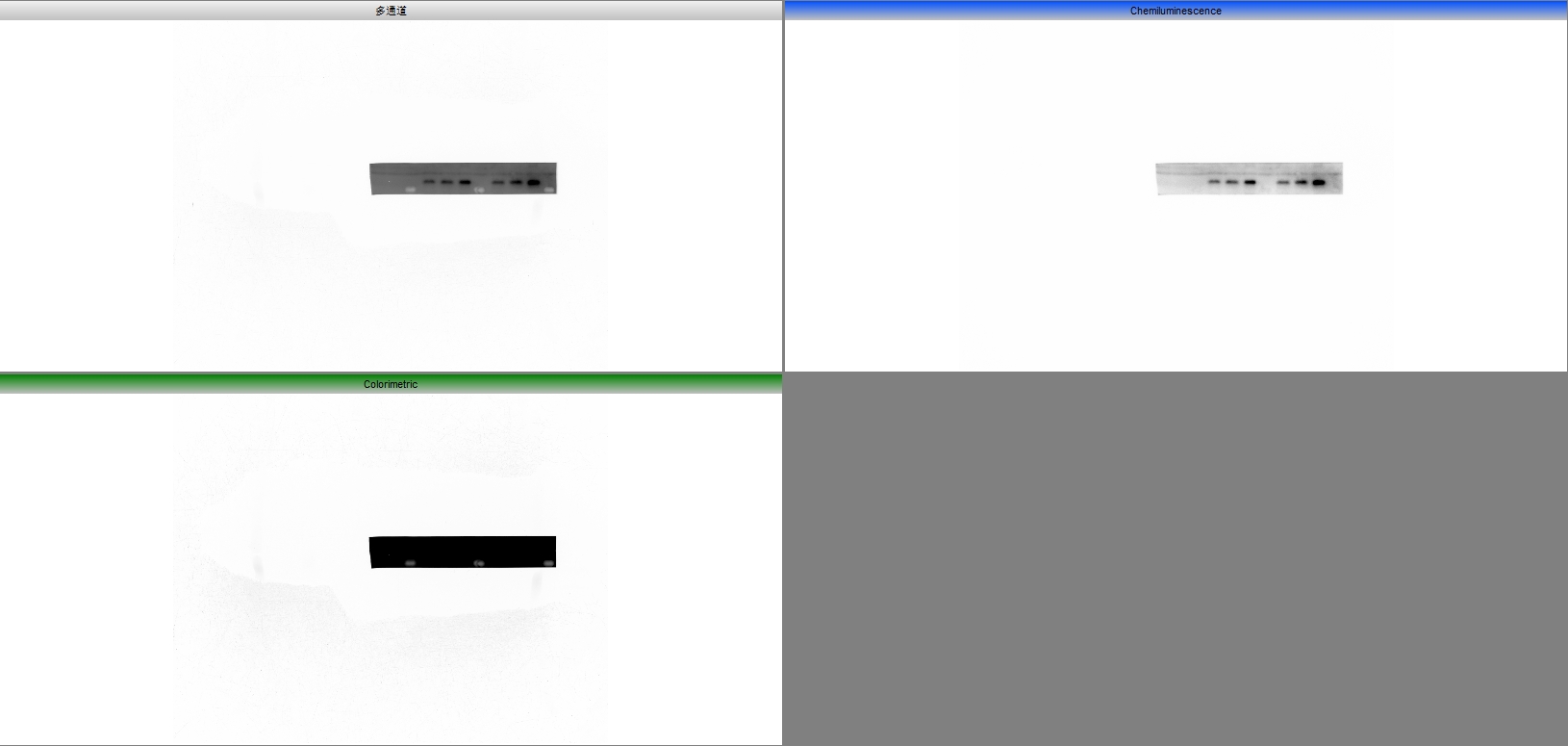

Supplement: Supplementary file 1 [file cancers-14-04809-s001.zip › cancers-1883913-Supplementary File S1. original whole blot/fig4/5637 Bax1.jpg]

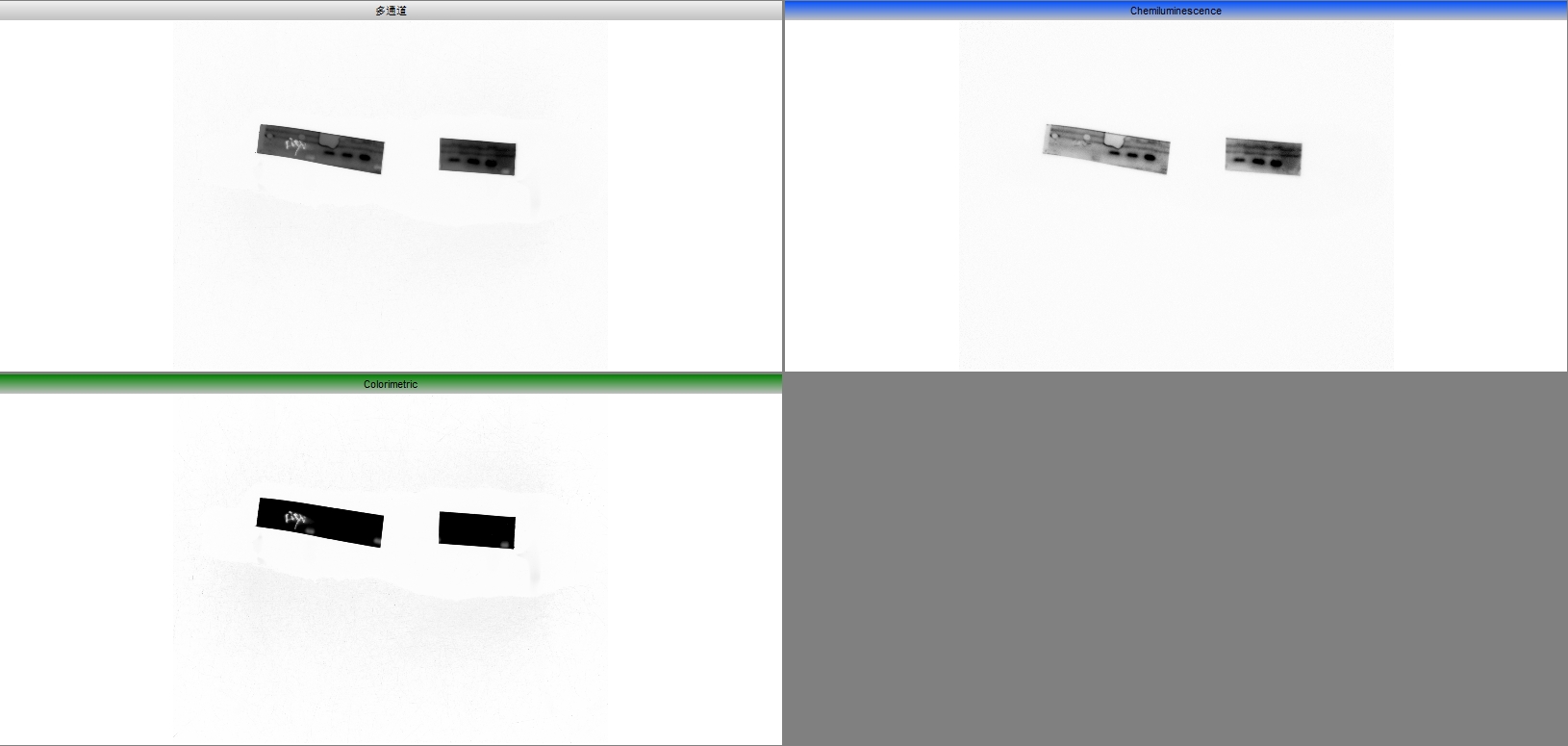

Supplement: Supplementary file 1 [file cancers-14-04809-s001.zip › cancers-1883913-Supplementary File S1. original whole blot/fig4/5637 Bax2.jpg]

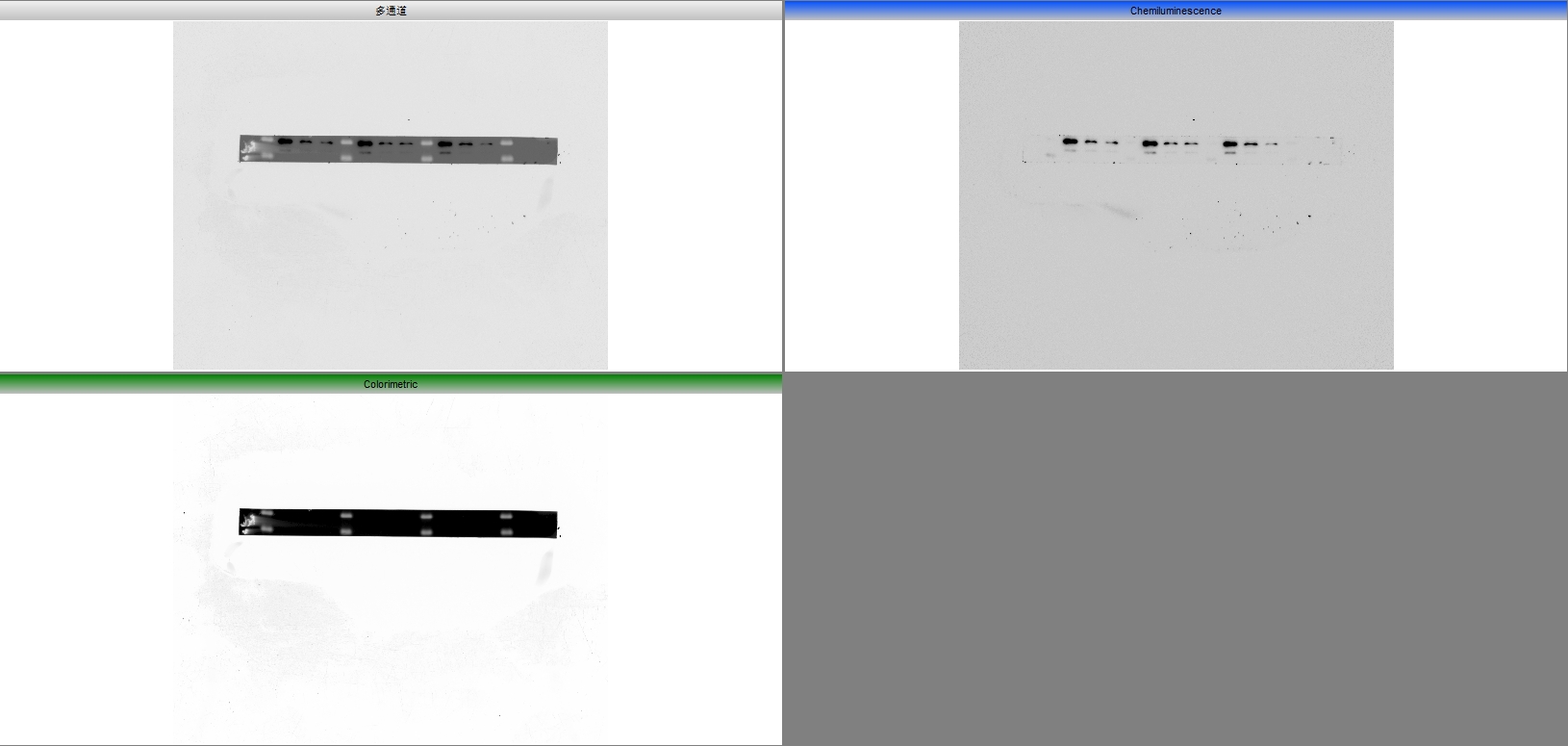

Supplement: Supplementary file 1 [file cancers-14-04809-s001.zip › cancers-1883913-Supplementary File S1. original whole blot/fig4/5637 Bcl-2.jpg]

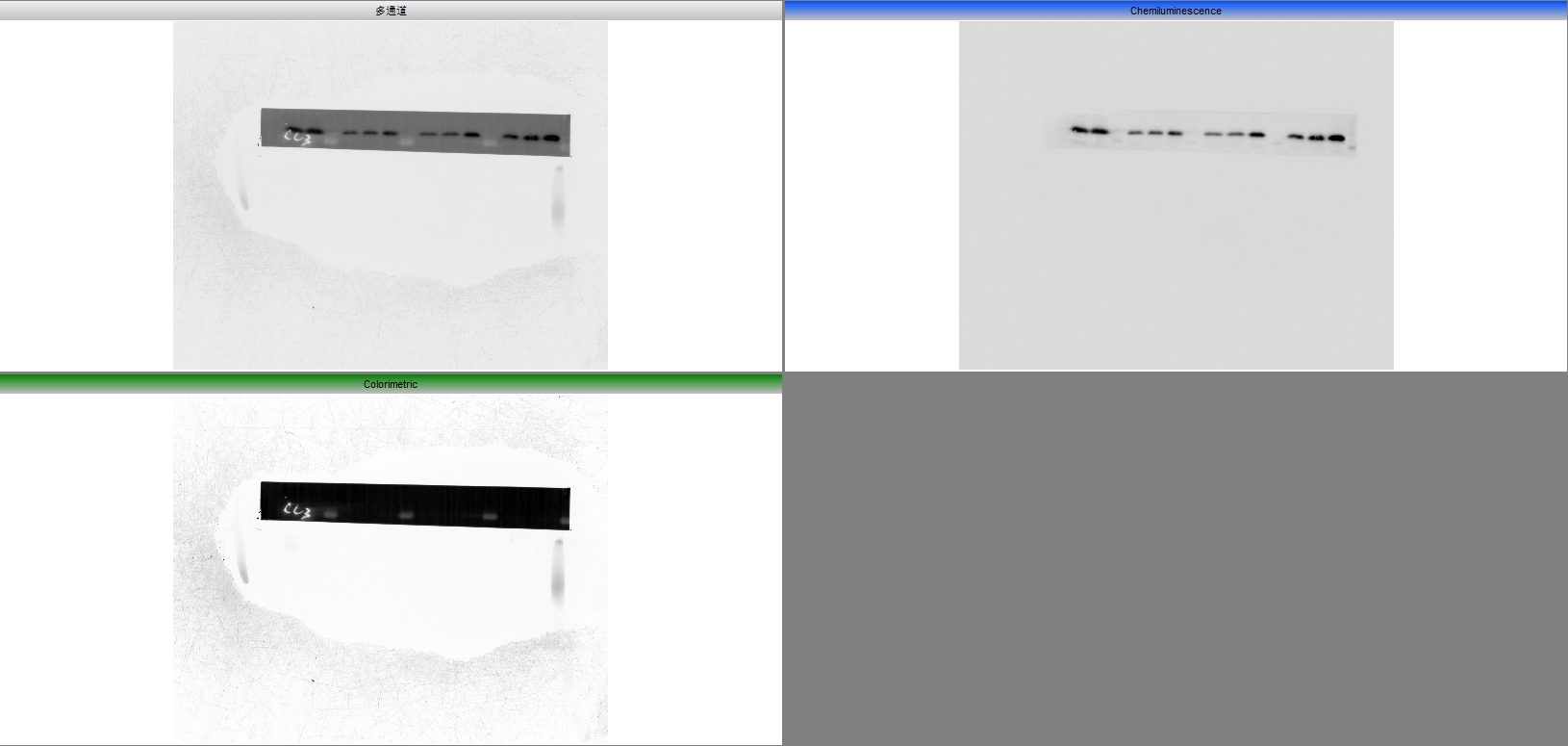

Supplement: Supplementary file 1 [file cancers-14-04809-s001.zip › cancers-1883913-Supplementary File S1. original whole blot/fig4/5637 Cleaved caspase-3.jpg]

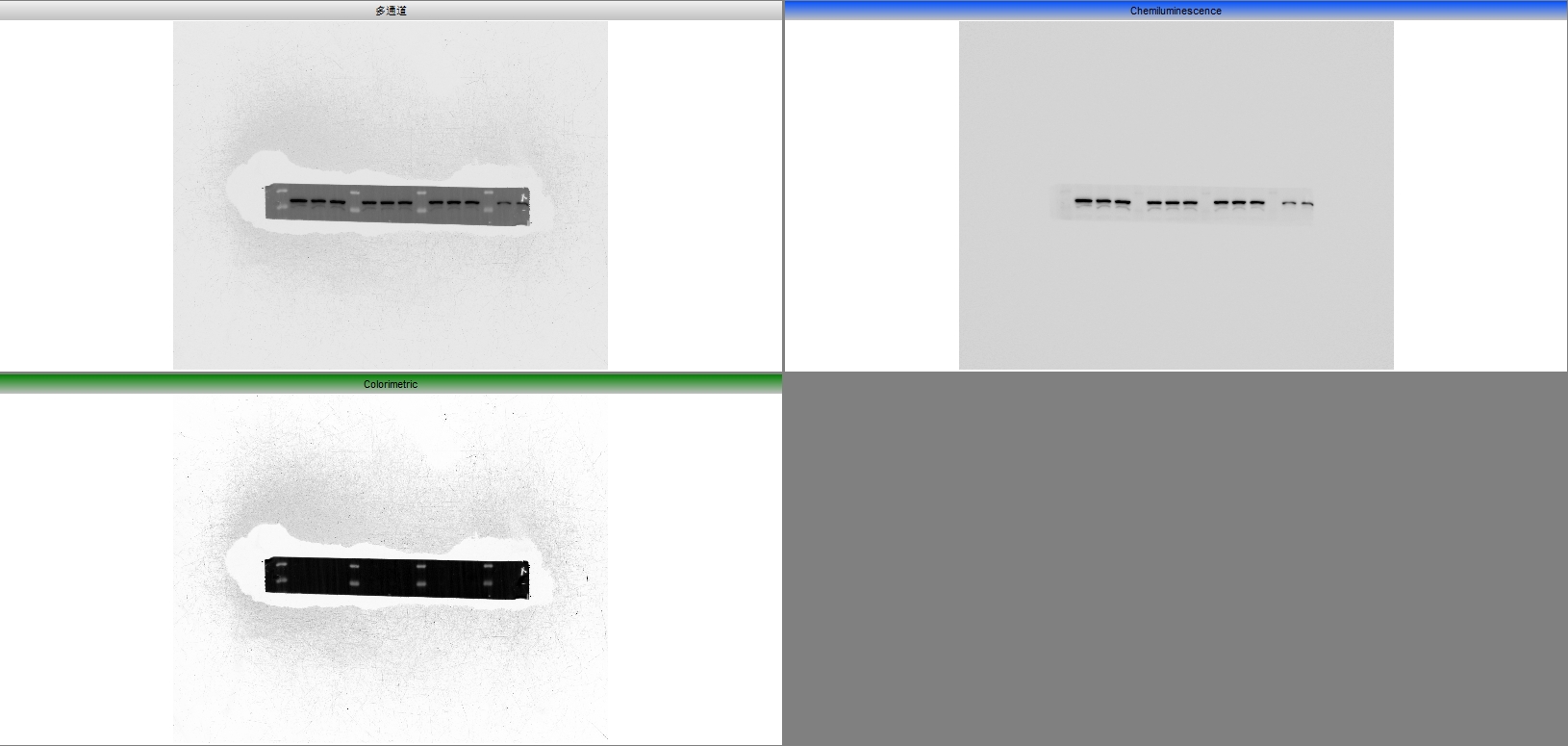

Supplement: Supplementary file 1 [file cancers-14-04809-s001.zip › cancers-1883913-Supplementary File S1. original whole blot/fig4/5637 GAPDH.jpg]

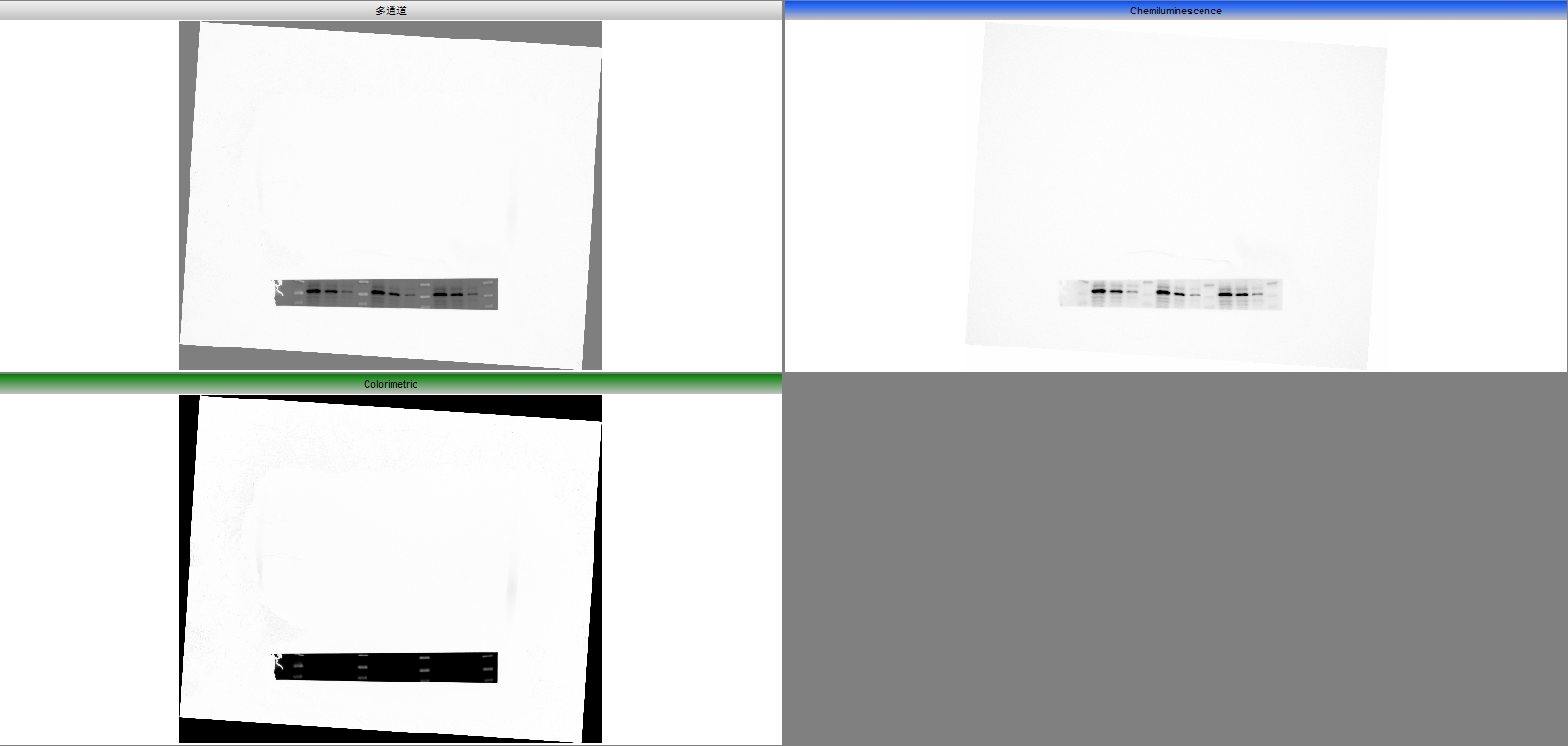

Supplement: Supplementary file 1 [file cancers-14-04809-s001.zip › cancers-1883913-Supplementary File S1. original whole blot/fig4/5637 Pro caspase-3.jpg]

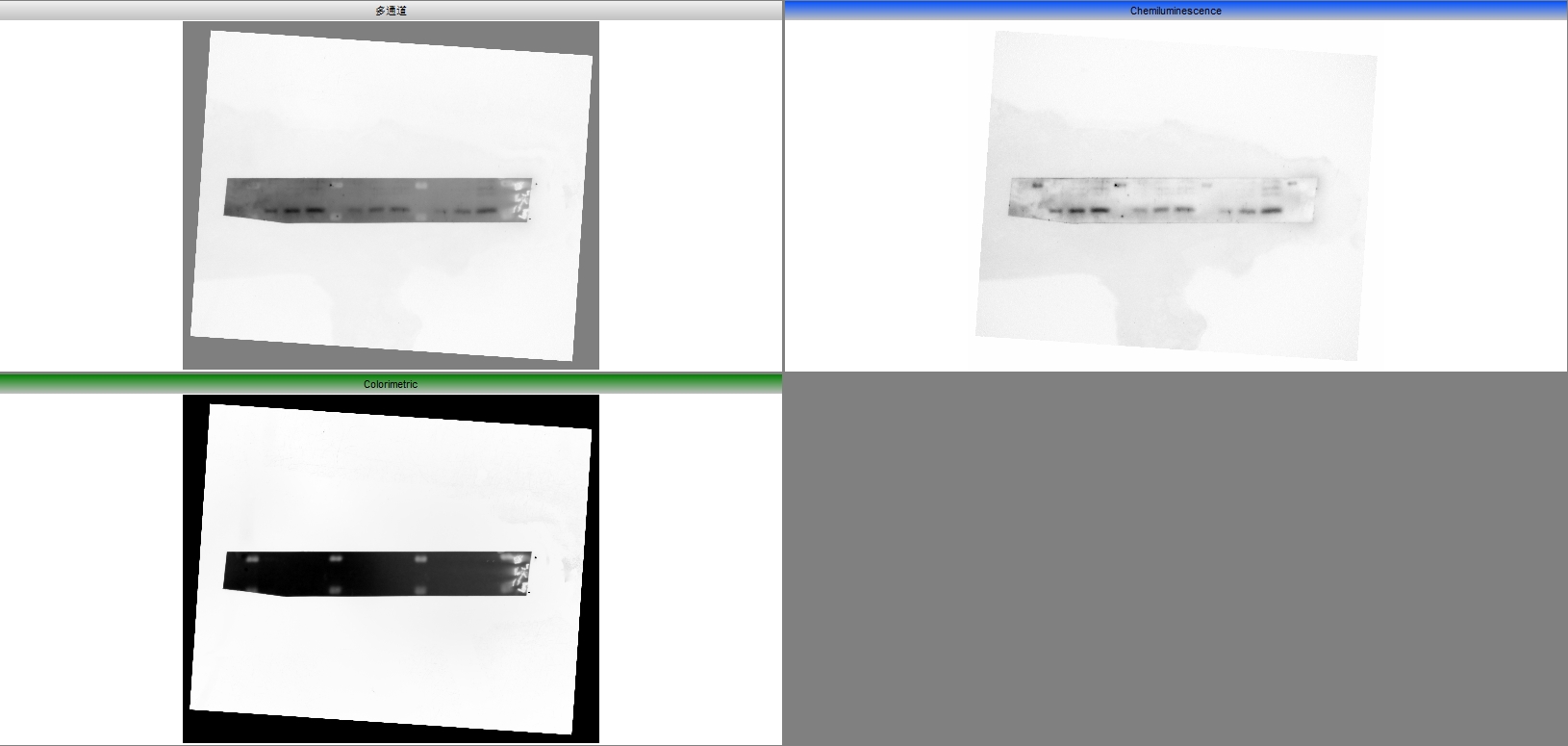

Supplement: Supplementary file 1 [file cancers-14-04809-s001.zip › cancers-1883913-Supplementary File S1. original whole blot/fig4/T24 Bax.jpg]

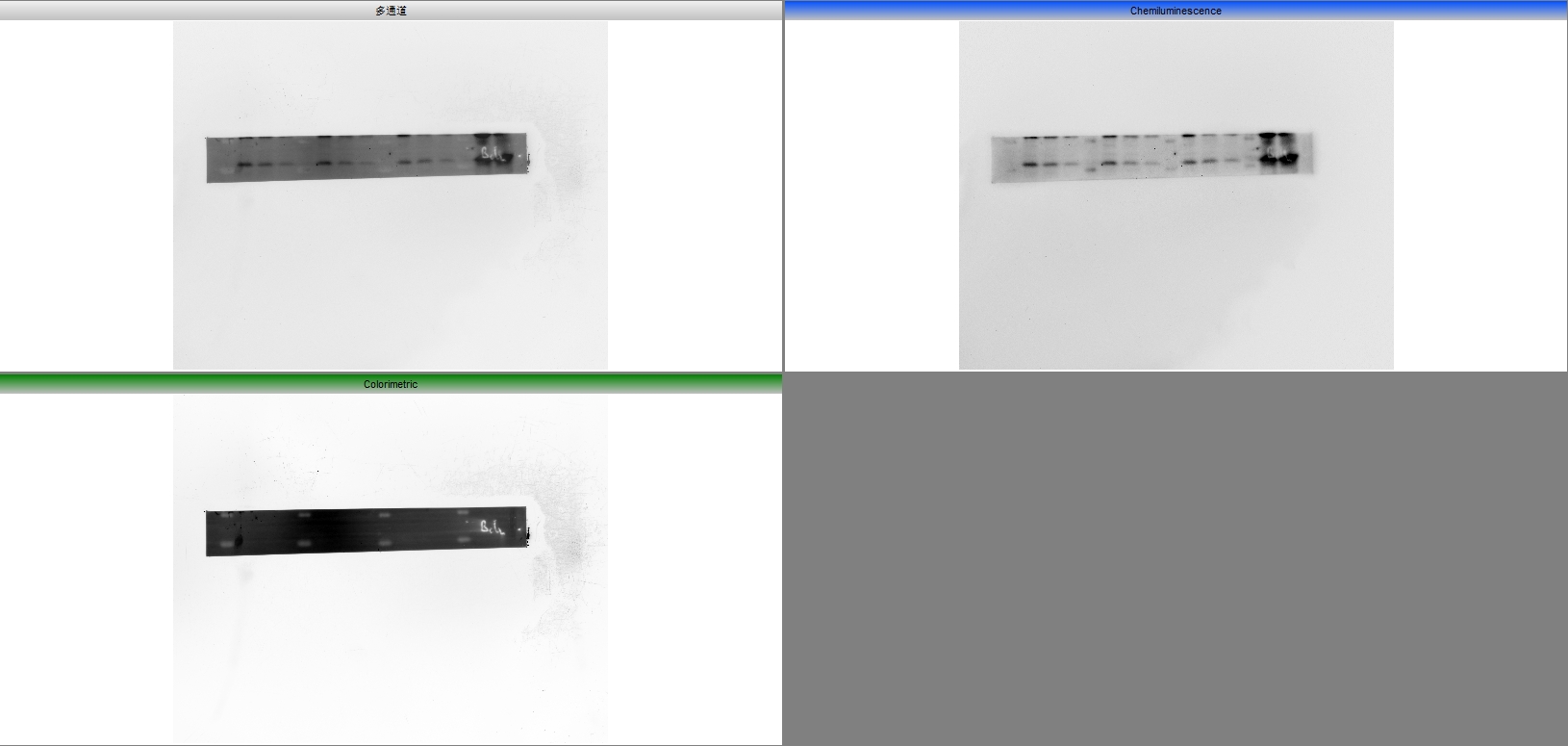

Supplement: Supplementary file 1 [file cancers-14-04809-s001.zip › cancers-1883913-Supplementary File S1. original whole blot/fig4/T24 Bcl-2.jpg]

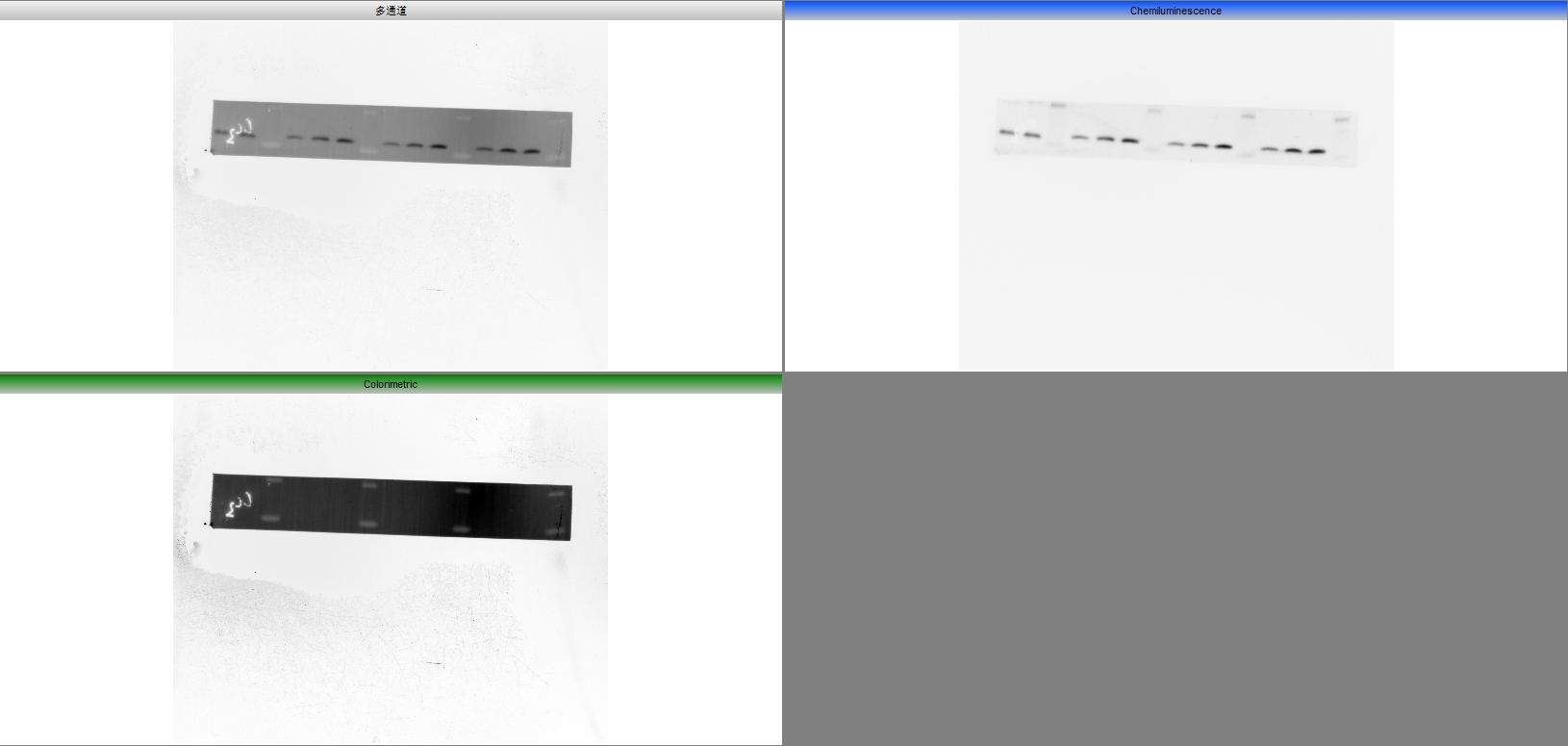

Supplement: Supplementary file 1 [file cancers-14-04809-s001.zip › cancers-1883913-Supplementary File S1. original whole blot/fig4/T24 Cleaved caspase-3.jpg]

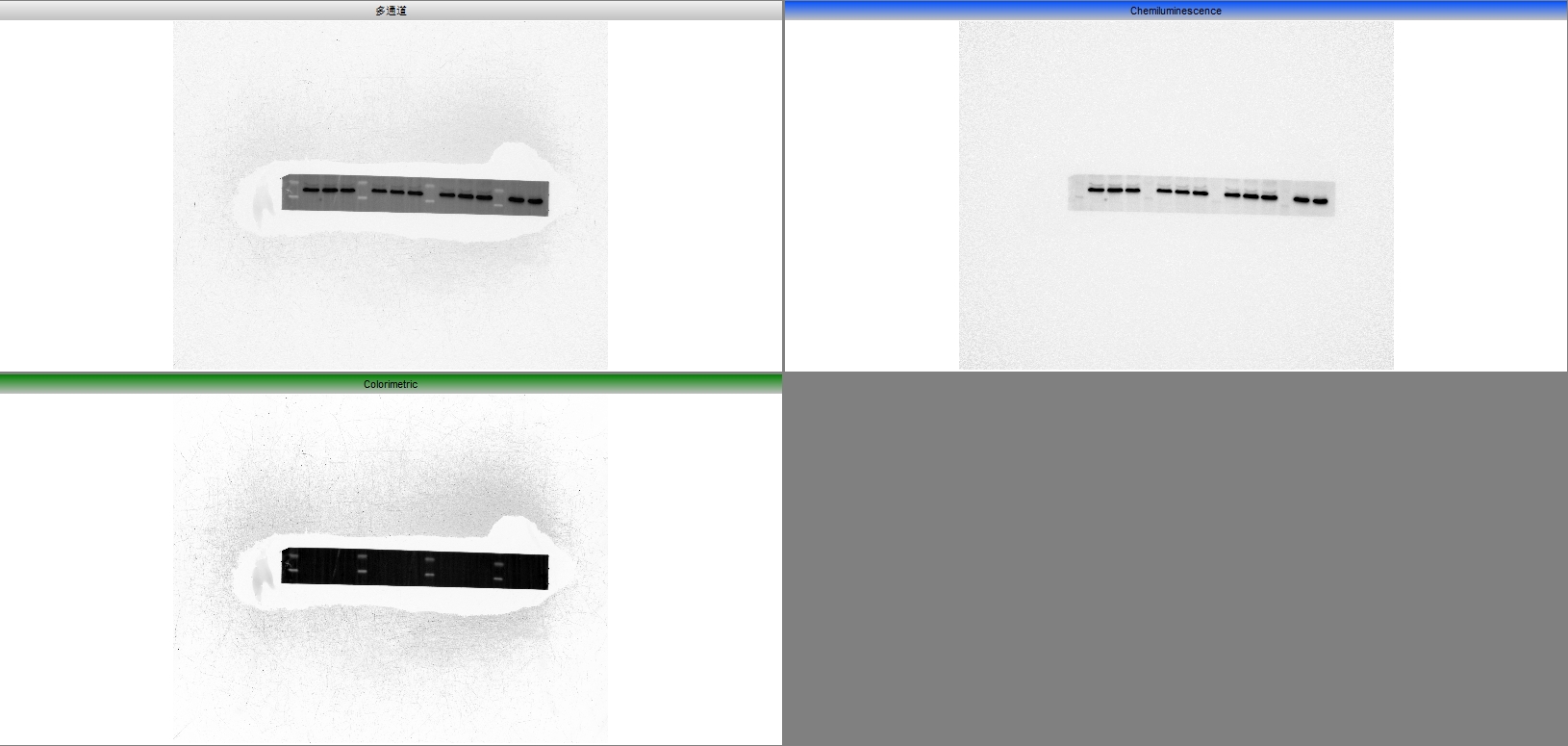

Supplement: Supplementary file 1 [file cancers-14-04809-s001.zip › cancers-1883913-Supplementary File S1. original whole blot/fig4/T24 GAPDH.jpg]

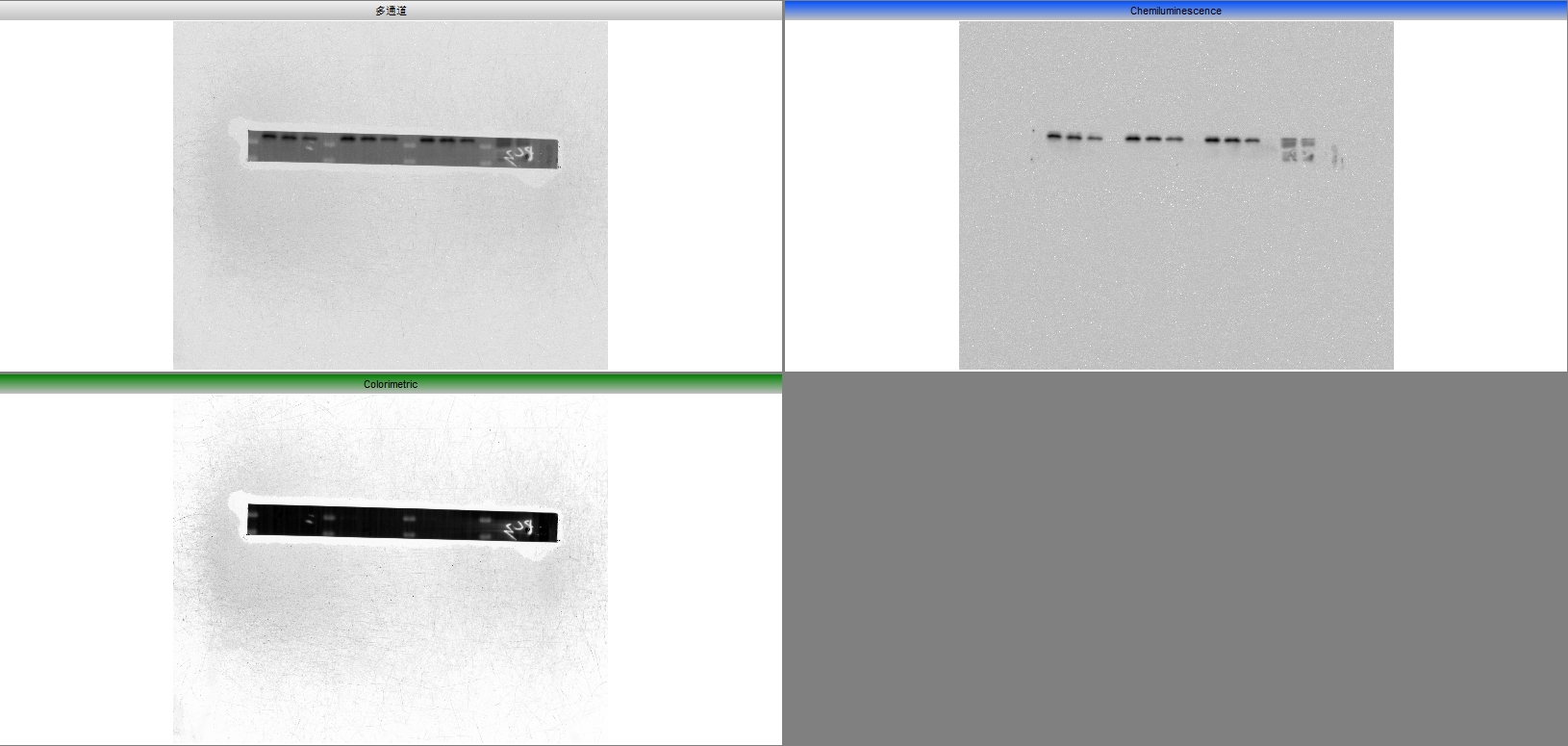

Supplement: Supplementary file 1 [file cancers-14-04809-s001.zip › cancers-1883913-Supplementary File S1. original whole blot/fig4/T24 Pro caspase-3.jpg]

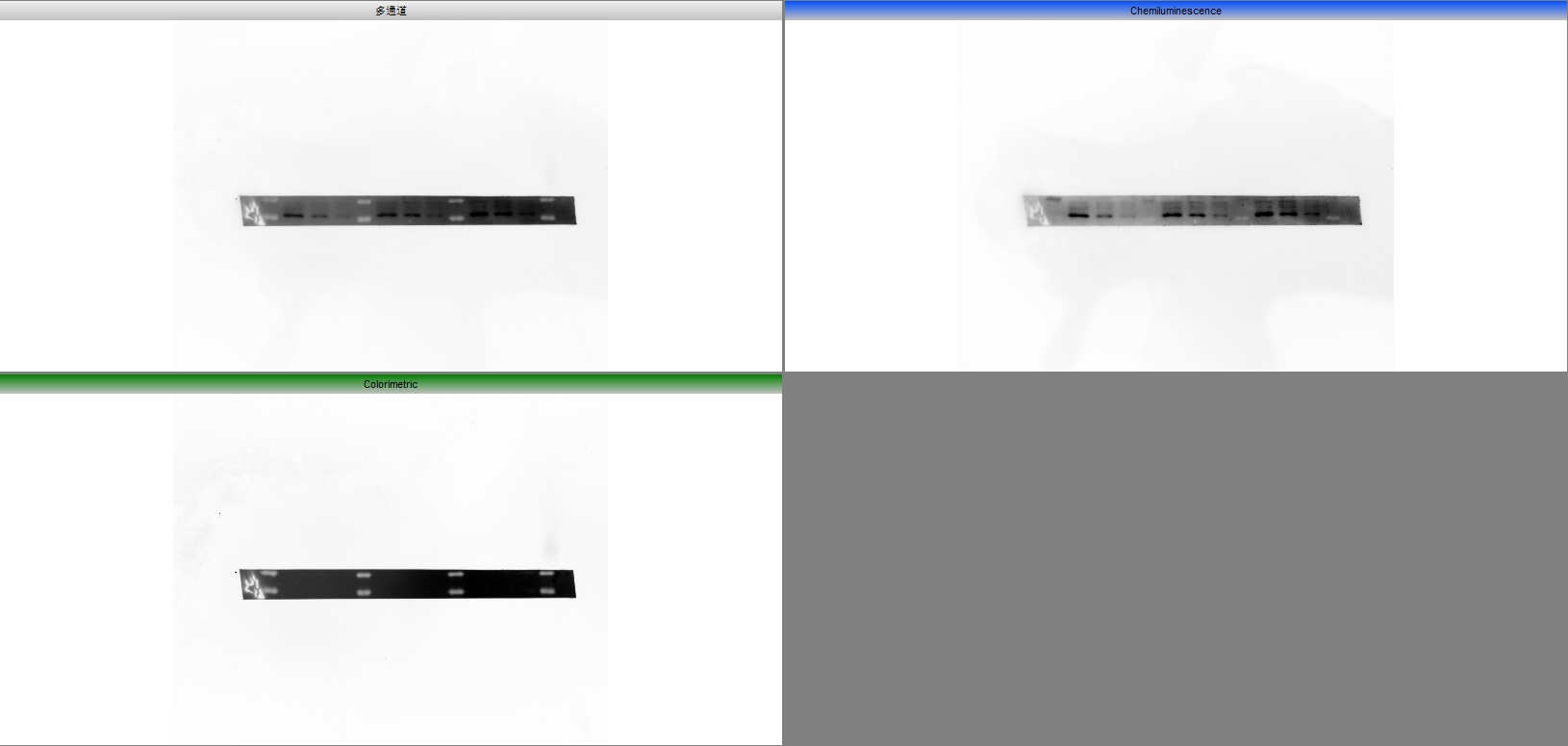

Supplement: Supplementary file 1 [file cancers-14-04809-s001.zip › cancers-1883913-Supplementary File S1. original whole blot/fig5/5637 Cyclin D1.jpg]

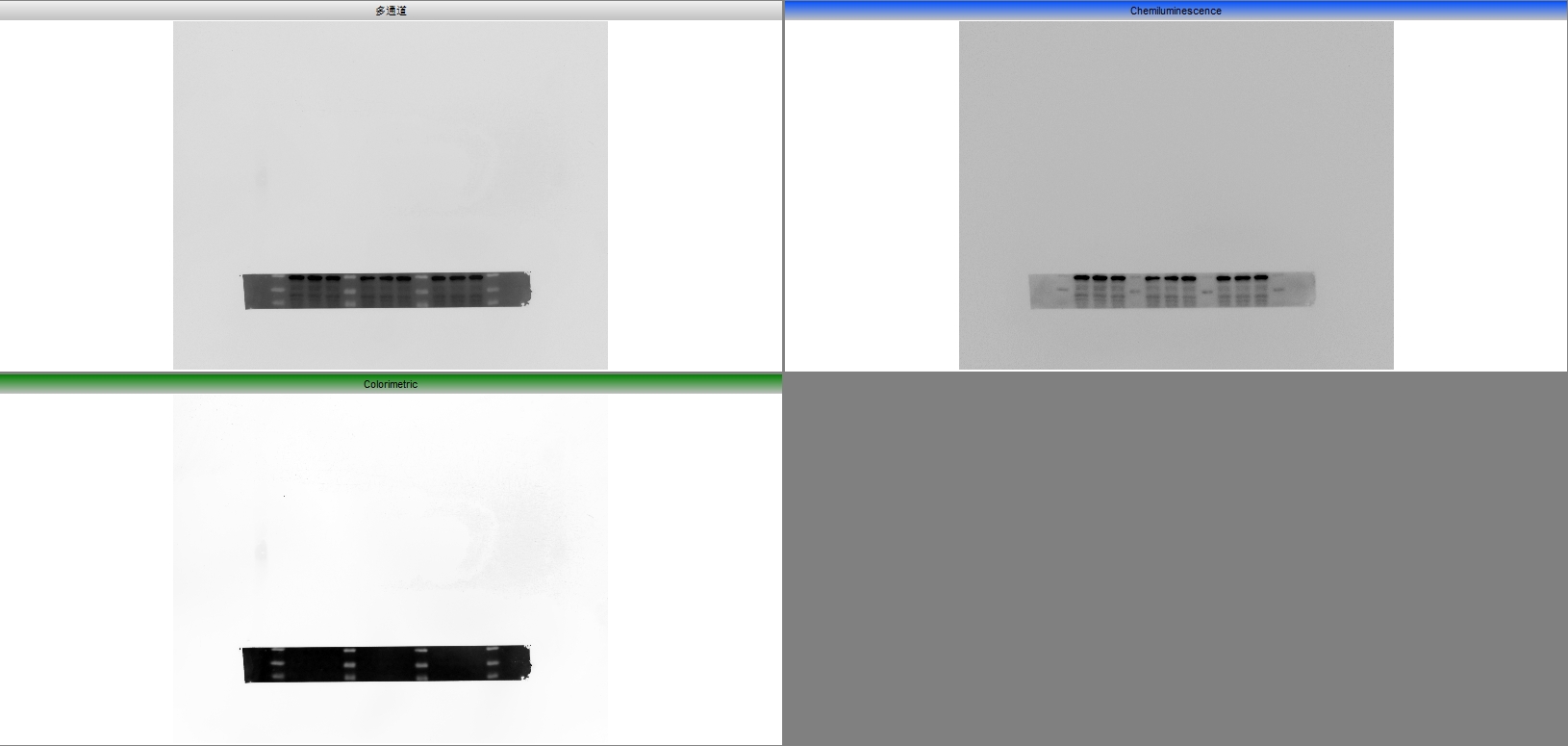

Supplement: Supplementary file 1 [file cancers-14-04809-s001.zip › cancers-1883913-Supplementary File S1. original whole blot/fig5/5637 GAPDH.jpg]

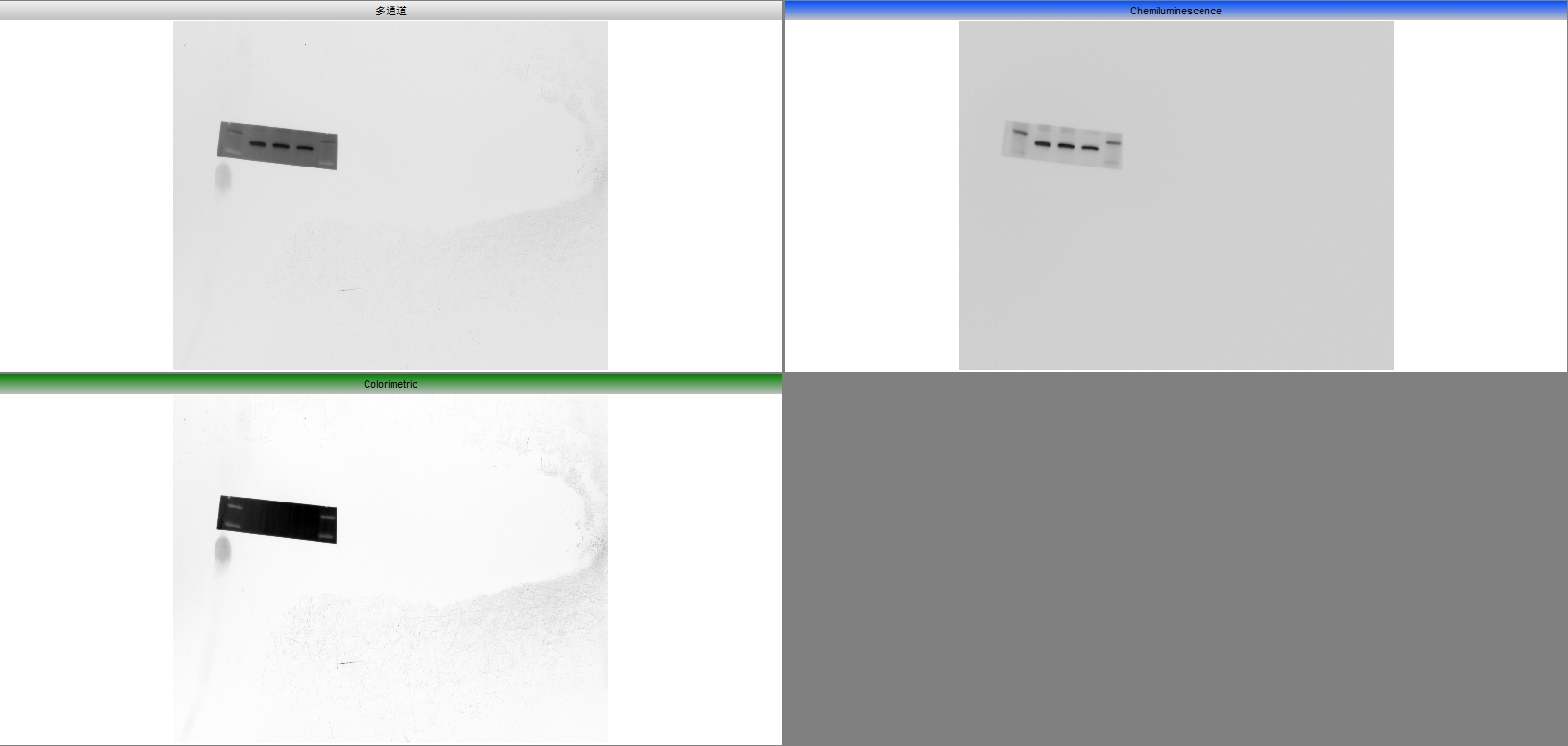

Supplement: Supplementary file 1 [file cancers-14-04809-s001.zip › cancers-1883913-Supplementary File S1. original whole blot/fig5/T24 Cyclin D1 1.jpg]

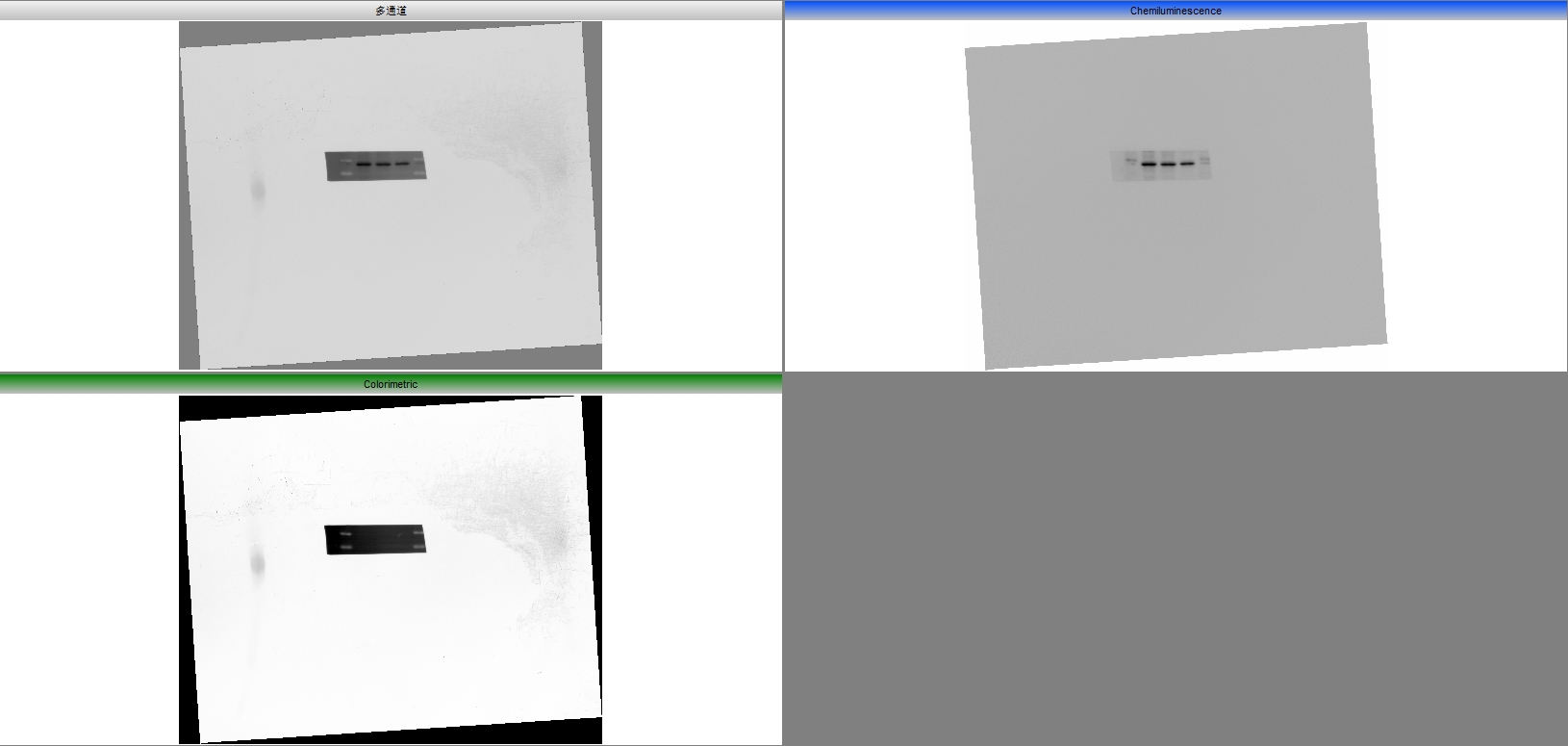

Supplement: Supplementary file 1 [file cancers-14-04809-s001.zip › cancers-1883913-Supplementary File S1. original whole blot/fig5/T24 Cyclin D1 2.jpg]

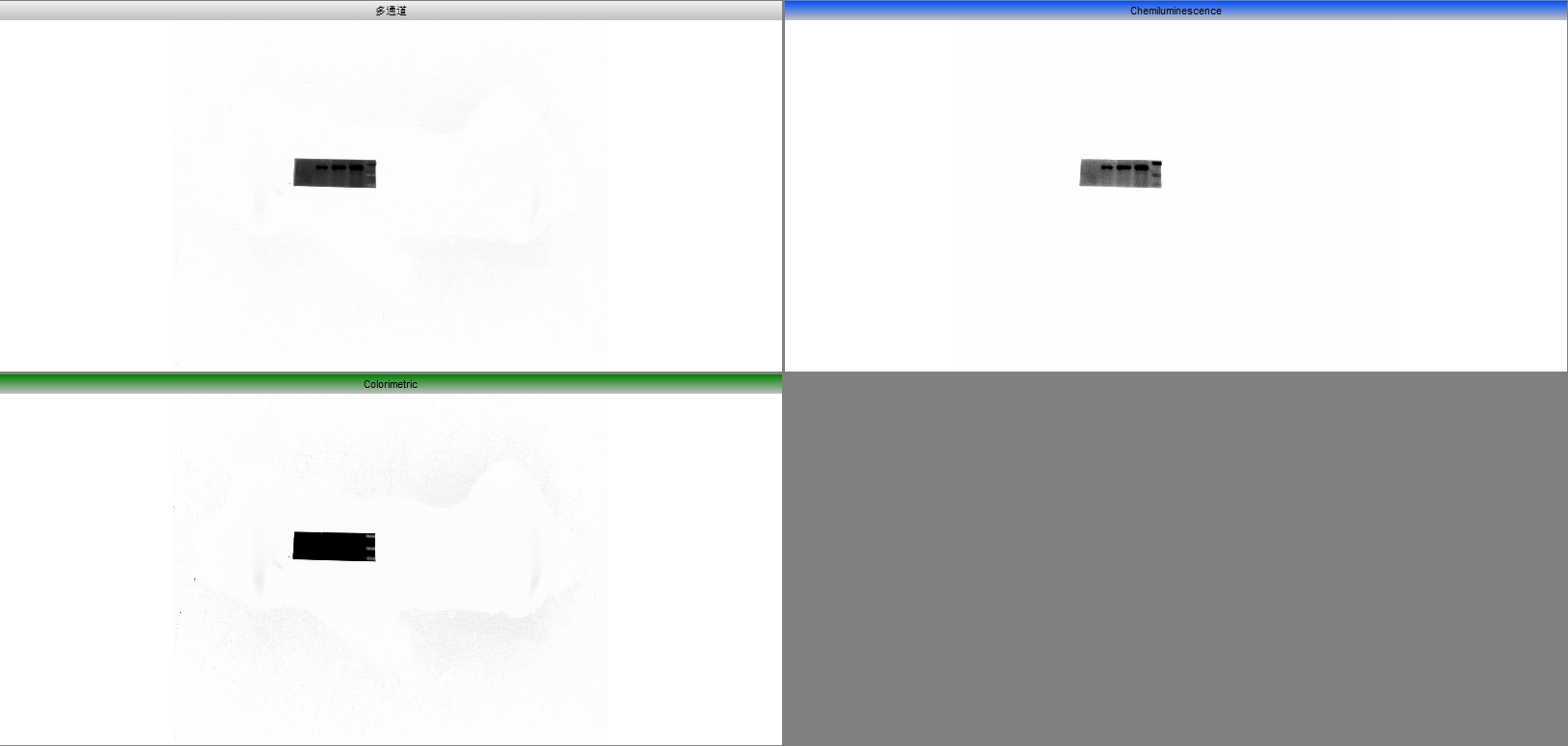

Supplement: Supplementary file 1 [file cancers-14-04809-s001.zip › cancers-1883913-Supplementary File S1. original whole blot/fig5/T24 Cyclin D1 3.jpg]

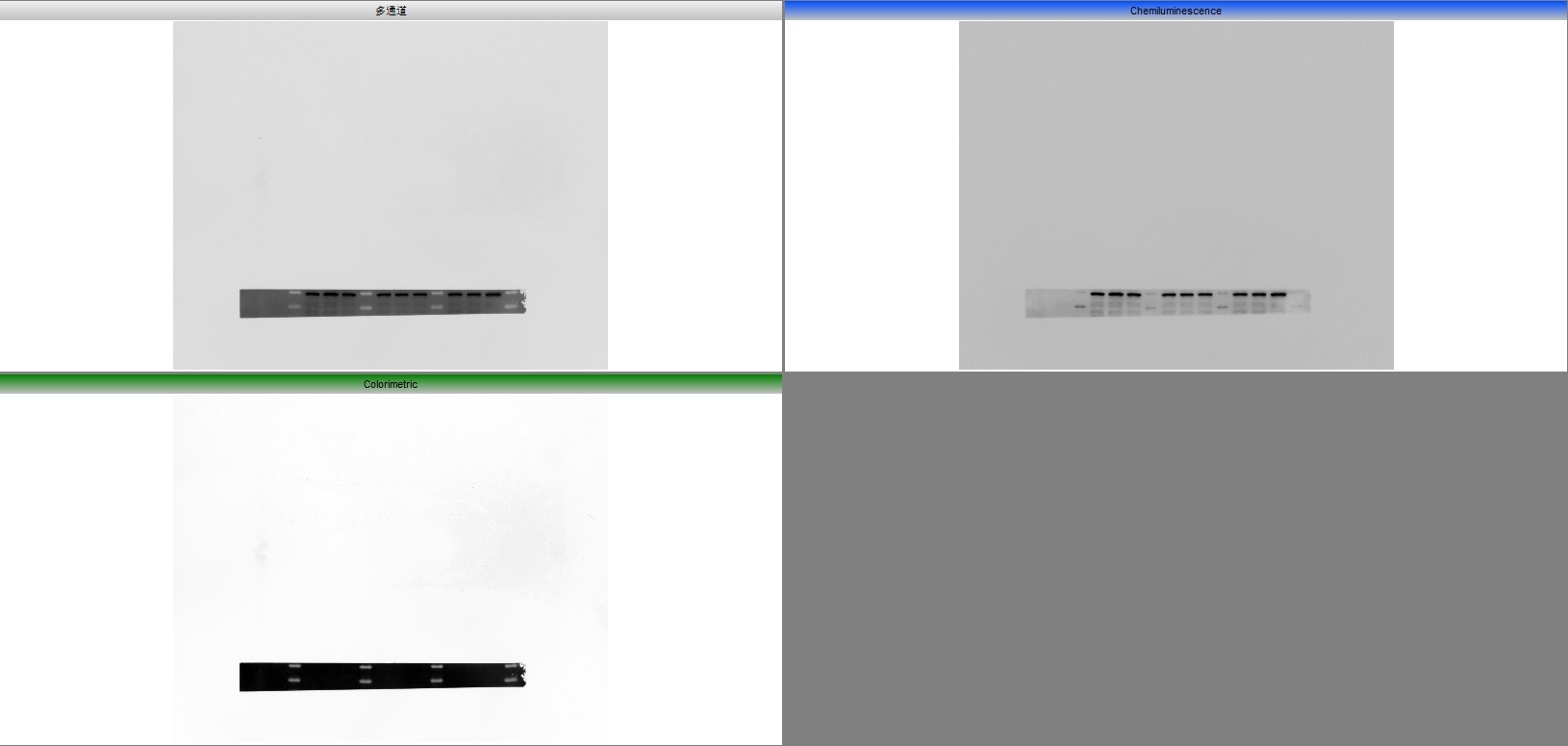

Supplement: Supplementary file 1 [file cancers-14-04809-s001.zip › cancers-1883913-Supplementary File S1. original whole blot/fig5/T24 GAPDH.jpg]

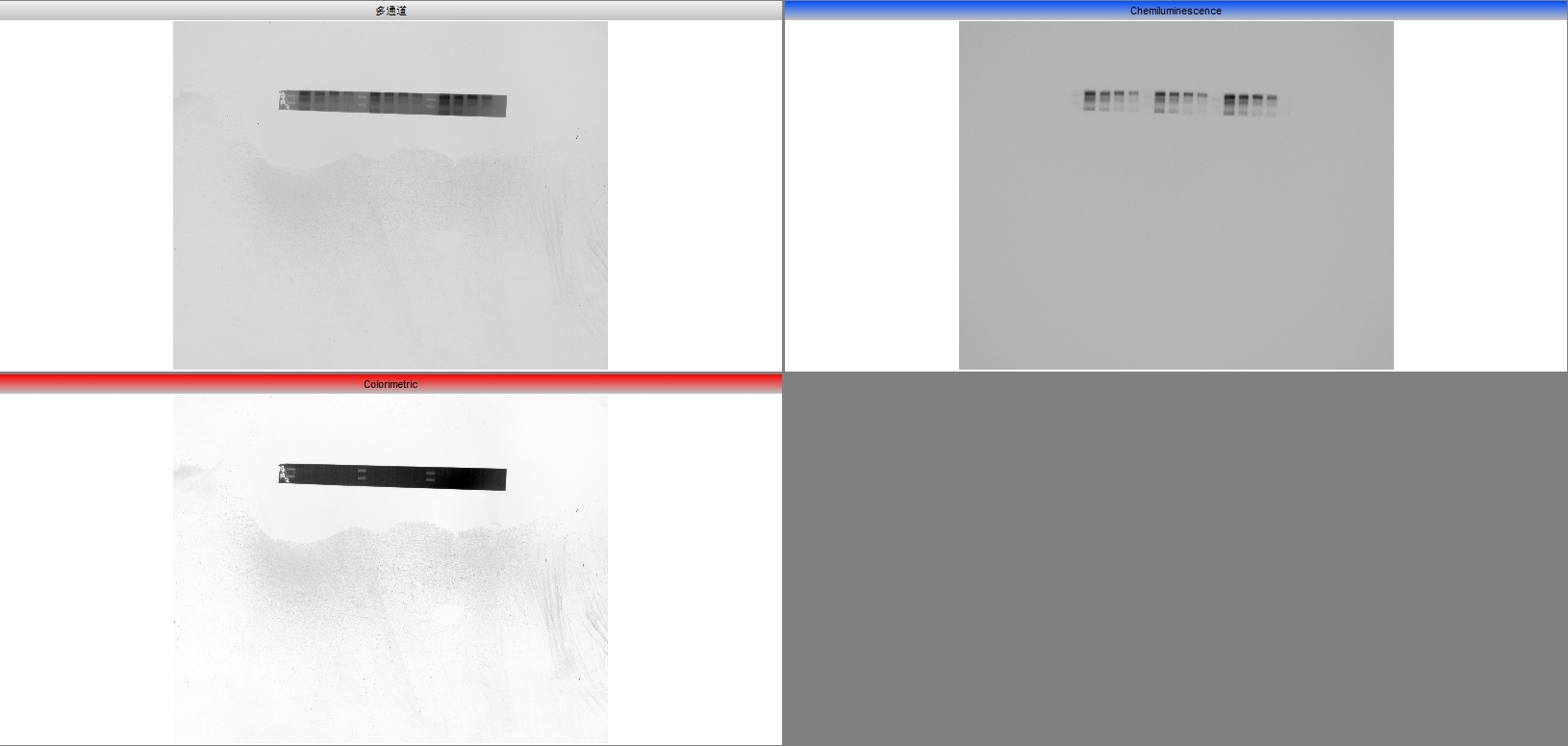

Supplement: Supplementary file 1 [file cancers-14-04809-s001.zip › cancers-1883913-Supplementary File S1. original whole blot/fig6/5637 EGFR.jpg]

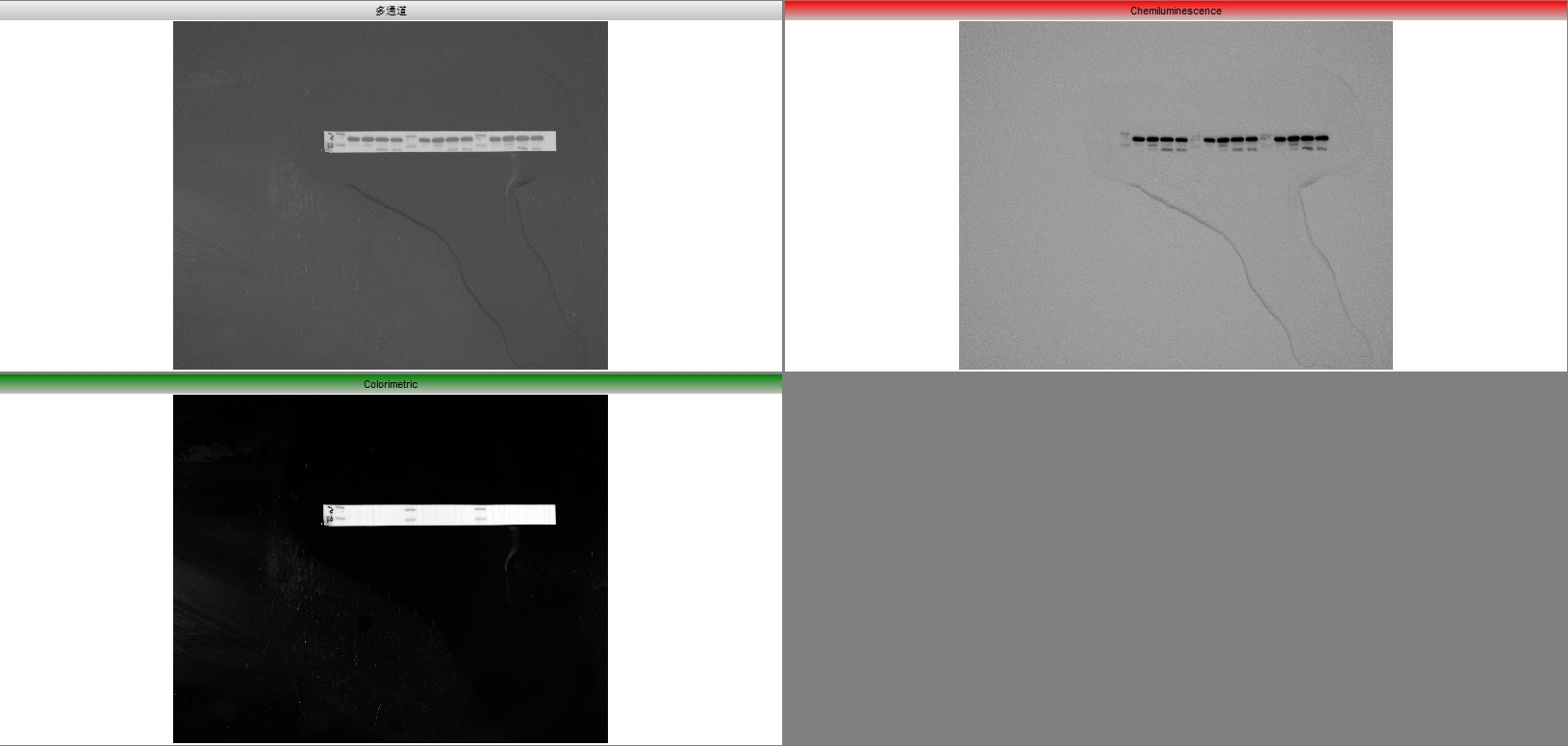

Supplement: Supplementary file 1 [file cancers-14-04809-s001.zip › cancers-1883913-Supplementary File S1. original whole blot/fig6/5637 GAPDH.jpg]

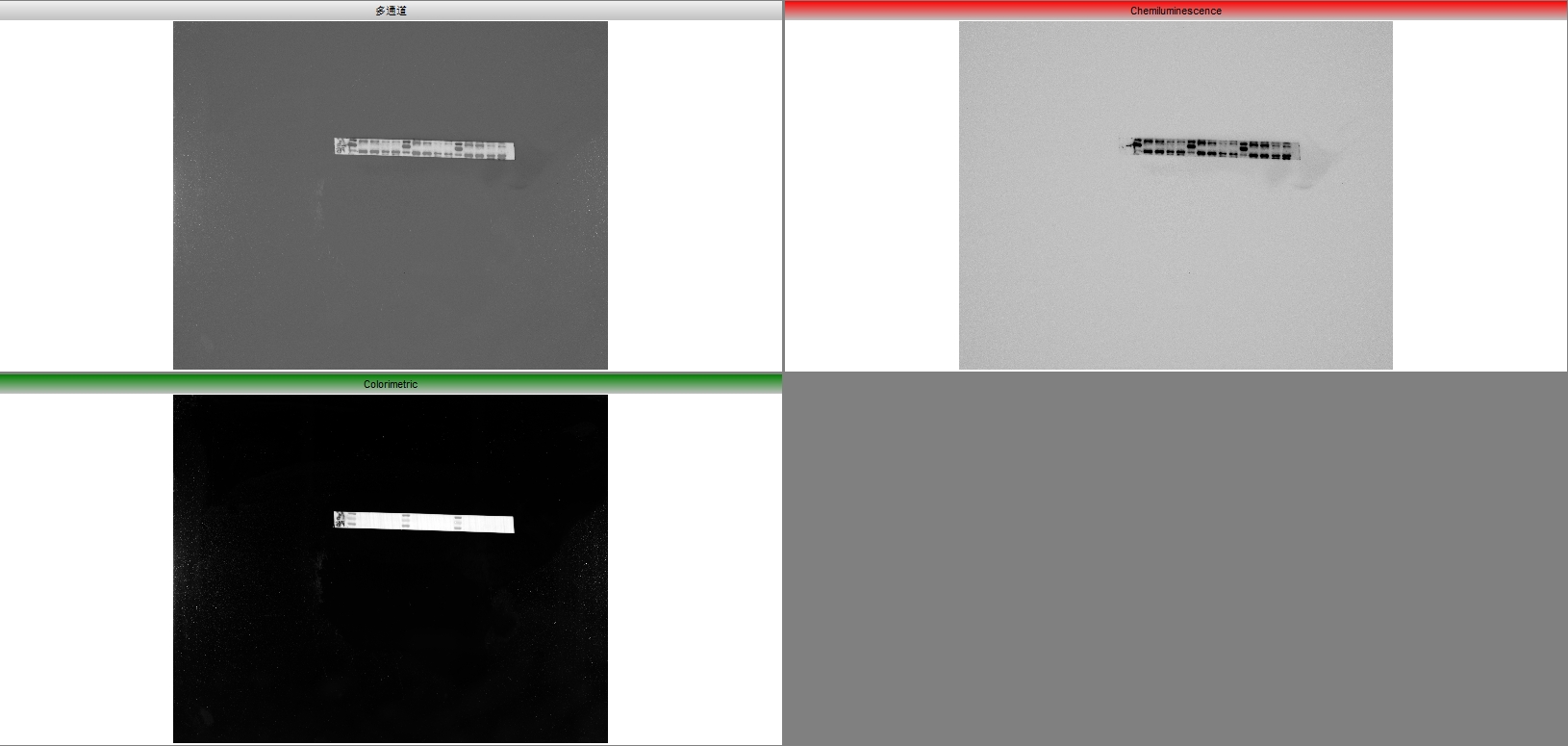

Supplement: Supplementary file 1 [file cancers-14-04809-s001.zip › cancers-1883913-Supplementary File S1. original whole blot/fig6/5637 NF-KB.jpg]

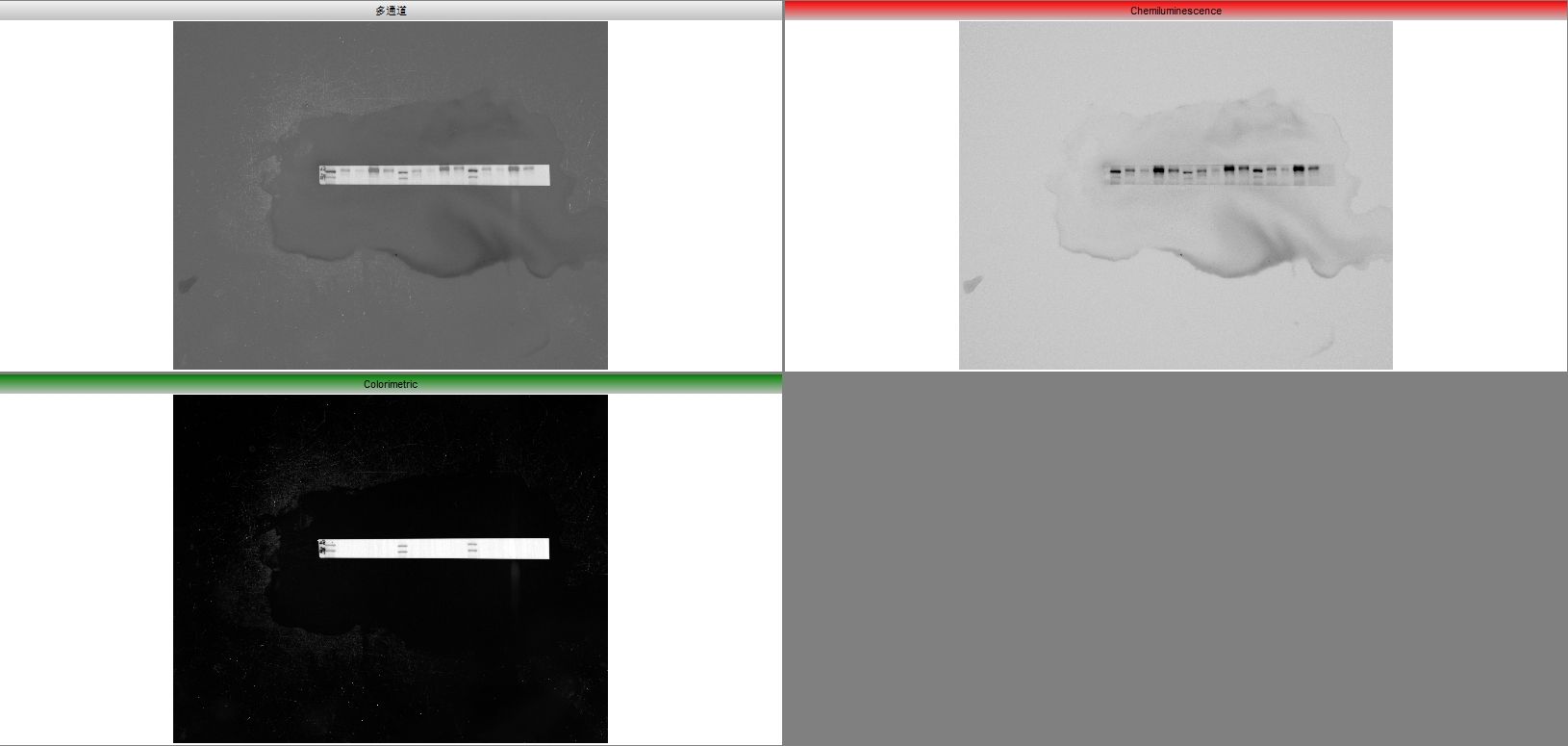

Supplement: Supplementary file 1 [file cancers-14-04809-s001.zip › cancers-1883913-Supplementary File S1. original whole blot/fig6/5637 pEGFR.jpg]

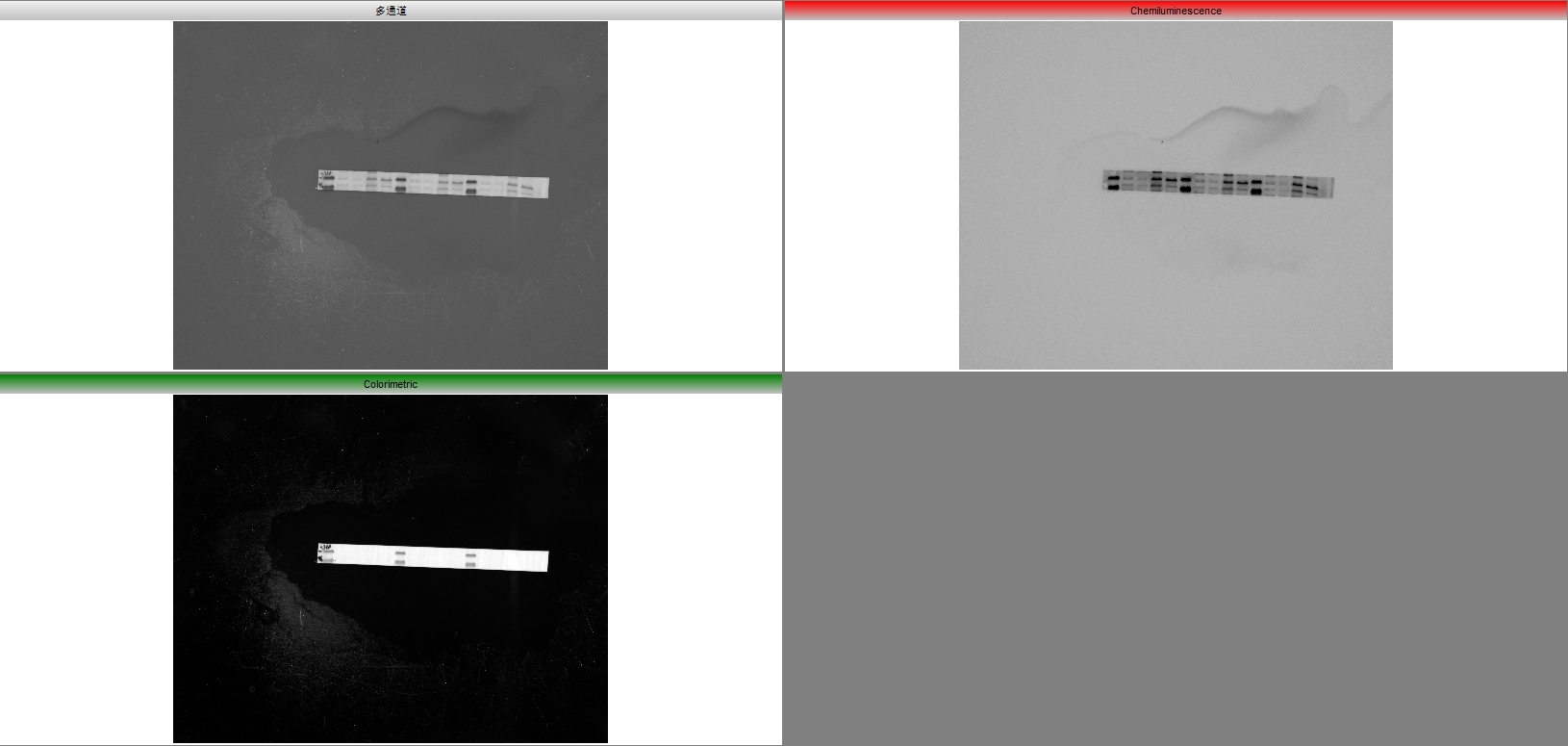

Supplement: Supplementary file 1 [file cancers-14-04809-s001.zip › cancers-1883913-Supplementary File S1. original whole blot/fig6/5637 pNF-KB.jpg]

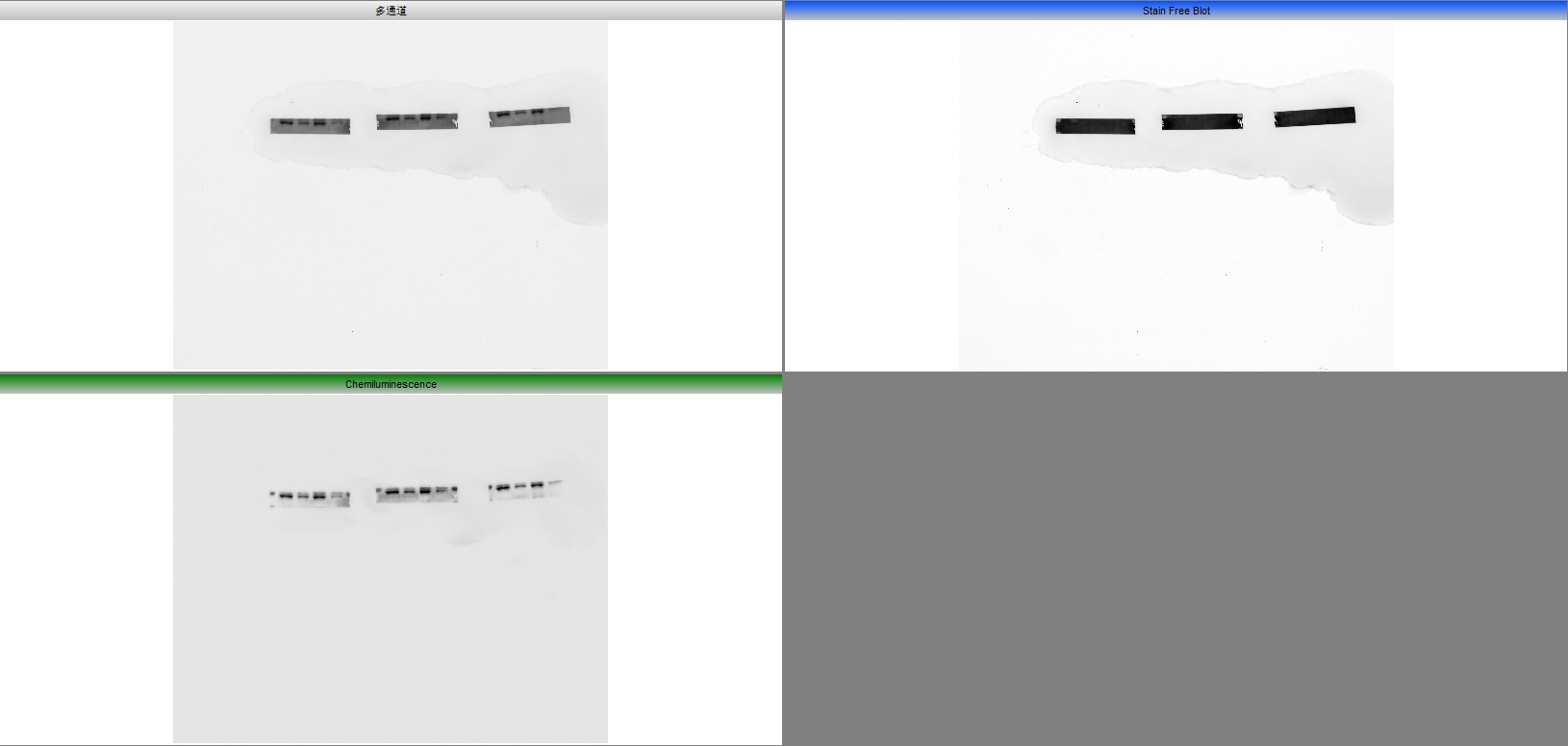

Supplement: Supplementary file 1 [file cancers-14-04809-s001.zip › cancers-1883913-Supplementary File S1. original whole blot/fig6/5637 TFAP2C.jpg]

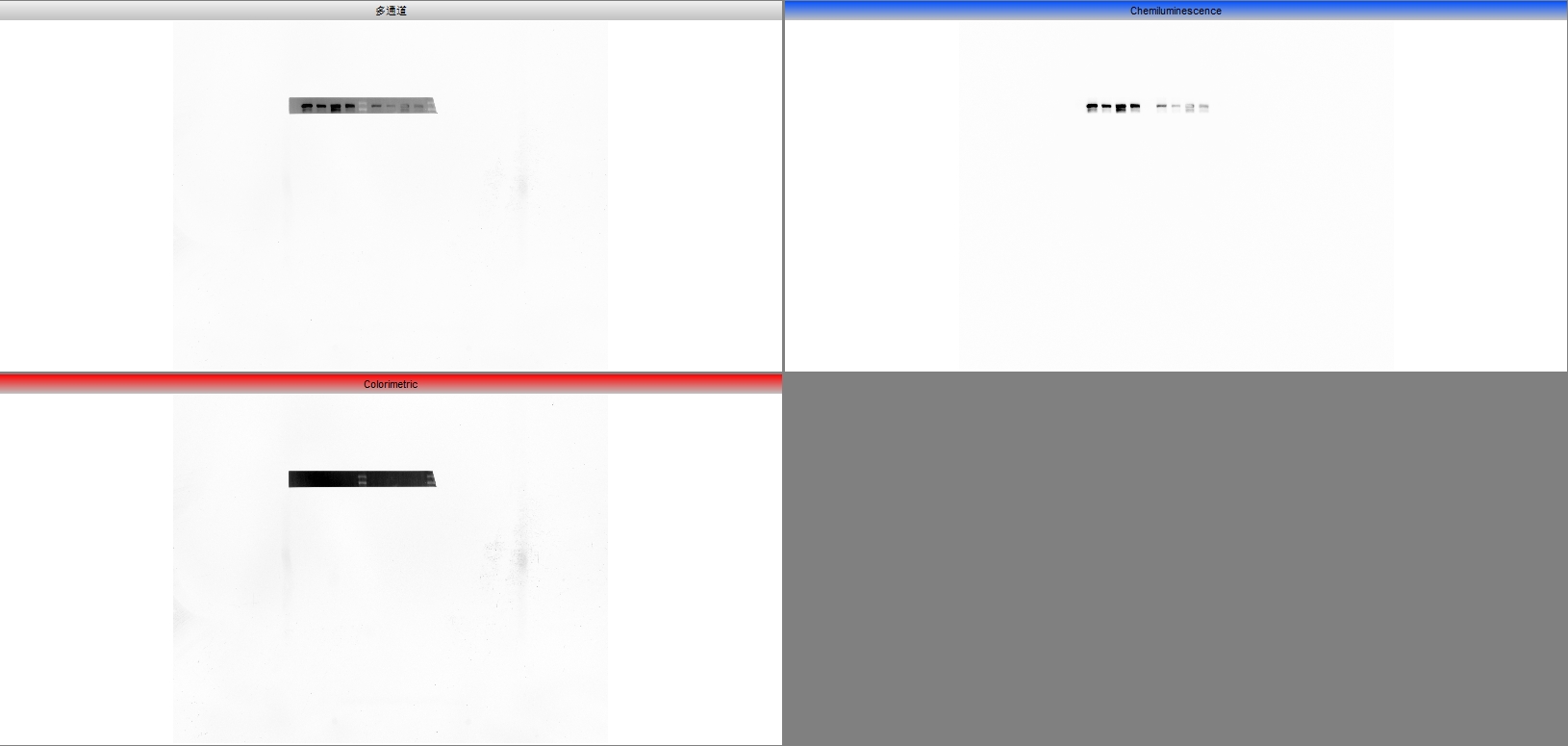

Supplement: Supplementary file 1 [file cancers-14-04809-s001.zip › cancers-1883913-Supplementary File S1. original whole blot/fig6/T24 EGFR1.jpg]

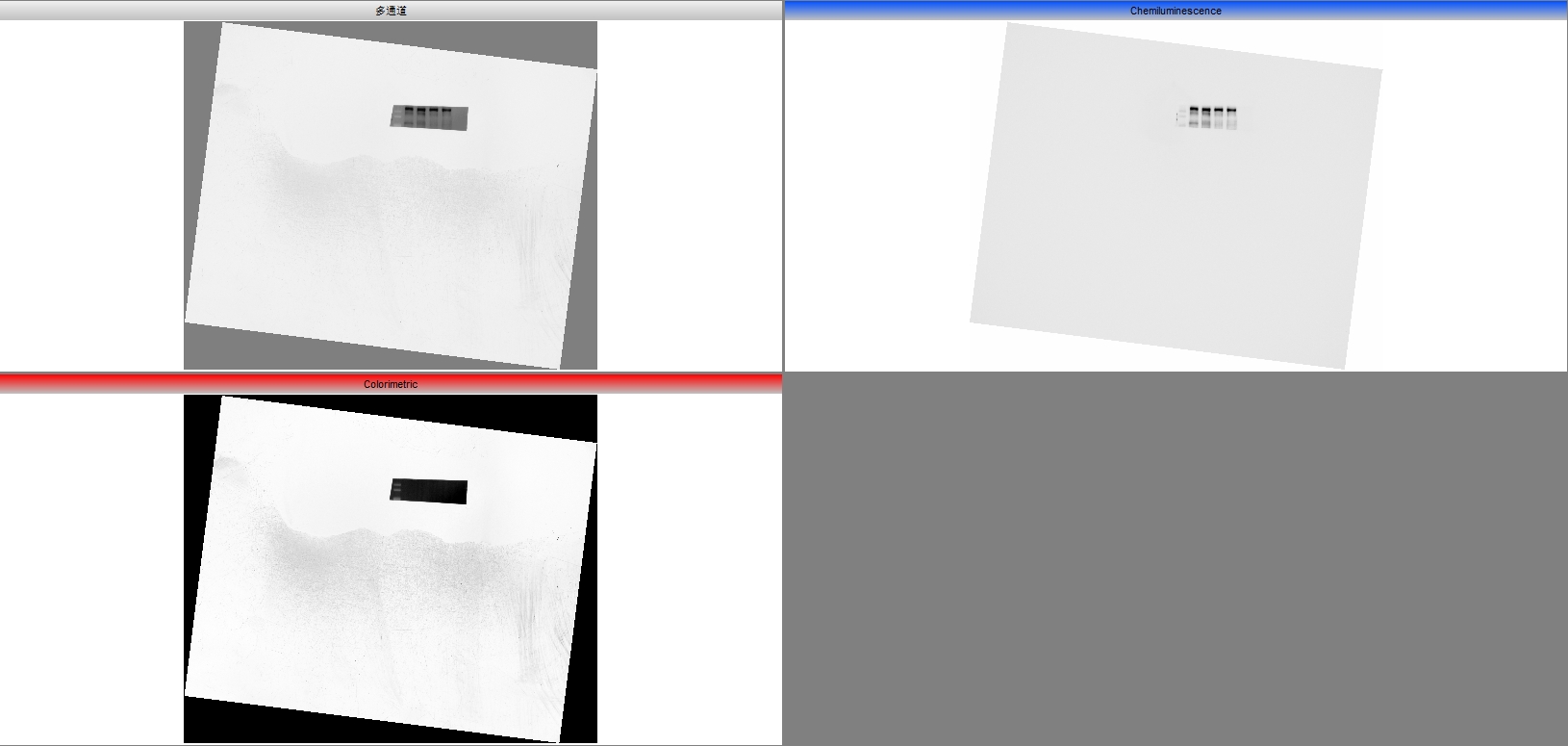

Supplement: Supplementary file 1 [file cancers-14-04809-s001.zip › cancers-1883913-Supplementary File S1. original whole blot/fig6/T24 EGFR2.jpg]

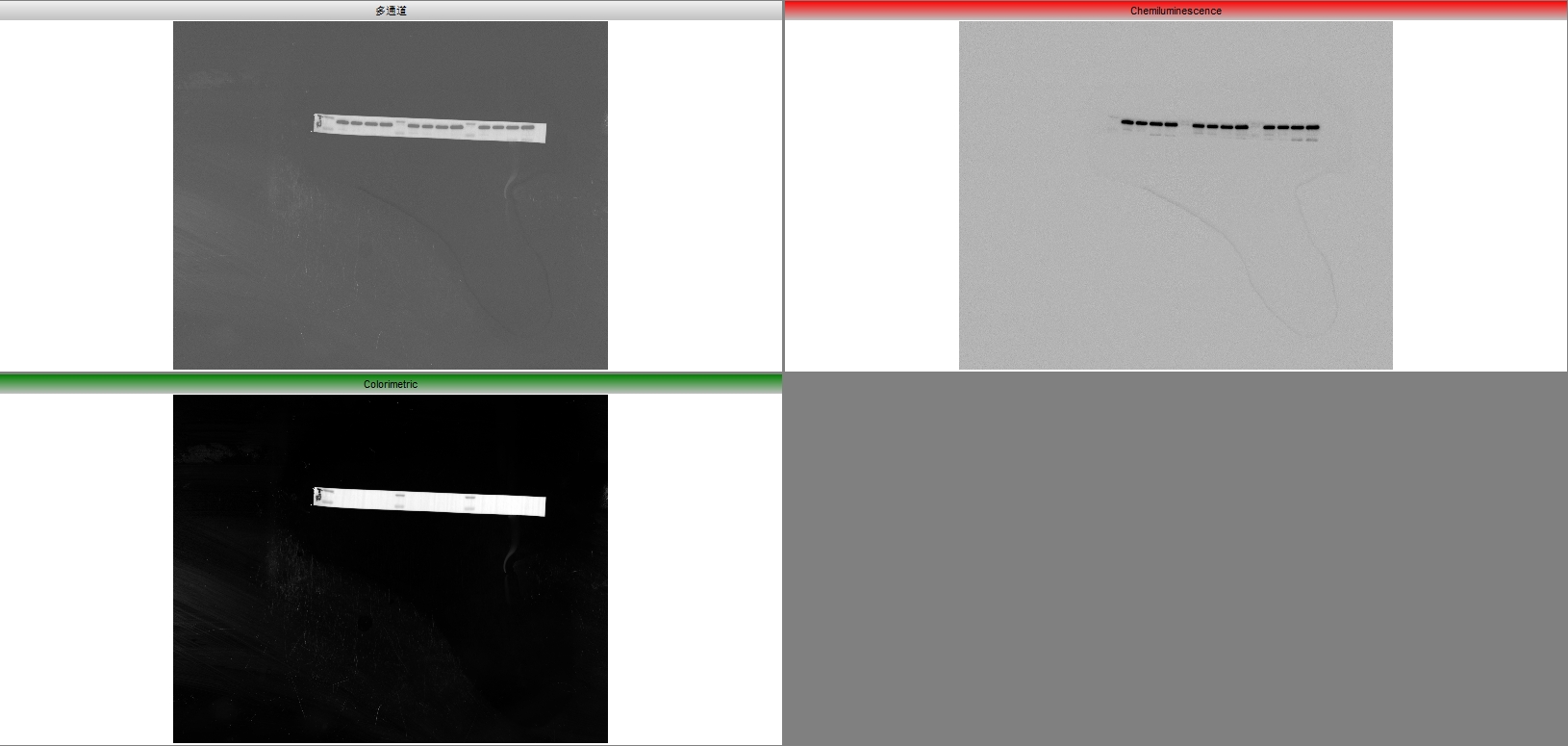

Supplement: Supplementary file 1 [file cancers-14-04809-s001.zip › cancers-1883913-Supplementary File S1. original whole blot/fig6/T24 GAPDH.jpg]

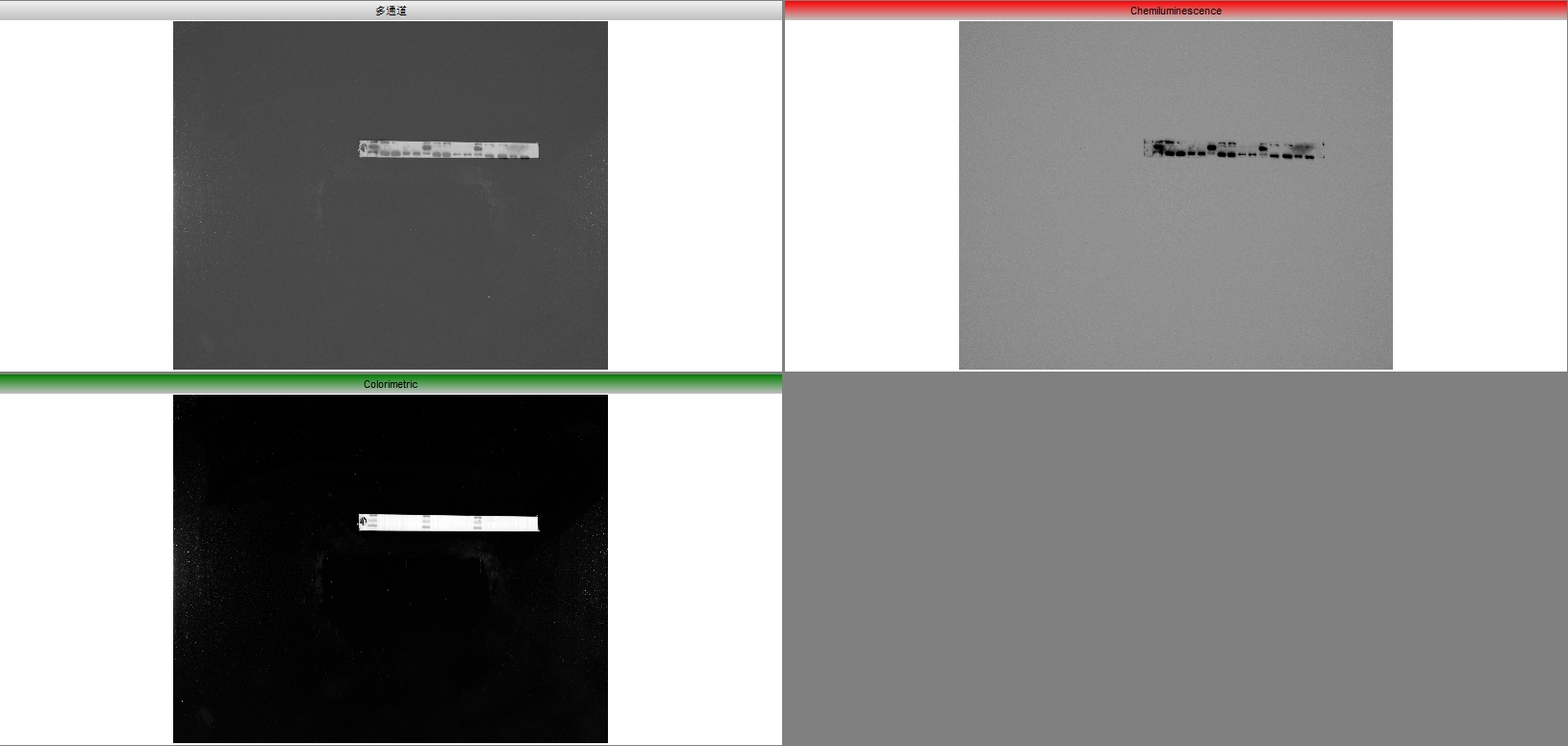

Supplement: Supplementary file 1 [file cancers-14-04809-s001.zip › cancers-1883913-Supplementary File S1. original whole blot/fig6/T24 NF-KB.jpg]

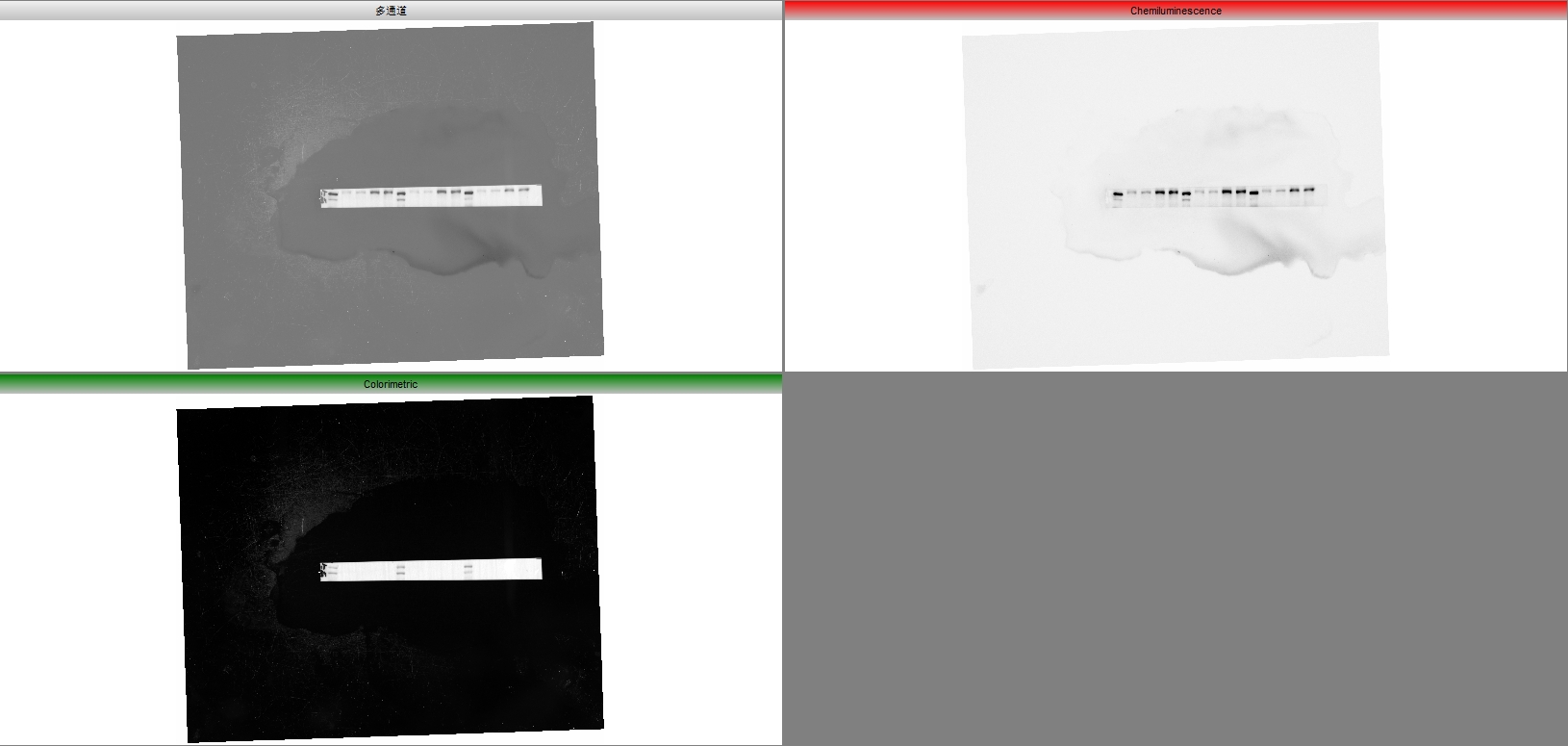

Supplement: Supplementary file 1 [file cancers-14-04809-s001.zip › cancers-1883913-Supplementary File S1. original whole blot/fig6/T24 pEGFR.jpg]

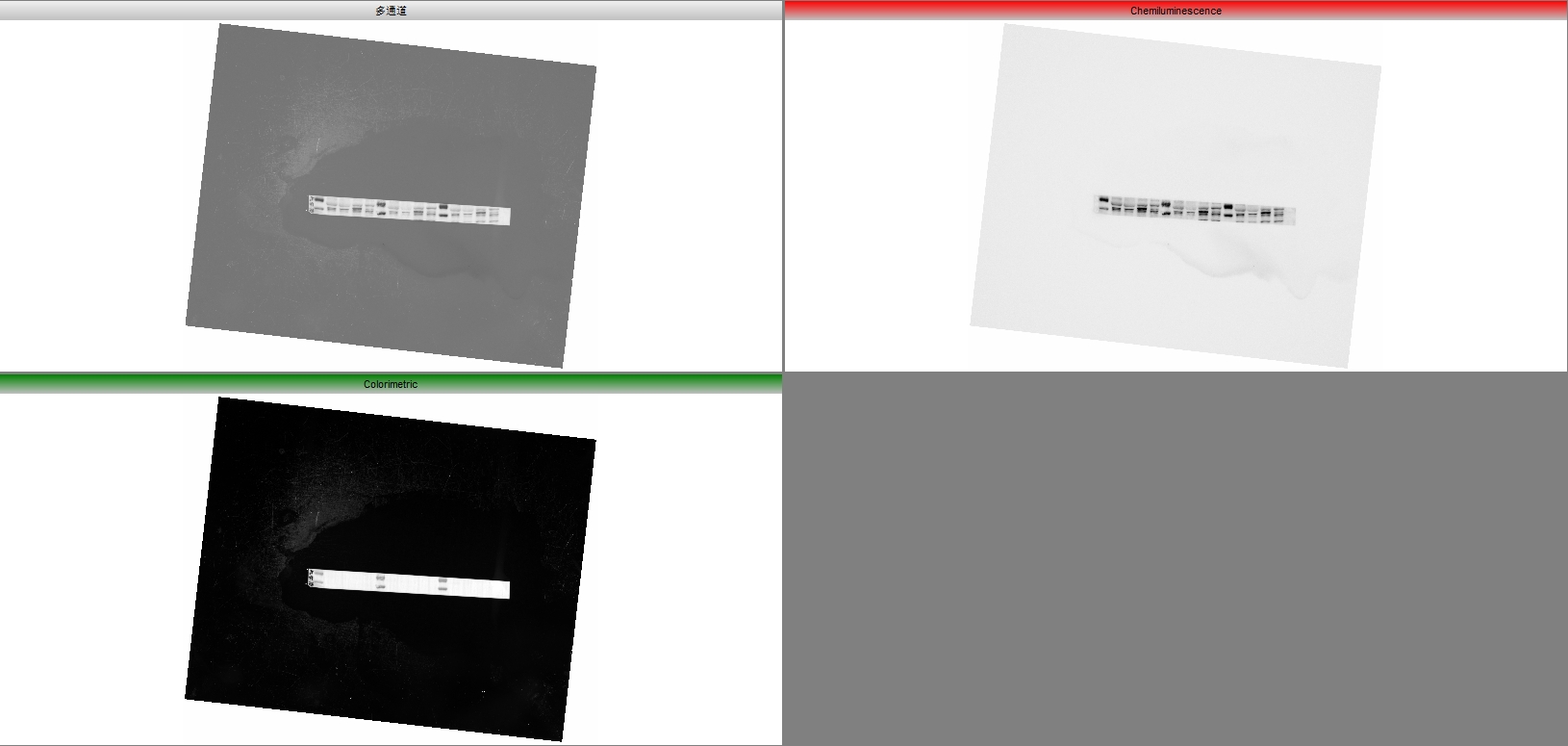

Supplement: Supplementary file 1 [file cancers-14-04809-s001.zip › cancers-1883913-Supplementary File S1. original whole blot/fig6/T24 pNF-KB.jpg]

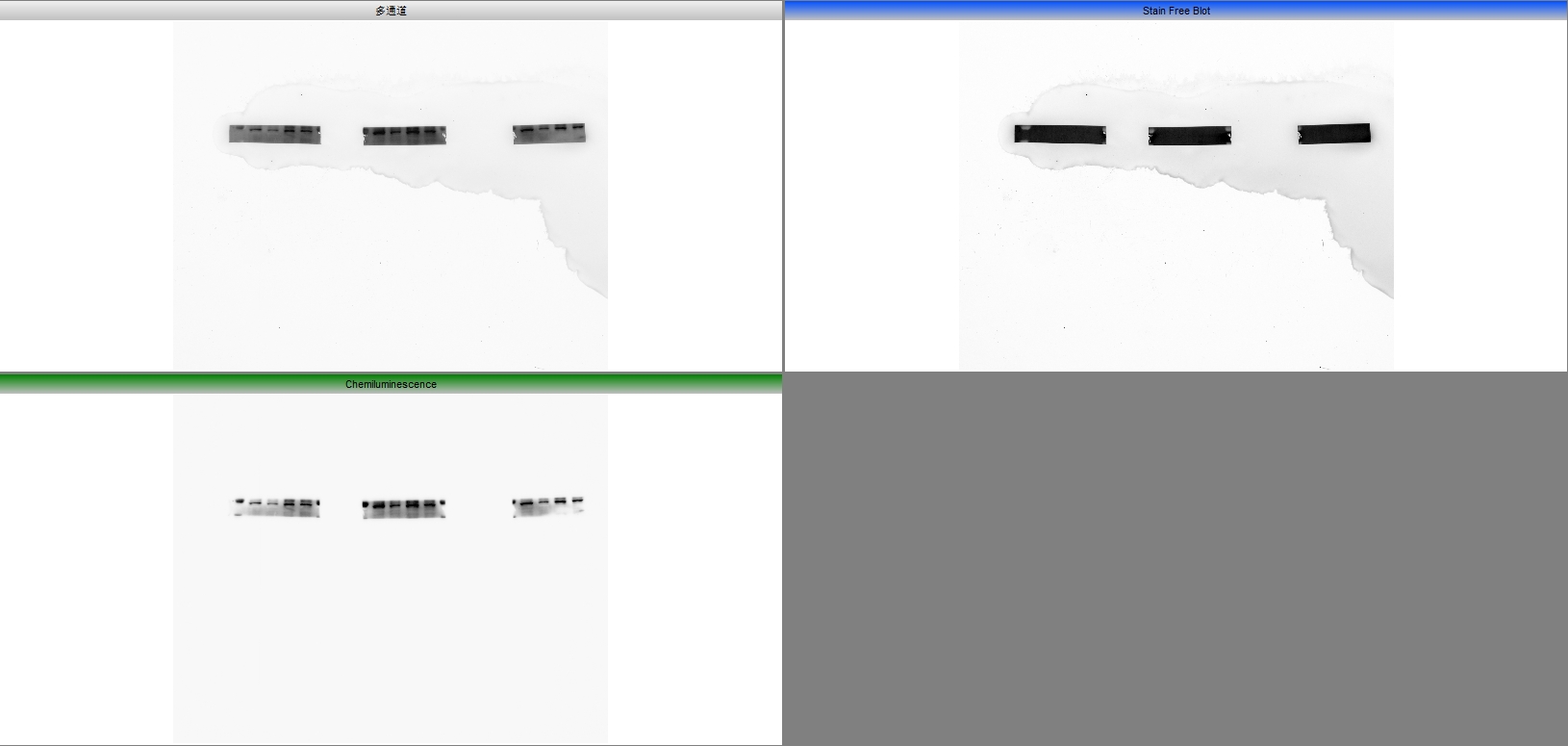

Supplement: Supplementary file 1 [file cancers-14-04809-s001.zip › cancers-1883913-Supplementary File S1. original whole blot/fig6/T24 TFAP2C.jpg]

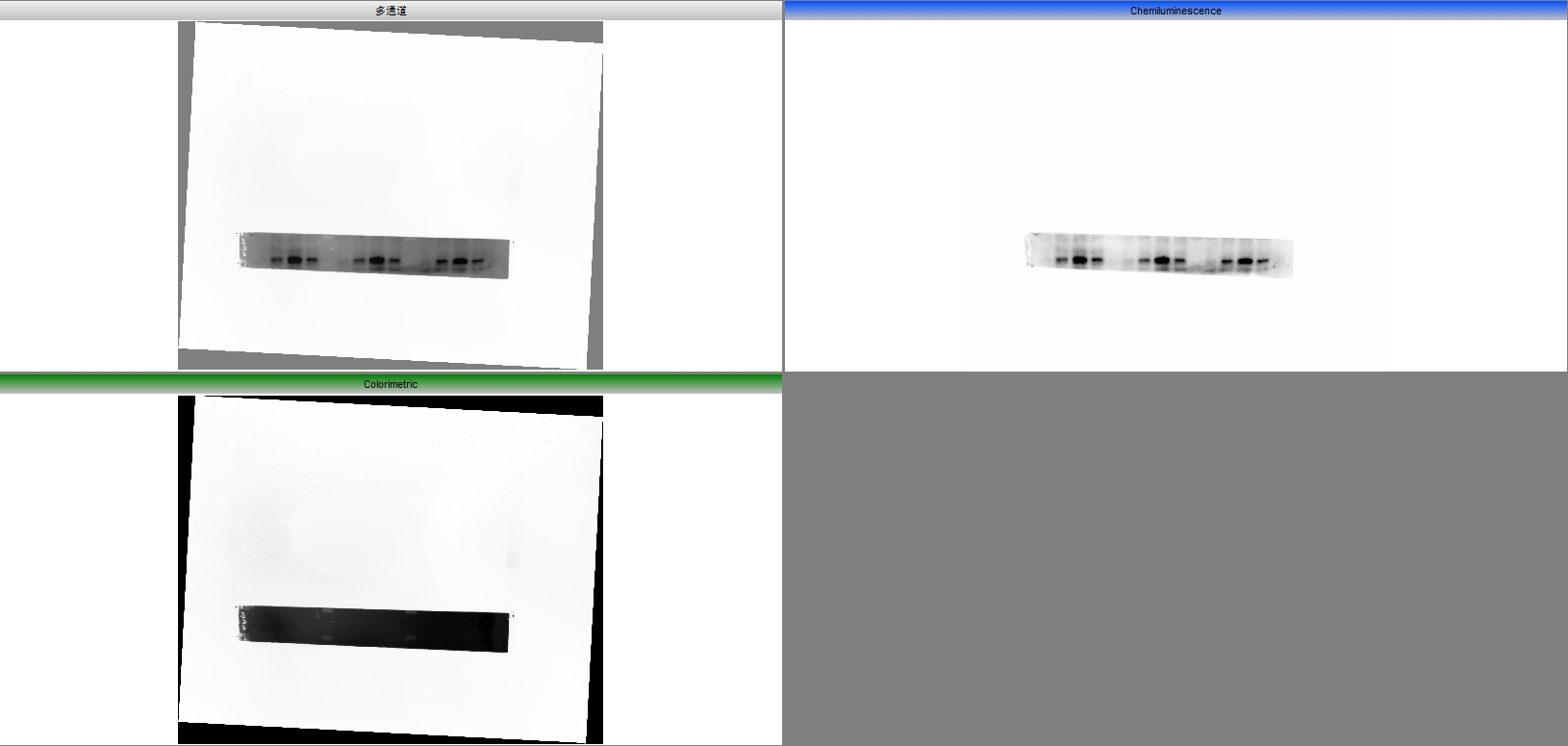

Supplement: Supplementary file 1 [file cancers-14-04809-s001.zip › cancers-1883913-Supplementary File S1. original whole blot/fig7/5637 Cleaved caspase-3.jpg]

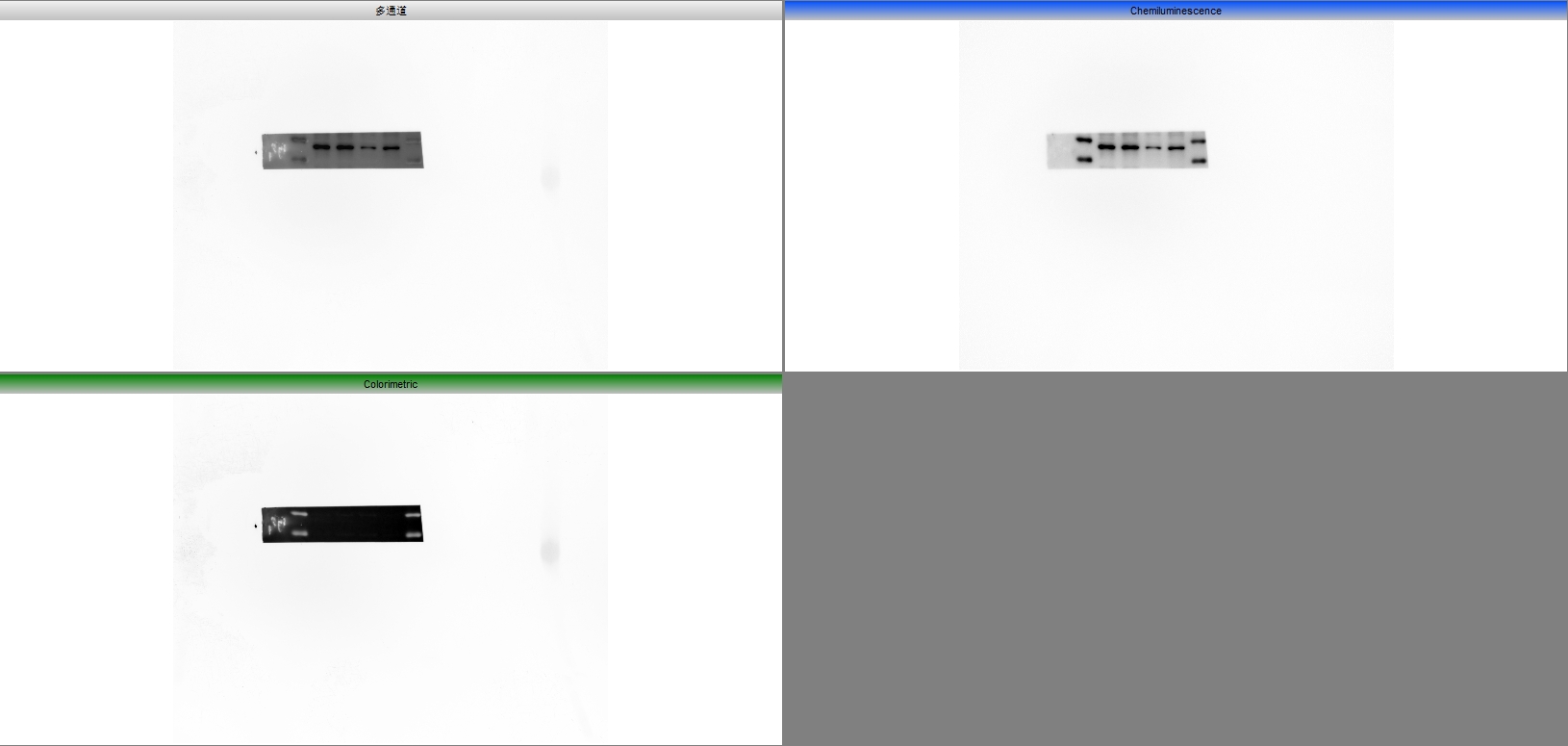

Supplement: Supplementary file 1 [file cancers-14-04809-s001.zip › cancers-1883913-Supplementary File S1. original whole blot/fig7/5637 Cyclin D1 2.jpg]

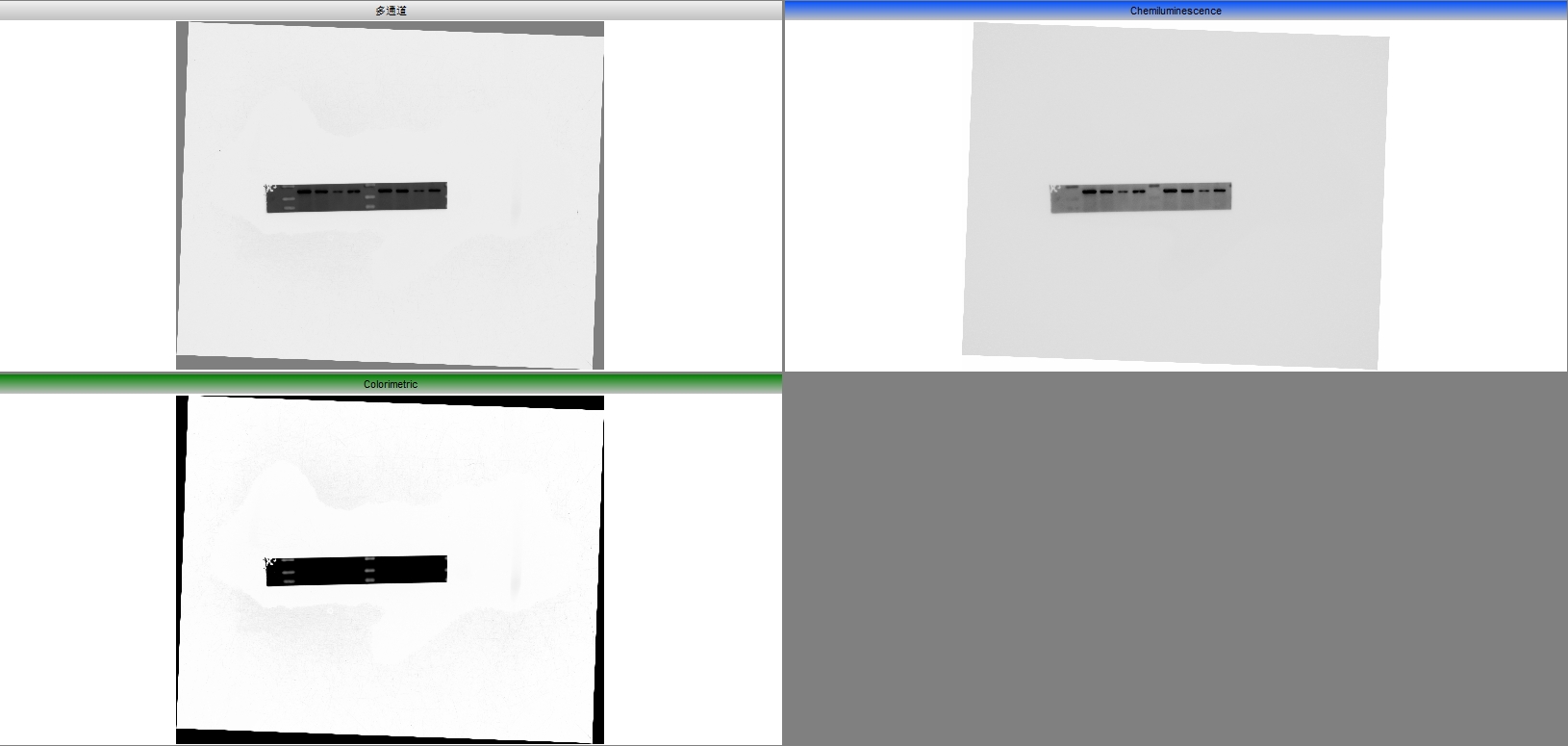

Supplement: Supplementary file 1 [file cancers-14-04809-s001.zip › cancers-1883913-Supplementary File S1. original whole blot/fig7/5637 Cyclin D1.jpg]

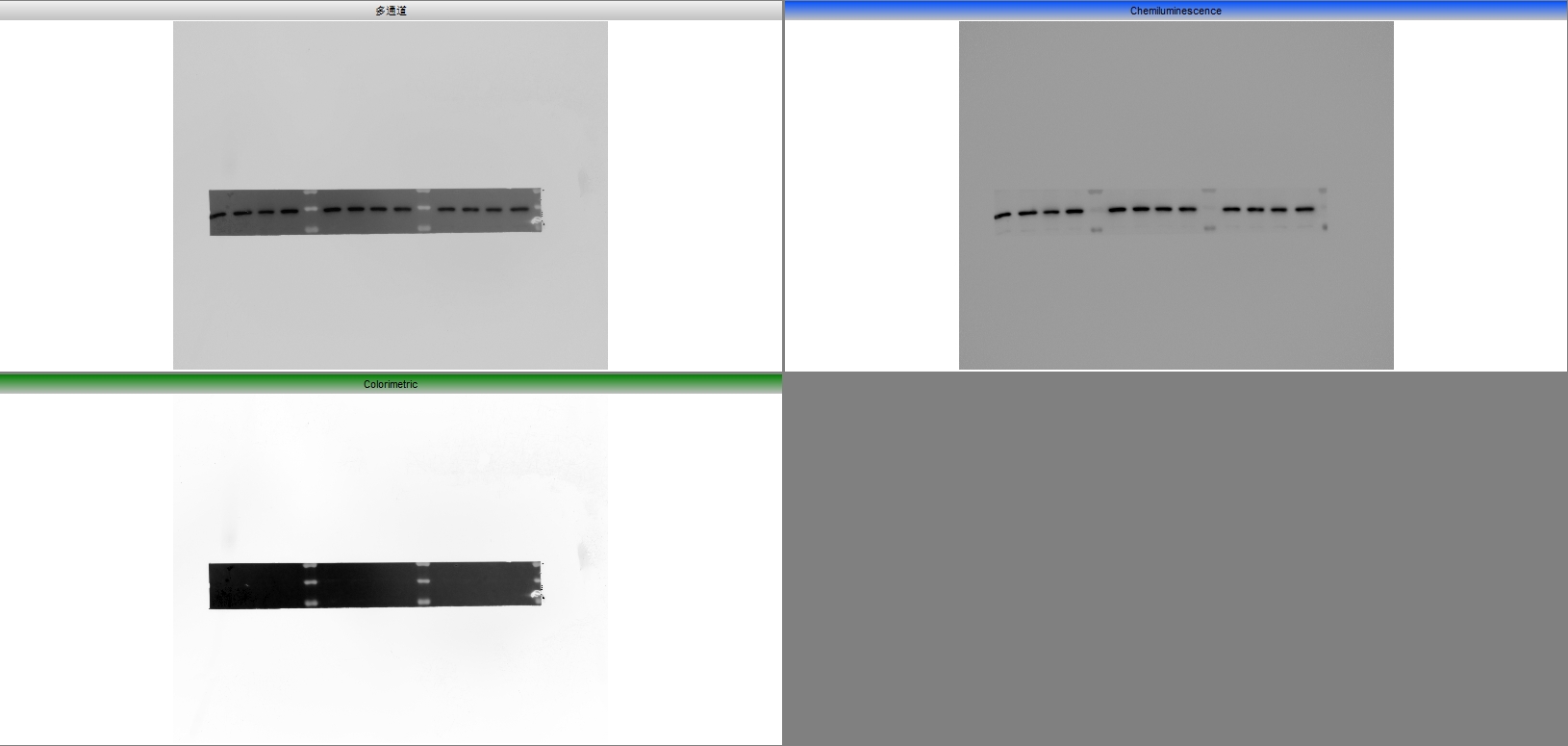

Supplement: Supplementary file 1 [file cancers-14-04809-s001.zip › cancers-1883913-Supplementary File S1. original whole blot/fig7/5637 GAPDH.jpg]

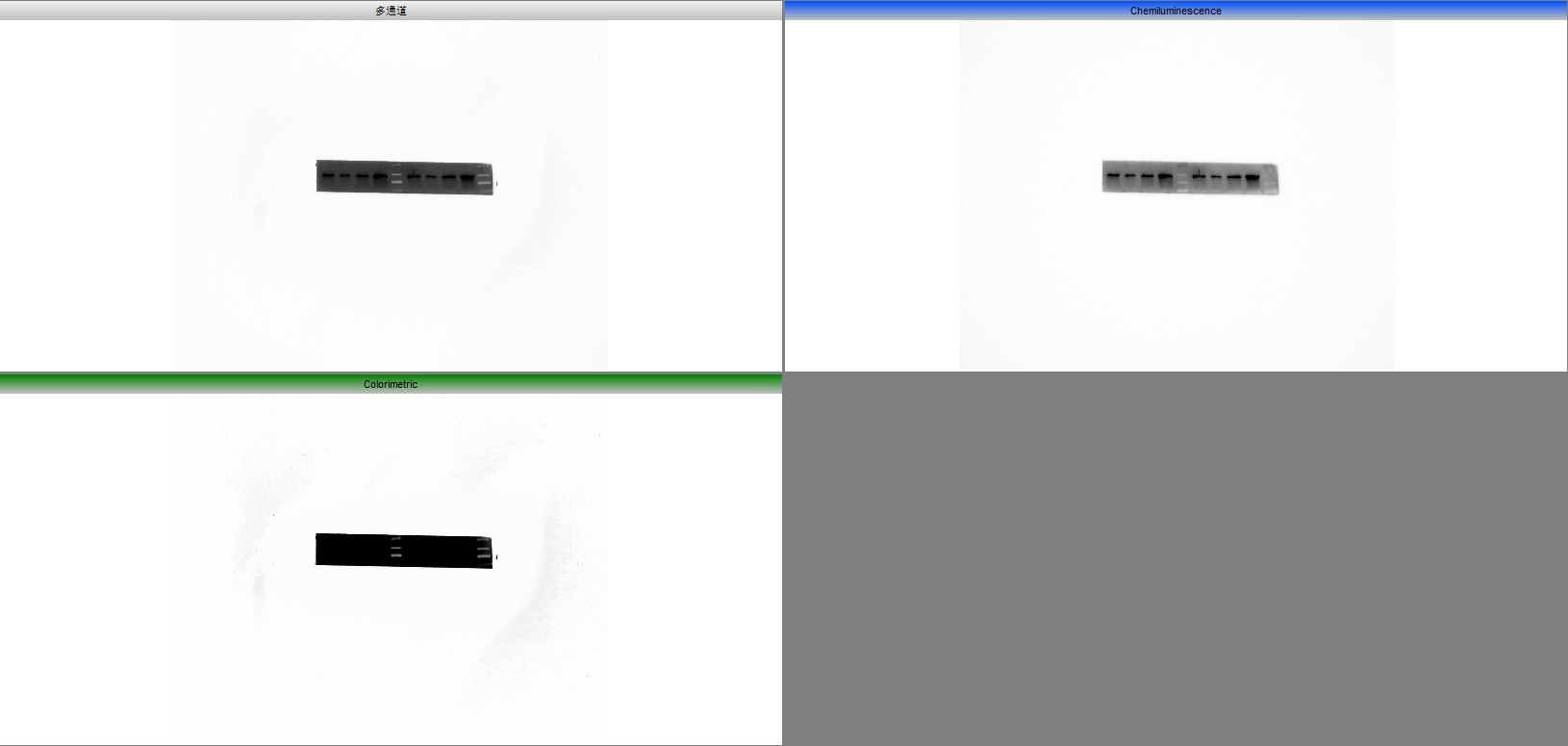

Supplement: Supplementary file 1 [file cancers-14-04809-s001.zip › cancers-1883913-Supplementary File S1. original whole blot/fig7/5637 N-cadhern 2.jpg]

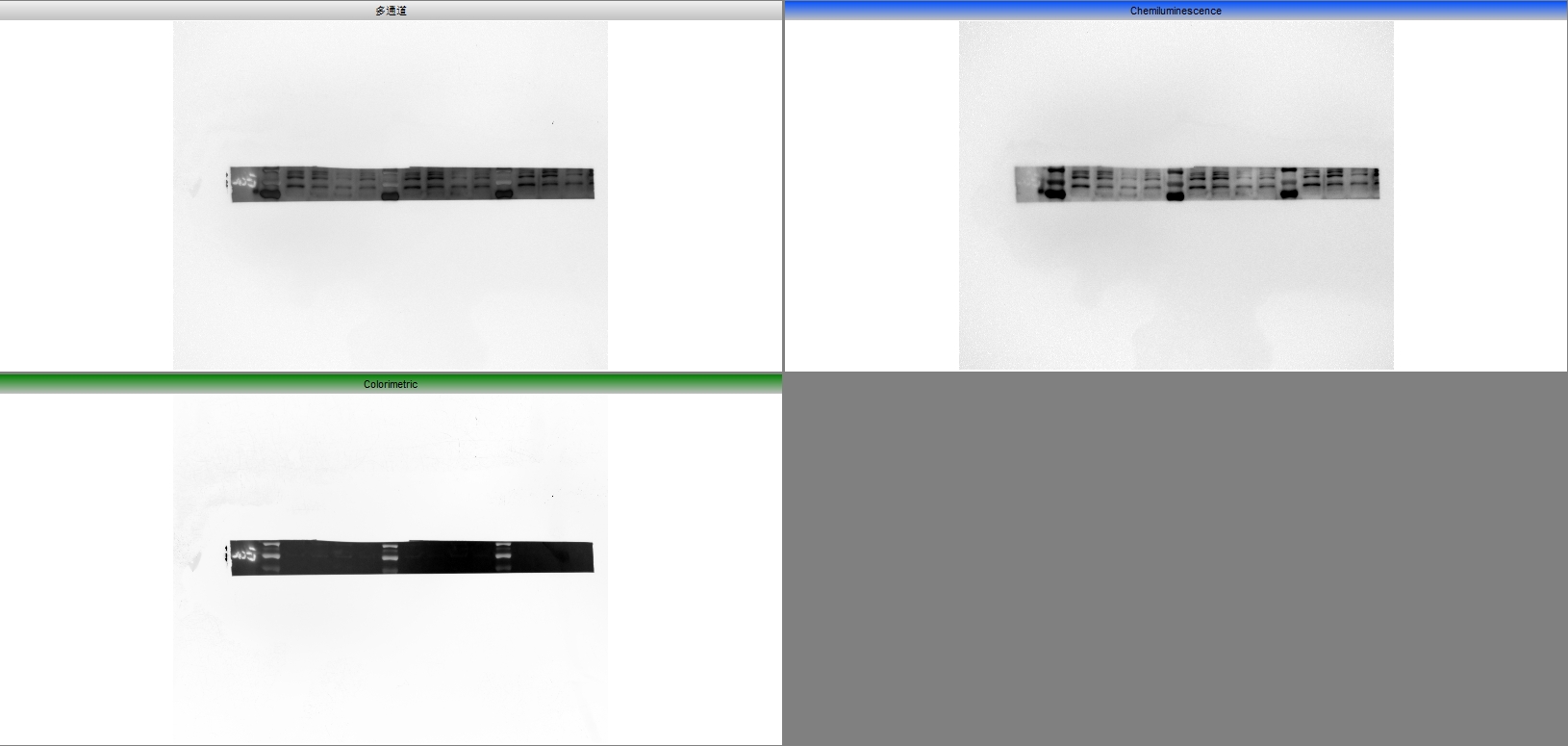

Supplement: Supplementary file 1 [file cancers-14-04809-s001.zip › cancers-1883913-Supplementary File S1. original whole blot/fig7/5637 N-cadhern.jpg]

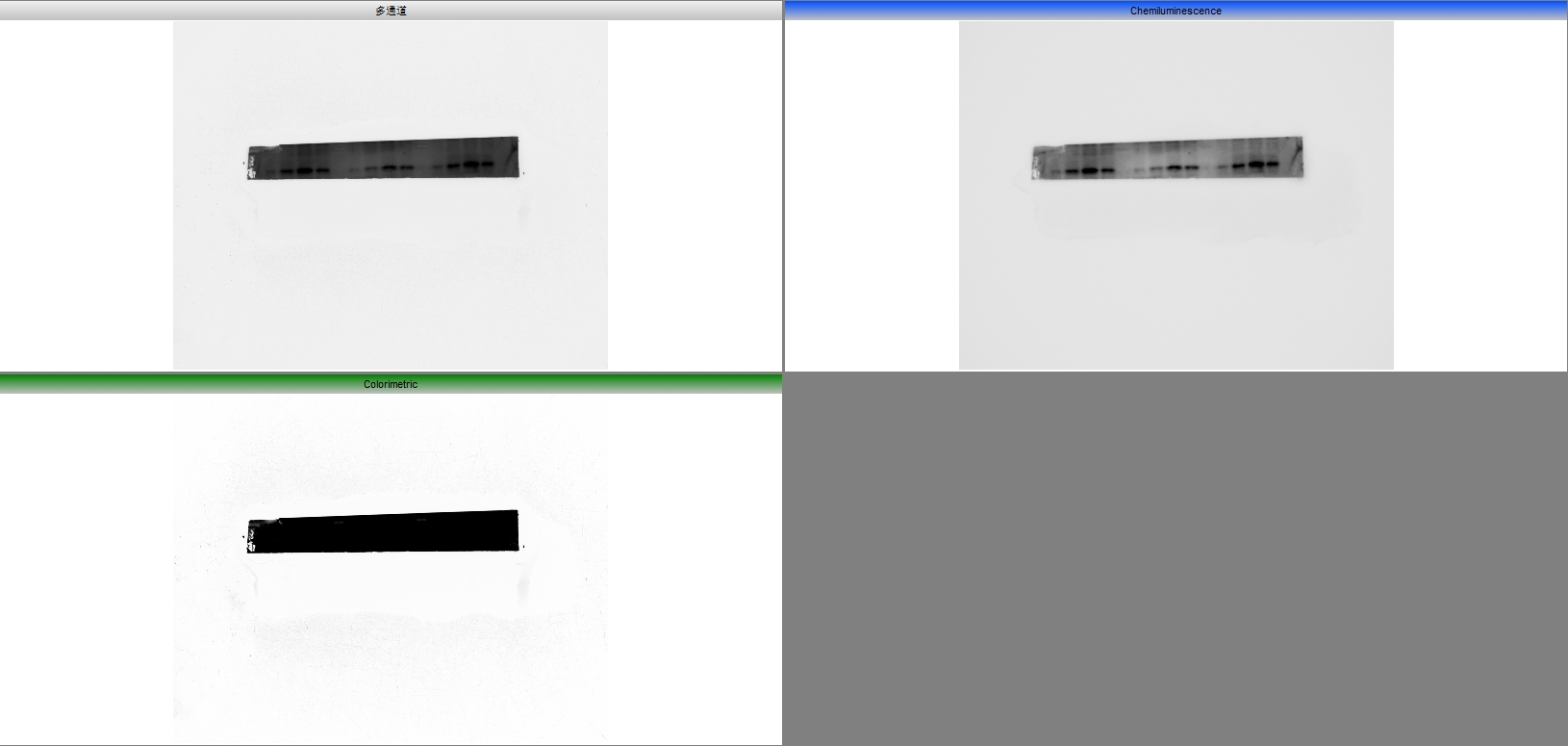

Supplement: Supplementary file 1 [file cancers-14-04809-s001.zip › cancers-1883913-Supplementary File S1. original whole blot/fig7/T24 Cleaved caspase-3.jpg]

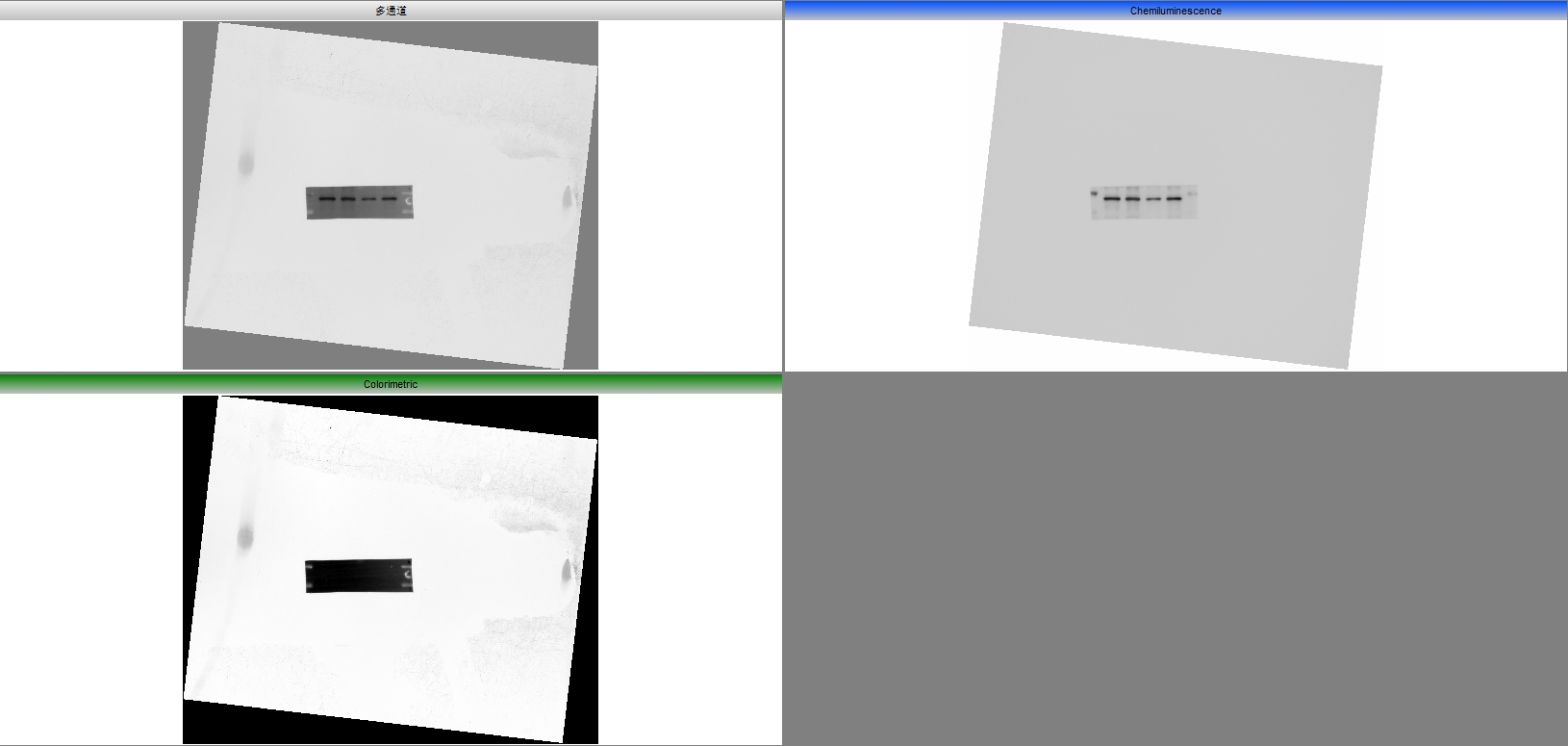

Supplement: Supplementary file 1 [file cancers-14-04809-s001.zip › cancers-1883913-Supplementary File S1. original whole blot/fig7/T24 Cyclin D1 2.jpg]

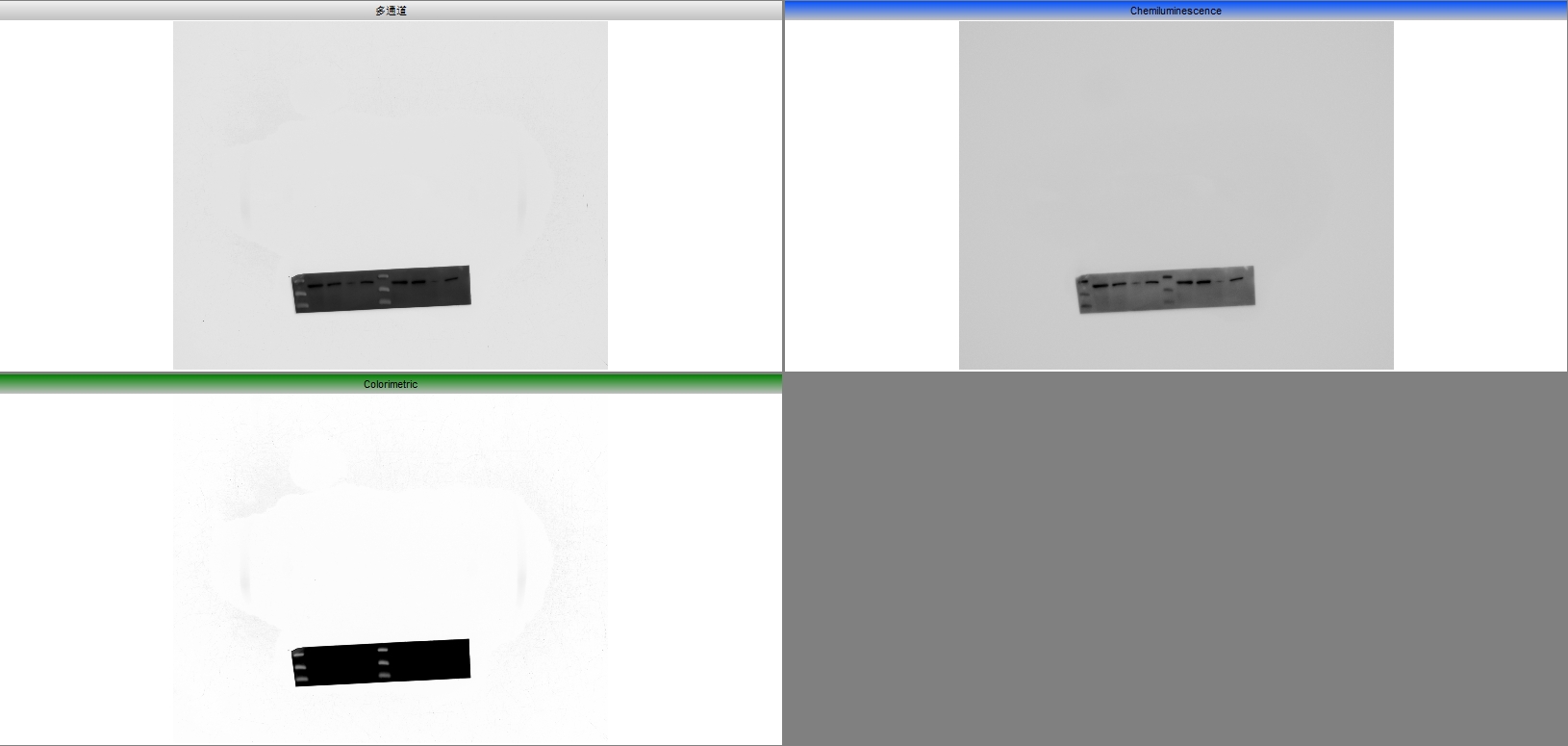

Supplement: Supplementary file 1 [file cancers-14-04809-s001.zip › cancers-1883913-Supplementary File S1. original whole blot/fig7/T24 Cyclin D1.jpg]

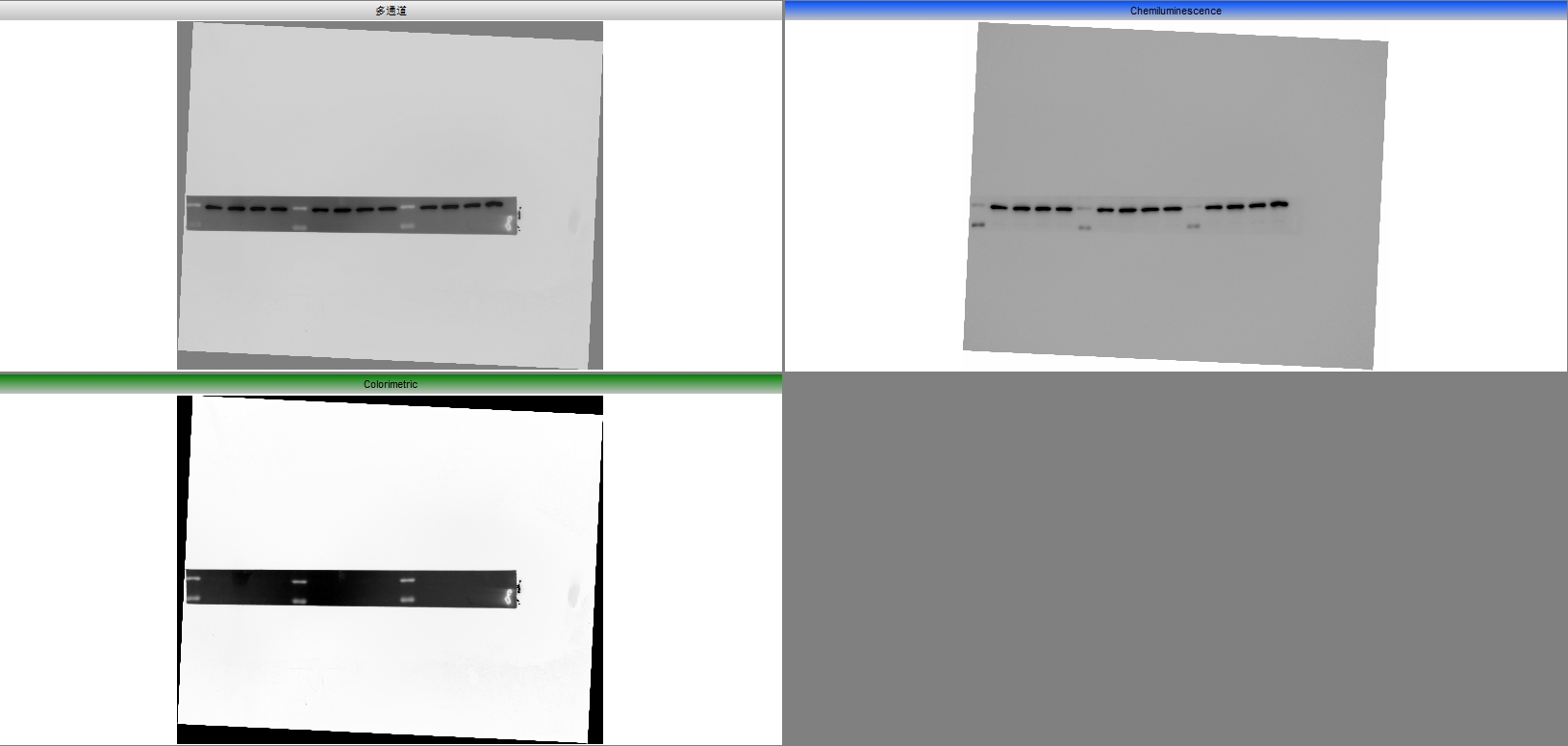

Supplement: Supplementary file 1 [file cancers-14-04809-s001.zip › cancers-1883913-Supplementary File S1. original whole blot/fig7/T24 GAPDH.jpg]

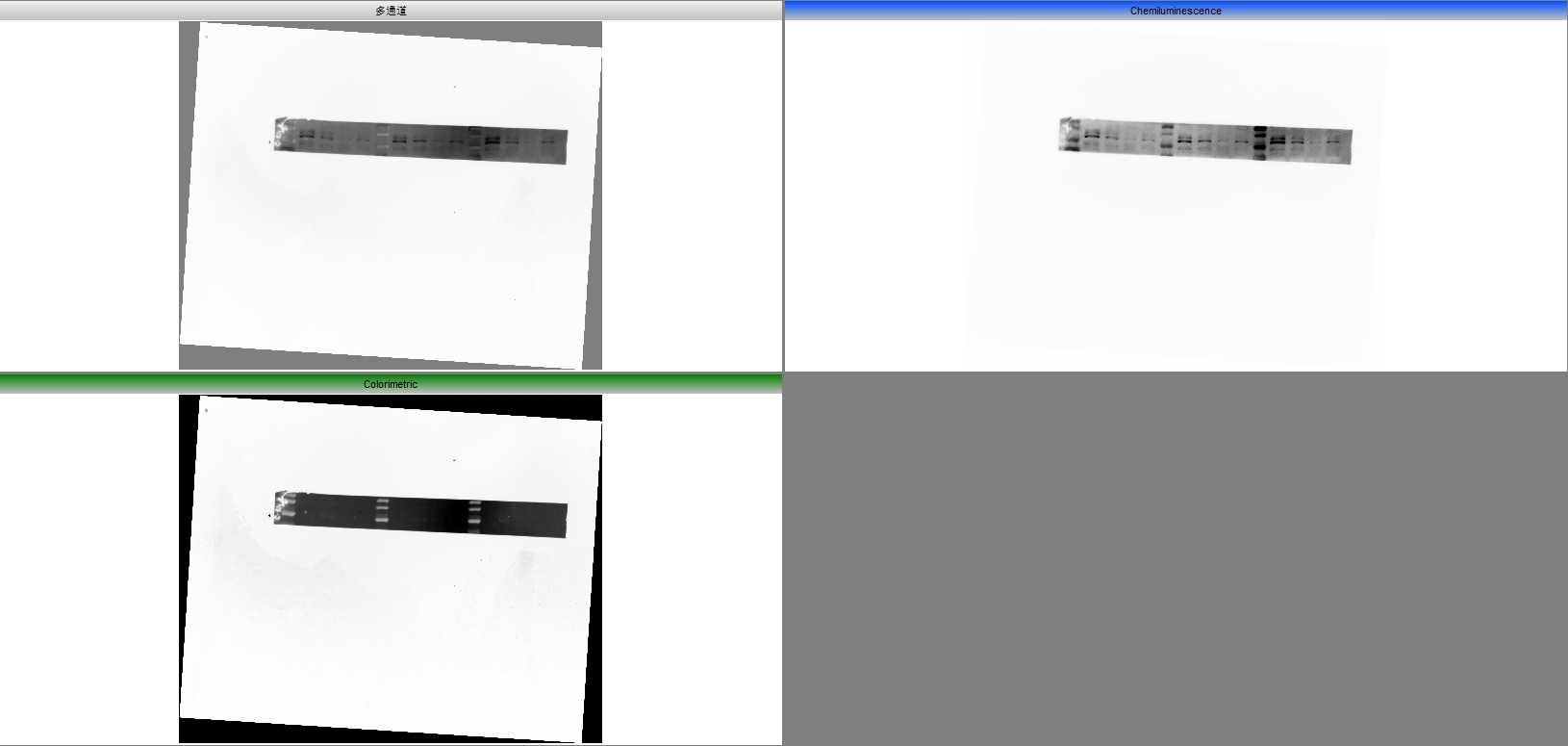

Supplement: Supplementary file 1 [file cancers-14-04809-s001.zip › cancers-1883913-Supplementary File S1. original whole blot/fig7/T24 N-cadhern.jpg]

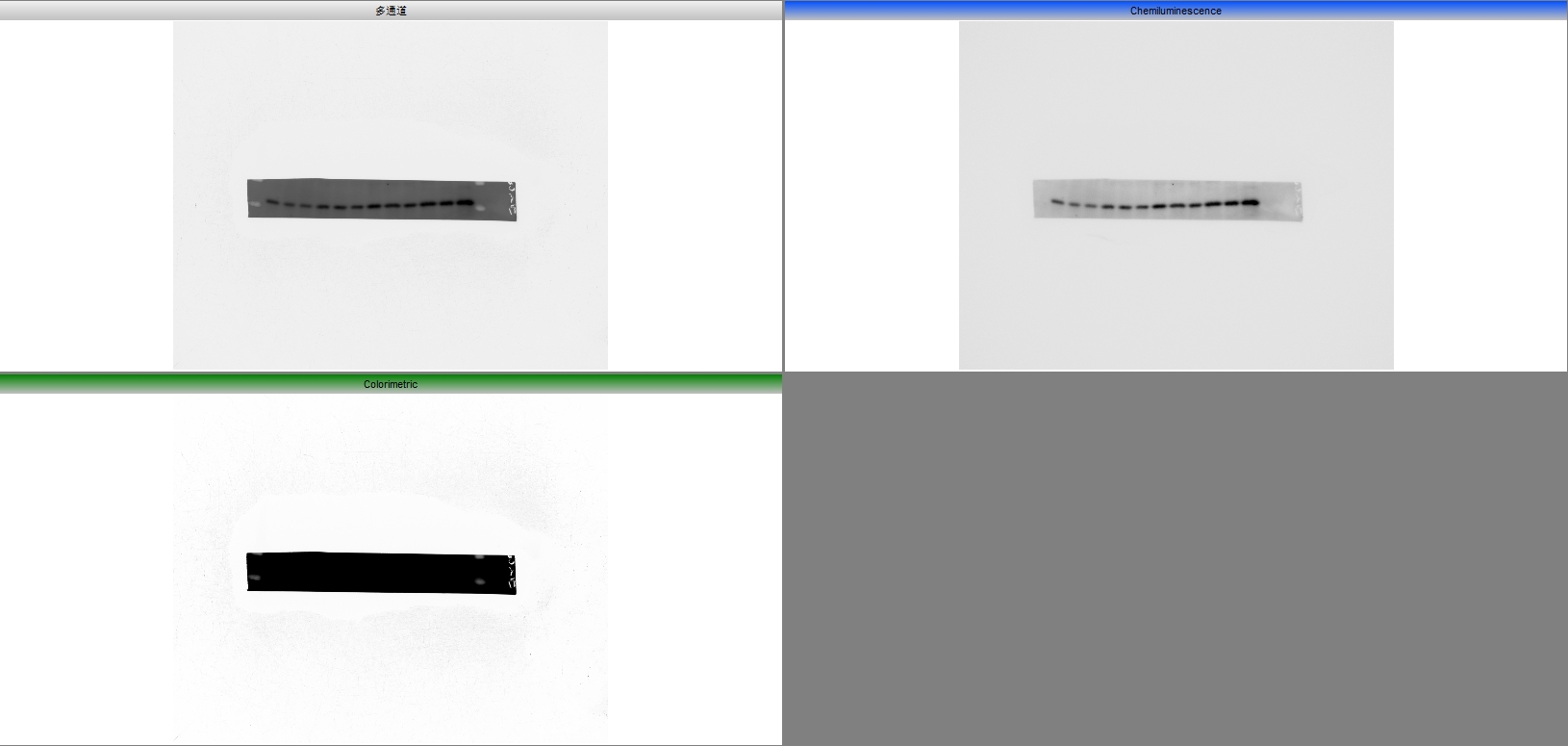

Supplement: Supplementary file 1 [file cancers-14-04809-s001.zip › cancers-1883913-Supplementary File S1. original whole blot/fig8/Cleaved caspase-3.jpg]

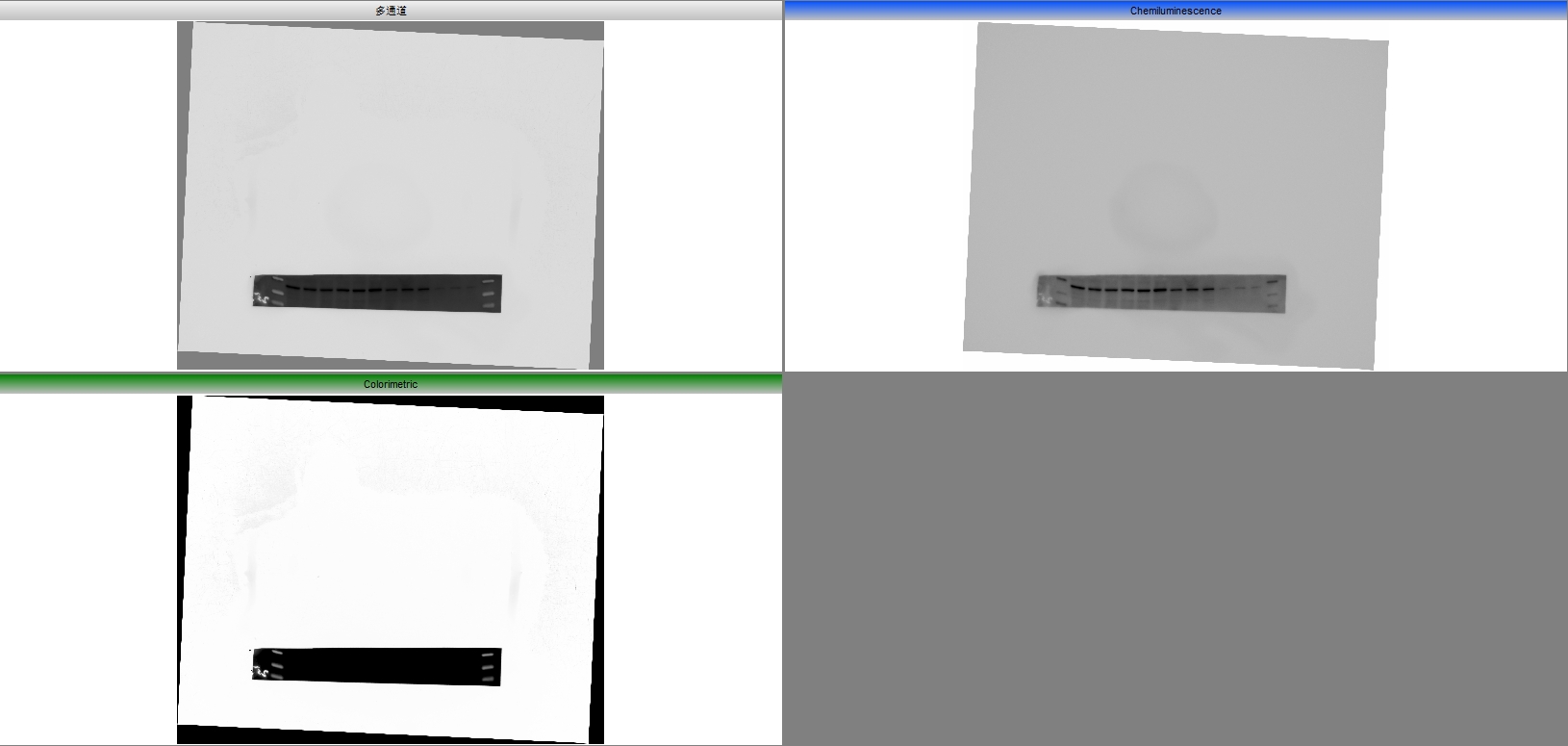

Supplement: Supplementary file 1 [file cancers-14-04809-s001.zip › cancers-1883913-Supplementary File S1. original whole blot/fig8/Cyclin D1.jpg]

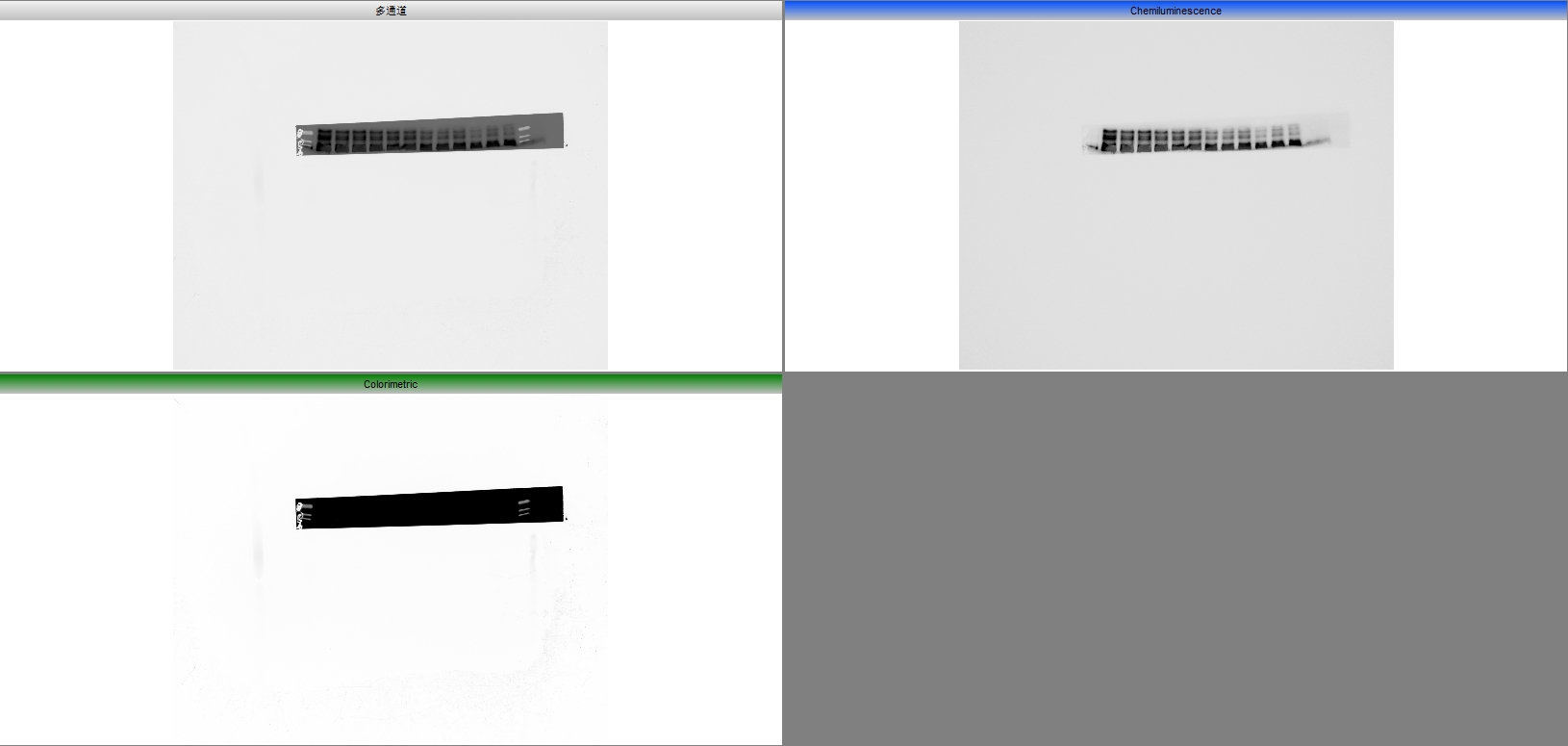

Supplement: Supplementary file 1 [file cancers-14-04809-s001.zip › cancers-1883913-Supplementary File S1. original whole blot/fig8/EGFR.jpg]

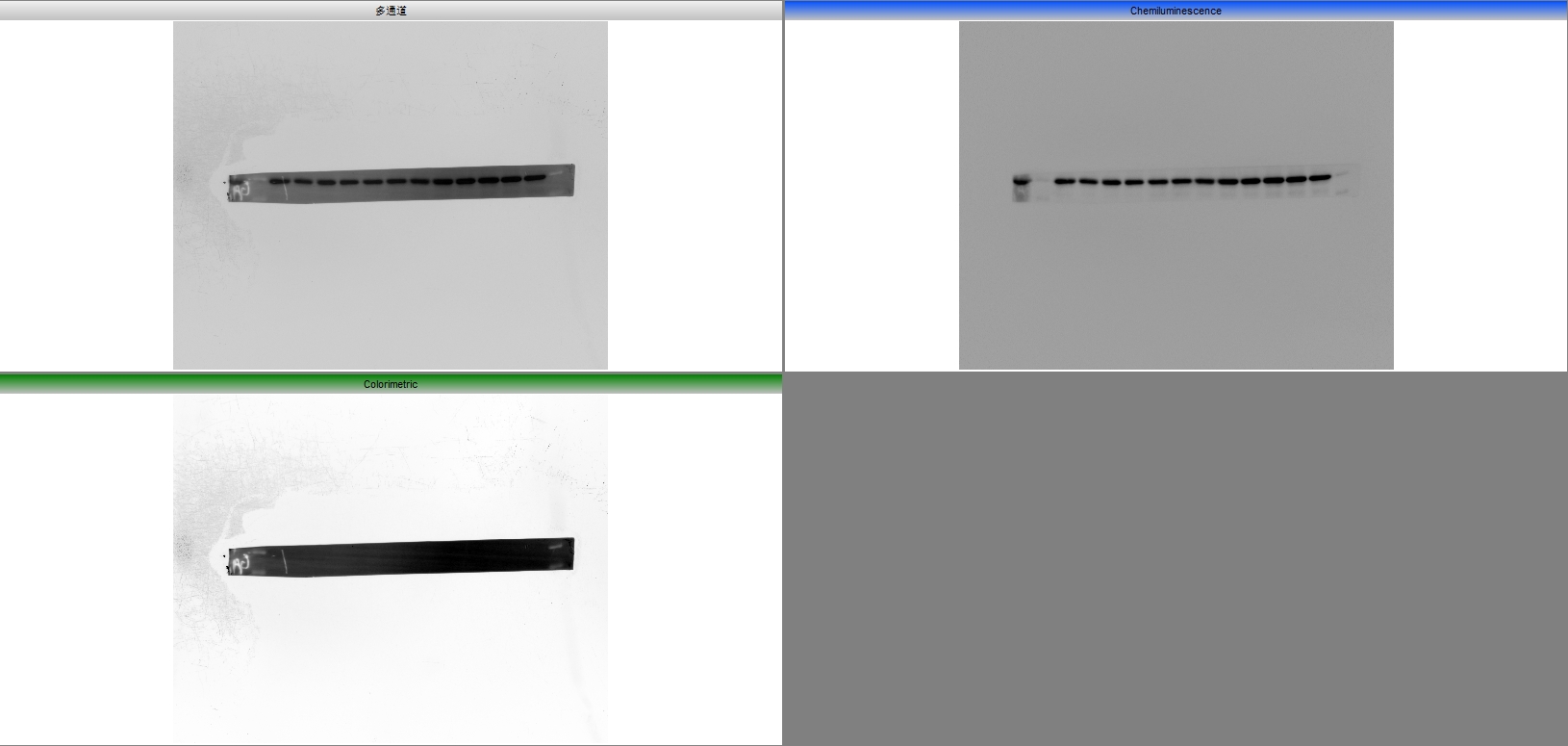

Supplement: Supplementary file 1 [file cancers-14-04809-s001.zip › cancers-1883913-Supplementary File S1. original whole blot/fig8/GAPDH.jpg]

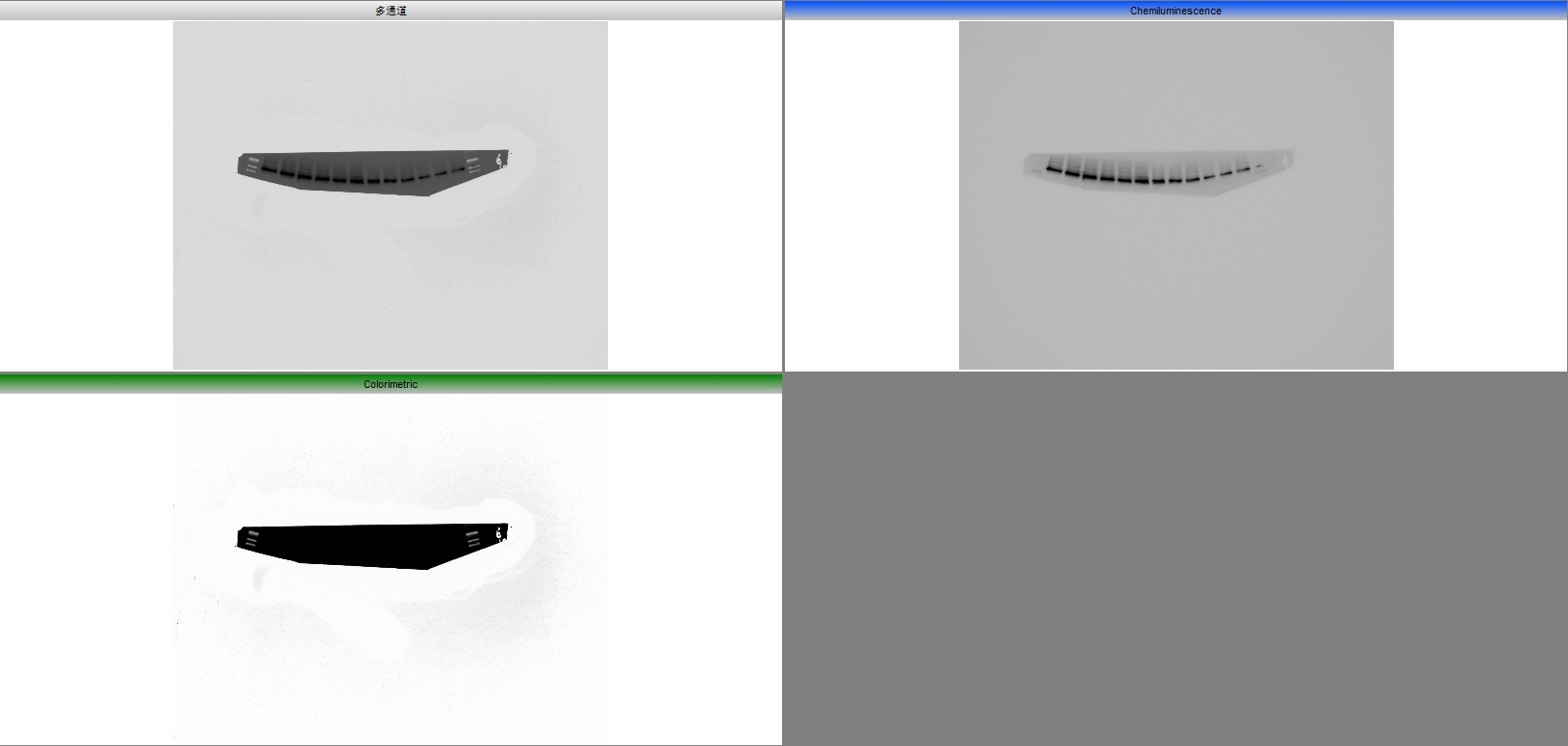

Supplement: Supplementary file 1 [file cancers-14-04809-s001.zip › cancers-1883913-Supplementary File S1. original whole blot/fig8/N-cadhern.jpg]

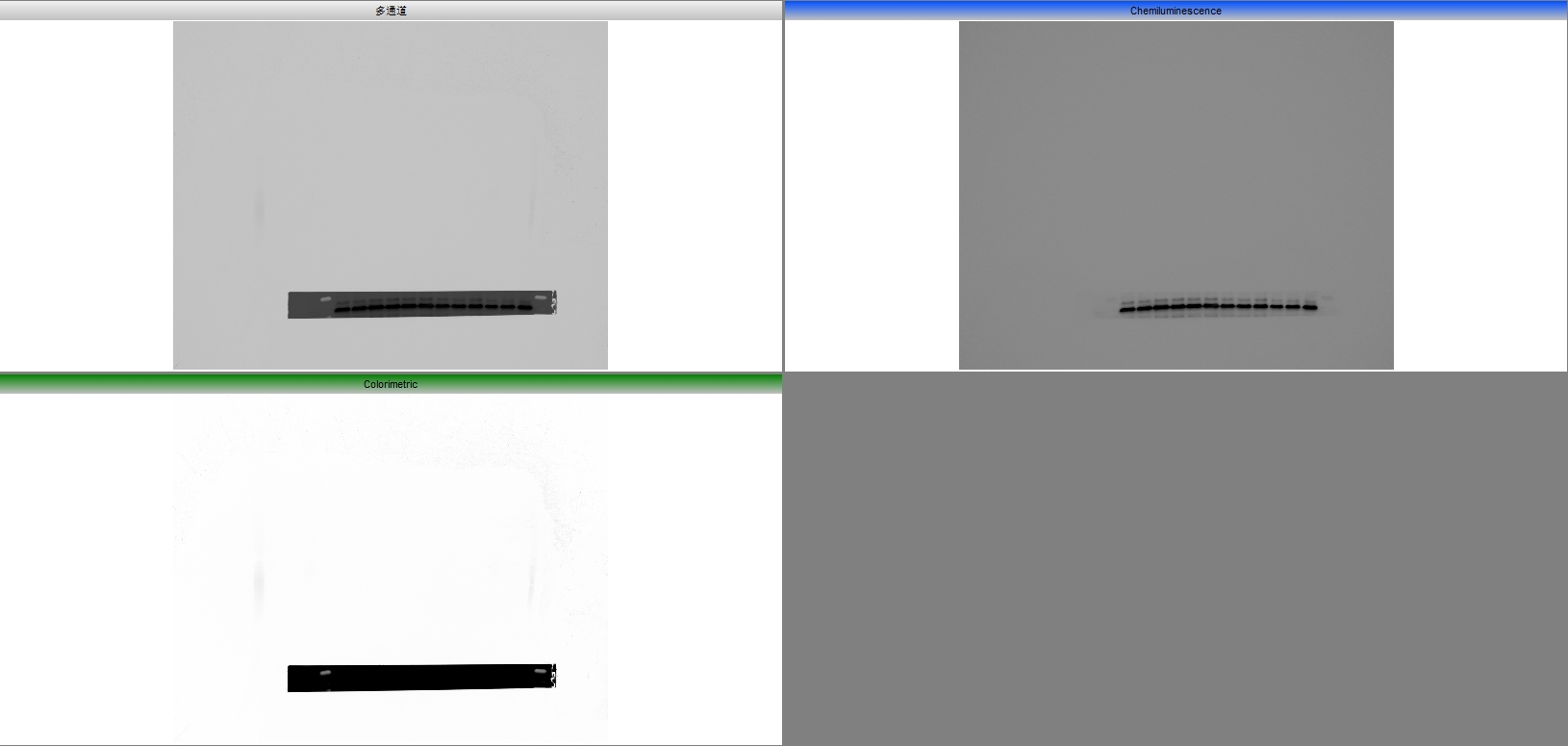

Supplement: Supplementary file 1 [file cancers-14-04809-s001.zip › cancers-1883913-Supplementary File S1. original whole blot/fig8/NF-KB.jpg]

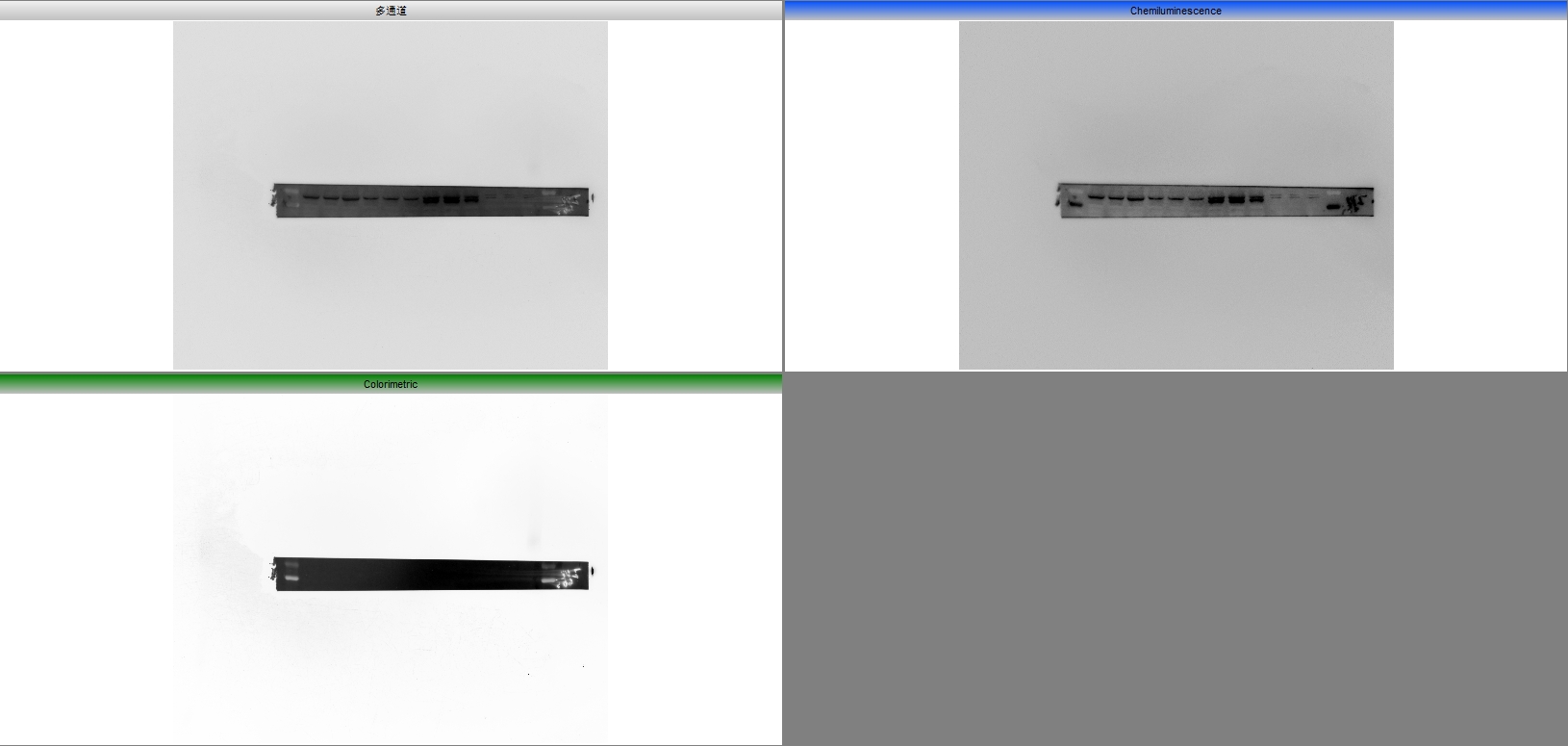

Supplement: Supplementary file 1 [file cancers-14-04809-s001.zip › cancers-1883913-Supplementary File S1. original whole blot/fig8/pEGFR.jpg]

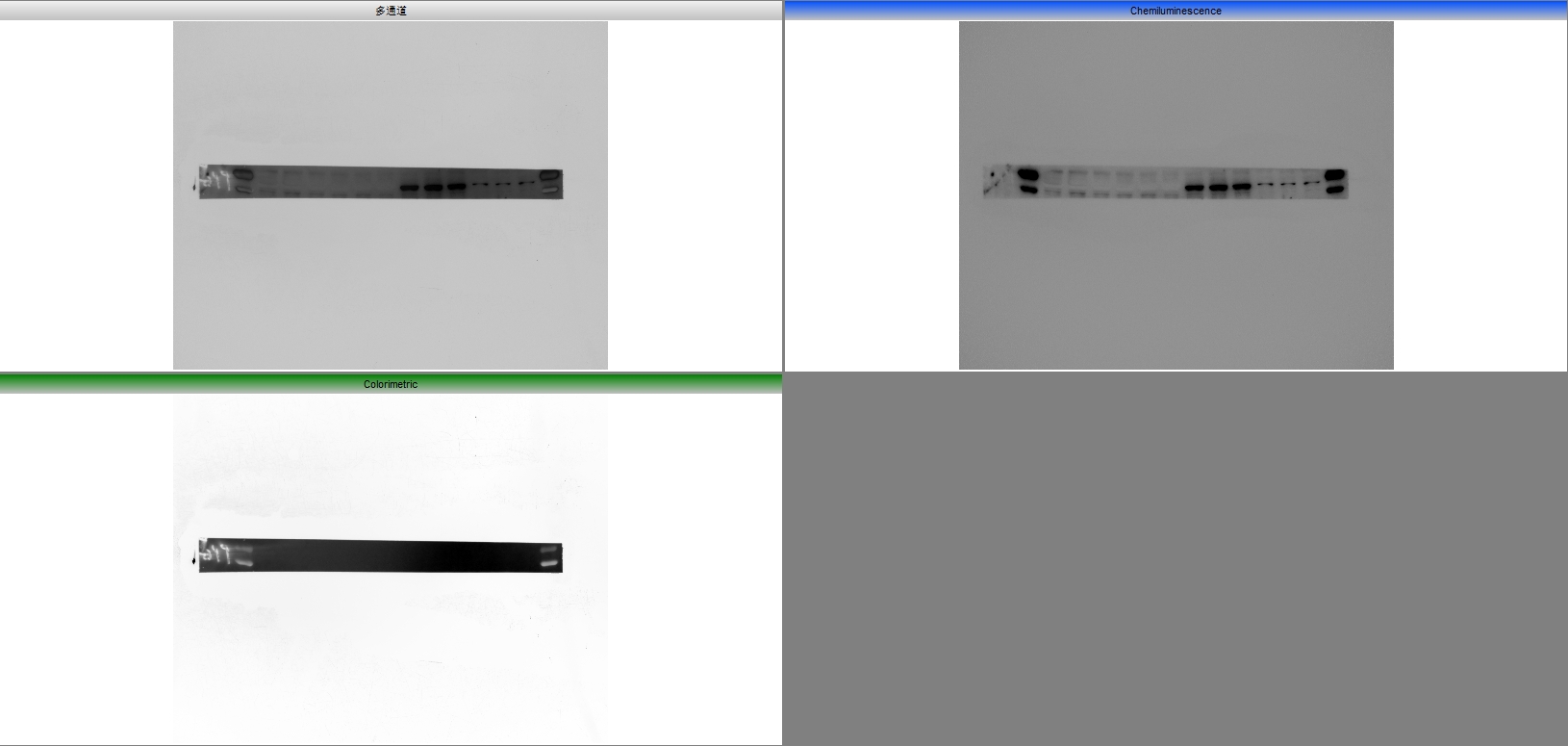

Supplement: Supplementary file 1 [file cancers-14-04809-s001.zip › cancers-1883913-Supplementary File S1. original whole blot/fig8/pNF-KB.jpg]
